# Mild Iron-Catalyzed Oxidative Cross-Coupling of Quinoxalinones with Indoles

**DOI:** 10.3390/molecules29112649

**Published:** 2024-06-04

**Authors:** Hangcheng Ni, Hui Mao, Ying Huang, Yi Lu, Zhenxiang Liu

**Affiliations:** 1 College of Pharmacy, Jinhua Polytechnic, Jinhua 321007, China; 2 Key Laboratory of the Ministry of Education for Advanced Catalysis Materials, Zhejiang Normal University, Jinhua 321004, China

**Keywords:** iron chloride, oxidative coupling, quinoxalinone, indole, 3-(indol-3-yl)quinoxalin-2-one, simplicity, cost effectiveness

## Abstract

Utilizing iron chloride as a Lewis acid catalyst, we developed a straightforward and mild oxidative cross-coupling reaction between quinoxalinones and indoles, yielding a series of versatile 3-(indol-3-yl)quinoxalin-2-one derivatives. This approach allows for the incorporation of a wide array of functional groups into the final products, demonstrating its synthetic versatility. Notably, the method was successfully scaled up to gram-scale reactions while maintaining high yields. Our mechanistic investigation indicates that iron chloride serves as a catalyst to facilitate the formation of key intermediates which subsequently undergo oxidation to afford the desired products. The merits of this protocol include its cost effectiveness, operational simplicity, and the ease of product isolation via filtration.

## 1. Introduction

Iron, an abundant, economical, and low-toxicity metal, plays a pivotal role in organic synthesis. Over the past decade, iron-catalyzed reactions have been successfully applied to a spectrum of organic transformations, including substitution, addition, elimination, oxidation, and reduction [1,2,3]. The utility of iron complexes extends to asymmetric catalysis, a more complex and challenging domain [4]. More recently, the potential of iron complexes has been harnessed in green photocatalytic processes, where intramolecular or intermolecular charge transfer facilitates the generation of reactive radical species [5].

**Scheme 1:**
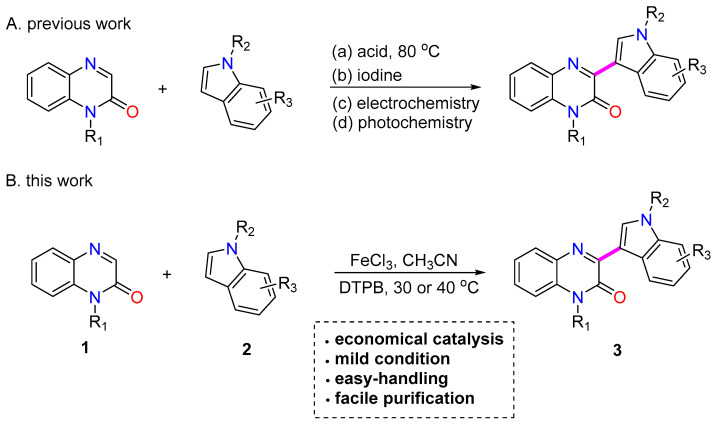
Synthesis of 3-(indol-3-yl)quinoxalin-2-ones via oxidative dehydrogenative coupling reactions of quinoxalinones with indoles.

Quinoxalinones are a significant class of heterocyclic compounds with widespread applications in medicinal chemistry and advanced functional materials [6,7,8,9,10]. Notably, the subclass of 3-(indol-3-yl)quinoxalin-2-one derivatives, which features both a quinoxalinone framework and an indole moiety, has shown remarkable biological activities. These include potent antibacterial properties, inhibition of platelet aggregation, and a significant suppression of human tumor cell proliferation [11,12,13]. Historically, Brønsted acids have been employed as catalysts for the cross-coupling of quinoxalinones with indoles under high-temperature conditions [14]. Molecular iodine has also proven effective for this reaction [15]. With the advent of electrochemistry and photochemistry, these innovative methodologies have been integrated into the reaction system [16,17,18]. For instance, Tang’s group has reported a visible light-promoted cross-coupling of quinoxalinones with indole derivatives, utilizing B(C_6_F_5_)_3_·H_2_O as a Lewis acid catalyst (Scheme 1A) [17].

In this work, we introduce an iron-catalyzed cross-coupling method for the synthesis of 3-(indol-3-yl)quinoxalin-2-one derivatives (Scheme 1B). This approach offers the advantages of a cost-effective catalyst, mild reaction conditions, and straightforward product isolation. Notably, the process allows for the direct filtration and isolation of pure products, eliminating the need for additional purification steps.

## 2. Results and Discussion

As depicted in Table 1, the reaction of quinoxalinone (**1a**) with indole (**2a**, 1.5 equivalents) at 30 °C, catalyzed by 10% FeCl_3_ and using 2.0 equivalents of di-tert-butyl peroxide (DTBP), yielded the desired product (**3a**) with an 81% yield (Entry 1). Utilizing 2.0 equivalents of indole resulted in a higher yield compared to lower amounts, and increasing the indole further did not improve the yield (Entries 2–4). Catalyst optimization demonstrated that a loading of 10% FeCl_3_ was optimal for achieving the highest yield (Entries 5–7). The quantity of DTBP was also screened, confirming that 2.0 equivalents were necessary for the maximum yield (Entries 8–9). Solvent screening revealed that acetonitrile (CH_3_CN) was the most effective medium, with other solvents such as dichloromethane (DCM) yielding product **3a** in moderate yields (Entries 10–11). Additionally, reactions conducted at shorter time resulted in lower yields of **3a**, indicating 24 h was essential for this reaction (Entry 12).

**Figure 1:**
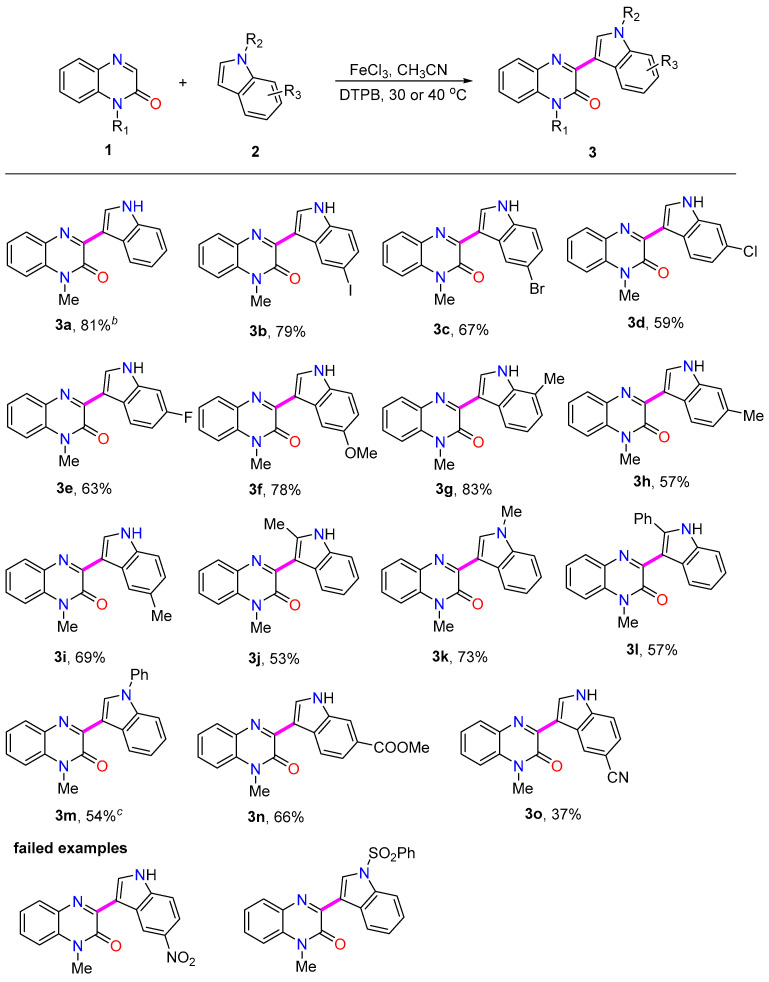
Substrate scope of indoles *^a^*. *^a^* All reactions were conducted at 0.5 mmol scale of 1a in 5.0 mL of CH3CN in a closed flask at 30 °C for 24 h; Isolated yield. *^b^* Reaction at 40 °C for 24 h. *^c^* Reaction conducted at 30 °C for 24 h, then 40 °C for 24 h.

Under the optimal reaction condition, we explored the substrate scope of the oxidative coupling between quinoxalinone (**1a**) and a series of indoles (**2**), with the results summarized in Figure 1. Indoles bearing various functional groups, including alkyl, methoxy, halide and cyanide, were all found to be compatible with our protocol, affording the corresponding products in moderate to good yields (**3a**–**3o**). Halogenated indoles, known for their reactivity in cross-coupling reactions, were smoothly converted into the desired products with good yields (**3b**–**3e**). The position of the substituents on the indole ring had a significant impact on the reaction efficiency (**3f**–**3k**), suggesting that the electronic properties of the three-position of the indoles are crucial for the reaction outcome. More sterically hindered indoles, such as 2-methyl indole and 2-phenyl indole, also successfully underwent the coupling reaction, albeit with moderate yields. Notably, electron-deficient indoles, including methyl *1H*-indole-6-carboxylate and *1H*-indole-5-carbonitrile, were viable substrates, yielding the desired products (**3n** and **3o**) with somewhat lower yields. However, when more electron-deficient indoles were used as substrates, the reaction resulted in only trace amounts of the product, with the starting material **1a** remaining unreacted.

**Figure 2:**
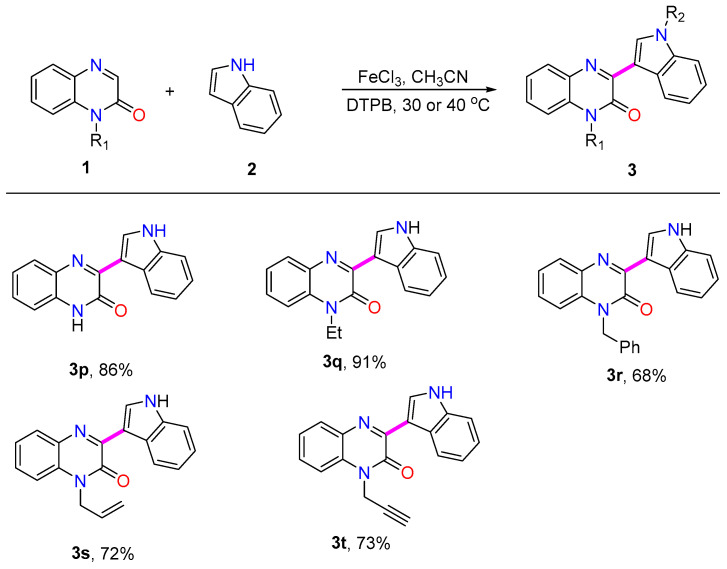
Substrate scope of quinoxalinones *^a^*. *^a^* All reactions were conducted at 0.5 mmol scale of **1** in 5.0 mL of CH_3_CN in a closed flask at 30 °C for 24 h, then 40 °C for 24 h; Isolated yield.

The substrate scope concerning N-substituted quinoxalinones (**1**) was then investigated, as detailed in Figure 2. N-substituted quinoxalinones (**1b**–**1f**) featuring a range of important and potentially reactive functional groups, including alkenyl and alkynyl moieties, were found to be well-tolerated under the reaction conditions. Elevated temperature was required for the full conversion of reaction intermediate. It is noteworthy that the commercially available starting material, quinoxalin-2(*1H*)-one (**1b**), could be efficiently converted to the desired product with an 86% yield upon increasing the temperature to ensure complete conversion of the intermediate to the final product. Interestingly, N-substituted quinoxalinones with the less stable alkenyl and alkynyl groups also successfully yielded products **3s** and **3t** in relatively good yields.

**Scheme 2:**
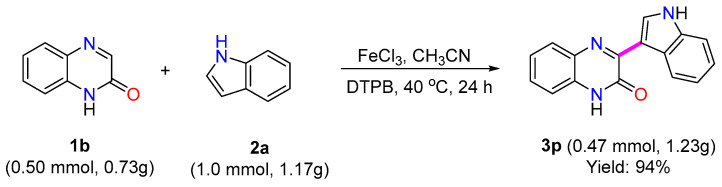
Gram scale reaction.

To demonstrate the practicality of the iron salt-catalyzed oxidative coupling reaction, a gram-scale synthesis was conducted under the standard condition. Gratifyingly, the reaction, employing commercially available quinoxalin-2(*1H*)-one (**1b**) and indole (**2a**) as substrates, was readily scalable to the gram scale, yielding the desired product **3p** with an exceptional isolated yield of 94%. It is particularly noteworthy that the product could be directly isolated via simple filtration of the reaction mixture, thereby eliminating the need for additional purification steps. This straightforward approach offers a cost-effective and convenient method for the large-scale synthesis of 3-(*1H*-indol-3-yl)quinoxalin-2(*1H*)-one derivatives, as illustrated in Scheme 2.

**Scheme 3:**
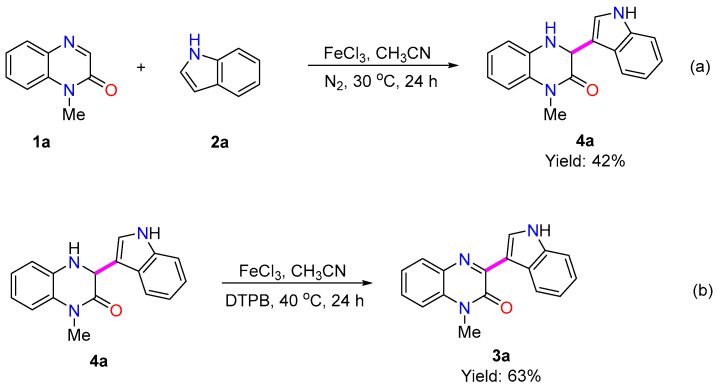
Control experiments: (**a**) Isolation of reaction intermediate; (**b**) Conversion of intermediate to product.

To elucidate the reaction mechanism, a series of control experiments were performed, as depicted in Scheme 3. When the reaction was carried out under a nitrogen atmosphere (Scheme 3a), the intermediate **4a** was identified as the predominant product, which was isolated in a 42% yield by filtration of the reaction mixture. The structure of intermediate **4a** was confirmed through ^1^H NMR and high-resolution mass spectrometry (HRMS) analyses. Notably, **4a** could be further oxidized to the desired product **3a** in a 63% yield upon continuation of the reaction under the standard condition. This observation implies that a Lewis acid-catalyzed Friedel–Crafts-type reaction is likely the initial step, leading to the formation of intermediate **4a** [14,17]. Furthermore, the observed lower efficiency with electron-deficient indoles (Figure 1, entries featuring 5-nitro-*1H*-indole and *1H*-indole-5- carbonitrile) provides additional support for the proposed iron salt-catalyzed Friedel–Crafts-type electrophilic substitution reaction mechanism.

**Scheme 4:**
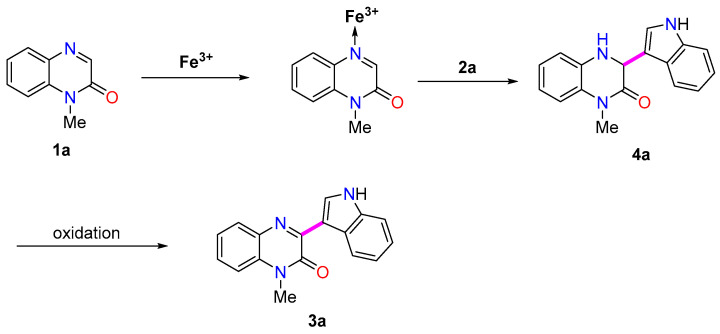
Proposed mechanism.

Based on the aforementioned findings and the relevant literature [14,17], we propose a plausible mechanism for the transformation of quinoxalinones (**1**) with indoles (**2**) to afford a variety of 3-(indol-3-yl)quinoxalinones (**3**), as illustrated in Scheme 4. Initially, quinoxalinone (**1a**) is coordinated to the Lewis acid (FeCl_3_), resulting in the formation of a cationic intermediate. Subsequently, this cationic intermediate undergoes an electrophilic Friedel–Crafts-type reaction with an electron-rich indole (**2a**), leading to the generation of intermediate **4a**. The final step involves the further oxidation of intermediate **4a** by di-tert-butyl peroxide (DTBP), ultimately yielding the desired product **3a**.

In conclusion, we developed a straightforward and mild oxidative cross-coupling method for the synthesis of versatile 3-(indol-3-yl)quinoxalinone derivatives, utilizing iron chloride as a Lewis acid catalyst. This approach allows for the incorporation of various functional groups into the products through the use of a diverse range of indoles and quinoxalin-2(*1H*)-one derivatives. Our mechanistic studies indicate that iron chloride serves as an effective Lewis acid catalyst, facilitating the reaction process. The protocol is distinguished by its cost effectiveness and ease of operation, with product isolation achievable through simple filtration. Furthermore, the method is successfully scaled up to gram-scale reactions with high yields, employing commercially available starting materials. This scalability provides a simple, economical and convenient route for the large-scale synthesis of a variety of 3-(indol-3-yl)quinoxalinone derivatives.

## 3. Materials and Methods

### 3.1. General Information

^1^H NMR and ^13^C NMR spectra were recorded on a Bruker AVANCE NEO 400 MHZ (Karlsruhe, Germany). Spectra were calibrated relative to resonances of the deuterated solvents for proton and carbon chemical shifts: DMSO (*δ* = 2.50 for ^1^H NMR and *δ* = 39.50 for ^13^C NMR), CDCl_3_ (*δ* = 7.26 for ^1^H NMR and *δ* = 77.16 for ^13^C NMR). Data are reported as follows: chemical shift δ/ppm, integration (1H only), multiplicity (s = singlet, d = doublet, t =triplet, dd = doublet of doublets, m = multiplet; ^13^C signals are singlets unless otherwise stated), coupling constants *J* in Hz. HRMS spectra were obtained on a waters G2-XS Qtof (Milford, MA, USA). High-performance liquid Chromatographic (HPLC) was carried out on a Shimadzu LC-20ADxr (Kyoto, Japan). The melting point was recorded on Shanghai INESA Physico-Optical Instrument INESA SGM X-4A (Shanghai, China). Compounds **1a** and **1c**–**1f** were prepared using literature methods [19,20]. All other chemicals were purchased from Chemical Co. (Shanghai Titan Scientific Co., Ltd., Shanghai, China, Anhui Zesheng Technology Co., Ltd., Anqing, China, etc.) and used as received unless otherwise specified.

### 3.2. General Procedure for the Synthesis of Starting Materials (***1a*** and ***1c**–**1f***) [19,20]

A typical procedure is as follows: To a stirred solution of quinoxalin-2(*1H*)-ones (3 mmol) in DMF (10 mL), the corresponding halide (1.6 equiv) and potassium carbonate (1.2 equiv) were added at room temperature; the mixture was stirred overnight. Then, the resulting mixture was supplemented with water (50 mL) and extracted with ethyl acetate (50 mL) for three times. The combined organic layers were dried over Na_2_SO_4_, filtered and evaporated under reduced pressure. The residue was purified by column chromatography on silica gel to produce the desired product **1**. See Supporting Information for more details including the chemical structural formulae and ^1^H NMR and ^13^C NMR spectra of the products.

### 3.3. General Procedure for the Synthesis of Product (***3a**–**3t***)

A Schlenk tube equipped with a magnetic stir bar was charged with **1** (0.5 mmol) and **2** (1.0 mmol, 2.0 equiv). FeCl_3_ solution (0.01 mmol/mL in CH_3_CN, 5 mL) and di-tert-butyl peroxide (DTBP, 1.0 mmol, 2.0 equiv) were added. Then, the reaction mixture was stirred at 30 or 40 °C for 24 h. After that, the resulting mixture was analyzed by HPLC and handled in two ways: (1) For the reaction with the precipitant, the mixture was filtered to produce product **3,** which was washed with a small amount of acetonitrile. The solution was further condensed to precipitate the product, and the mixture was filtered. (2) For the reaction without the precipitant, the reaction solution was removed under reduced pressure with a rotary evaporator. The crude residue was purified by silica gel column chromatography with ethyl acetate and petrol ether (1:4) as eluent to produce pure product **3**.

### 3.4. Characterization Data of Products

The chemical structural formulae and ^1^H NMR and ^13^C NMR spectra of products **3a**–**3t** can be seen in Supporting Information.

*3-(1H-indol-3-yl)-1-methylquinoxalin-2(1H)-one* (**3a**):
Isolated through filtration to produce yellow solid. Yield: 81%. ^1^H NMR (400 MHz, DMSO-*d*_6_) δ 11.79 (s, 1H), 9.03–8.85 (m, 2H), 7.92 (d, *J* = 7.9 Hz, 1H), 7.61–7.48 (m, 3H), 7.46–7.33 (m, 1H), 7.33–7.18 (m, 2H), 3.74 (s, 3H). ^13^C NMR (101 MHz, DMSO-*d*_6_) δ 153.66, 150.62, 136.29, 133.18, 132.96, 131.49, 128.36, 128.25, 126.31, 123.42, 123.02, 122.53, 120.98, 114.42, 111.88, 111.41, 29.09. This is a known structure. These data are similar to the reported ones [14].
*3-(5-iodo-1H-indol-3-yl)-1-methylquinoxalin-2(1H)-one* (**3b**):
Isolated through filtration to produce yellow solid. M.P.: >300 °C. Yield: 79%. ^1^H NMR (400 MHz, DMSO-*d*_6_) δ 11.93 (s, 1H), 9.23 (s, 1H), 8.89 (s, 1H), 7.85 (d, *J* = 7.9 Hz, 1H), 7.60–7.47 (m, 3H), 7.47–7.32 (m, 2H), 3.71 (s, 3H). ^13^C NMR (101 MHz, DMSO-*d*_6_) δ 153.54, 150.22, 135.39, 133.80, 132.72, 131.55, 131.26, 130.51, 128.75, 128.54, 128.34, 123.53, 114.50, 114.36, 110.57, 85.43, 29.10. HRMS (ESI): *m/z* calcd for C_17_H_12_N_3_OI [M + Na]^+^: 423.9923. Found: 423.9923.
*3-(5-bromo-1H-indol-3-yl)-1-methylquinoxalin-2(1H)-one* (**3c**):
Isolated through filtration to produce yellow solid. Yield: 67%. ^1^H NMR (400 MHz, DMSO-*d*_6_) δ 11.96 (s, 1H), 9.01 (s, 1H), 8.93 (s, 1H), 7.87 (d, *J* = 7.9 Hz, 1H), 7.72–6.40 (m, 5H), 3.71 (s, 3H). ^13^C NMR (101 MHz, DMSO-*d*_6_) δ 153.55, 150.23, 135.04, 134.27, 132.73, 131.56, 128.57, 128.40, 127.99, 125.05, 125.00, 123.53, 114.51, 113.94, 113.78, 110.90, 29.11. This is a known structure. These data are similar to the reported ones [14].
*3-(5-chloro-1H-indol-3-yl)-1-methylquinoxalin-2(1H)-one* (**3d**):
Isolated through filtration to produce yellow solid. Yield: 59%. ^1^H NMR (400 MHz, DMSO-*d*_6_) δ 11.86 (s, 1H), 8.92 (s, 1H), 8.85 (s, 1H), 7.89 (s, 1H), 7.53 (m, 3H), 7.37 (s, 1H), 7.23 (s, 1H), 3.71 (s, 3H). ^13^C NMR (101 MHz, DMSO-*d*_6_) δ 153.53, 150.23, 136.80, 133.96, 132.78, 131.58, 128.52, 128.45, 127.09, 125.06, 124.26, 123.43, 121.11, 114.44, 111.58, 111.45, 29.08. This is a known structure. These data are similar to the reported ones [14].
*3-(6-fluro-1H-indol-3-yl)-1-methylquinoxalin-2(1H)-one* (**3e**):
Isolated through filtration to produce yellow solid. M.P.: 261.0–263.0 °C. Yield: 63%. ^1^H NMR (400 MHz, DMSO-*d*_6_) δ 11.81 (s, 1H), 8.97–8.82 (m, 2H), 7.89 (d, *J* = 7.9 Hz, 1H), 7.52 (t, *J* = 2.8 Hz, 2H), 7.37 (s, 1H), 7.30 (d, *J* = 9.7 Hz, 1H), 7.07 (t, *J* = 9.3 Hz, 1H), 3.71 (d, *J* = 2.0 Hz, 3H). ^13^C NMR (101 MHz, DMSO-*d*_6_) δ 159.25 (d, *J* = 236.4 Hz), 153.55, 150.30, 136.40 (d, *J* = 12.5 Hz), 133.73 (d, *J* = 2.6 Hz), 132.82, 131.58, 128.43, 128.42, 124.14 (d, *J* = 9.6 Hz), 123.42, 123.01, 114.43, 111.45, 109.07 (d, *J* = 23.4 Hz), 98.06 (d, *J* = 25.7 Hz), 29.08. HRMS (ESI): *m/z* calcd for C_17_H_12_N_3_OF [M + H]^+^: 294.1043. Found: 294.1046.
*3-(5-methoxy-1H-indol-3-yl)-1-methylquinoxalin-2(1H)-one* (**3f**):
Isolated through filtration to produce yellow solid. Yield: 78%. ^1^H NMR (400 MHz, DMSO-*d*_6_) δ 11.68 (s, 1H), 8.88 (s, 1H), 8.47 (s, 1H), 7.90 (d, *J* = 7.9 Hz, 1H), 7.53 (s, 2H), 7.47–7.33 (m, 2H), 6.89 (d, *J* = 8.7 Hz, 1H), 3.88 (s, 3H), 3.72 (s, 3H). ^13^C NMR (101 MHz, DMSO-*d*_6_) δ 154.87, 153.67, 150.68, 133.52, 132.94, 131.42, 131.17, 128.31, 128.14, 126.99, 123.45, 114.44, 112.48, 112.08, 111.13, 105.06, 55.24, 29.09. This is a known structure. These data are similar to the reported ones [14].
*3-(7-methyl-1H-indol-3-yl)-1-methylquinoxalin-2(1H)-one* (**3g**):
Isolated through filtration to produce yellow solid. M.P.: 289.0–291.0 °C. Yield: 83%. ^1^H NMR (400 MHz, DMSO-*d*_6_) δ 11.78 (s, 1H), 8.92 (s, 1H), 8.73 (d, *J* = 8.0 Hz, 1H), 7.91 (d, *J* = 7.9 Hz, 1H), 7.53 (d, *J* = 3.9 Hz, 2H), 7.46–7.34 (m, 1H), 7.14 (t, *J* = 7.6 Hz, 1H), 7.04 (d, *J* = 7.1 Hz, 1H), 3.73 (s, 3H), 2.53 (s, 3H). ^13^C NMR (101 MHz, DMSO-*d*_6_) δ 153.70, 150.61, 135.71, 132.96, 132.86, 131.47, 128.36, 128.23, 126.08, 123.41, 123.17, 121.19, 120.93, 120.65, 114.41, 111.81, 29.08, 16.76. HRMS (ESI): *m/z* calcd for C_18_H_15_N_3_O [M + H]^+^: 290.1293. Found: 290.1298.
*3-(6-methyl-1H-indol-3-yl)-1-methylquinoxalin-2(1H)-one* (**3h**):
Isolated through filtration to produce yellow solid. Yield: 57%. ^1^H NMR (400 MHz, DMSO-*d*_6_) δ 11.65 (s, 1H), 8.85 (d, *J* = 2.9 Hz, 1H), 8.74 (d, *J* = 8.1 Hz, 1H), 7.90 (d, *J* = 7.9 Hz, 1H), 7.53 (s, 1H), 7.50 (d, *J* = 9.1 Hz, 1H), 7.42–7.34 (m, 1H), 7.30 (s, 1H), 7.06 (d, *J* = 8.2 Hz, 1H), 3.72 (s, 3H), 2.44 (s, 3H). ^13^C NMR (101 MHz, DMSO-*d*_6_) δ 153.64, 150.60, 136.71, 132.99, 132.73, 131.68, 131.46, 128.32, 128.15, 124.18, 123.39, 122.72, 122.63, 114.40, 111.70, 111.40, 29.07, 21.36. This is a known structure. These data are similar to the reported ones [14].
*3-(5-methyl-1H-indol-3-yl)-1-methylquinoxalin-2(1H)-one* (**3i**):
Isolated through filtration to produce yellow solid. M.P.: >300 °C. Yield: 69%. ^1^H NMR (400 MHz, DMSO-*d*_6_) δ 11.67 (s, 1H), 8.88 (d, *J* = 2.6 Hz, 1H), 8.71 (s, 1H), 7.93 (d, *J* = 7.9 Hz, 1H), 7.54 (d, *J* = 2.2 Hz, 2H), 7.39 (d, *J* = 6.0 Hz, 2H), 7.07 (d, *J* = 8.2 Hz, 1H), 3.73 (s, 3H). ^13^C NMR (101 MHz, DMSO-*d*_6_) δ 153.67, 150.67, 134.60, 133.22, 132.99, 131.43, 129.63, 128.35, 128.12, 126.57, 123.97, 123.38, 122.80, 114.40, 111.51, 111.02, 29.06, 21.66. HRMS (ESI): *m/z* calcd for C_18_H_15_N_3_O [M + H]^+^: 290.1293. Found: 290.1295.
*3-(2-methyl-1H-indol-3-yl)-1-methylquinoxalin-2(1H)-one* (**3j**):
Isolated through silica gel column chromatography with ethyl acetate and petrol ether (1:4) to produce yellow solid. The yellow solid was washed with petrol ether. Yield: 69%. ^1^H NMR (400 MHz, DMSO-*d*_6_) δ 11.49 (s, 1H), 7.80 (d, *J* = 8.0 Hz, 1H), 7.75 (d, *J* = 7.9 Hz, 1H), 7.62–7.52 (m, 2H), 7.41–7.31 (m, 2H), 7.07 (t, *J* = 7.8 Hz, 1H), 7.03–6.97 (m, 1H), 3.71 (s, 3H), 2.55 (s, 3H). ^13^C NMR (101 MHz, DMSO-*d*_6_) δ 154.01, 153.14, 139.29, 135.08, 132.81, 132.53, 129.02, 128.65, 127.96, 123.30, 120.83, 120.80, 119.49, 114.42, 110.59, 109.48, 29.28, 14.24. This is a known structure. These data are similar to the reported ones [14].
*3-(1-methyl-1H-indol-3-yl)-1-methylquinoxalin-2(1H)-one* (**3k**):
Isolated through filtration to produce yellow solid. Yield: 73%. ^1^H NMR (400 MHz, DMSO-*d*_6_) δ 8.95–8.88 (m, 2H), 7.92 (d, *J* = 7.4 Hz, 1H), 7.56 (d, *J* = 7.2 Hz, 3H), 7.44–7.36 (m, 1H), 7.35–7.25 (m, 2H), 3.93 (s, 3H), 3.75 (s, 3H). ^13^C NMR (101 MHz, DMSO-*d*_6_) δ 153.60, 150.32, 136.98, 136.87, 132.97, 131.49, 128.37, 128.31, 126.78, 123.48, 123.14, 122.63, 121.31, 114.48, 110.36, 110.25, 33.07, 29.11. This is a known structure. These data are similar to the reported ones [14].
*3-(2-phenyl-1H-indol-3-yl)-1-methylquinoxalin-2(1H)-one* (**3l**):
Isolated through silica gel column chromatography with ethyl acetate and petrol ether (1:4) to produce yellow solid. Yield: 57%. ^1^H NMR (400 MHz, DMSO-*d*_6_) δ 11.84 (s, 1H), 7.78 (d, *J* = 7.9 Hz, 1H), 7.70–7.44 (m, 6H), 7.42–7.28 (m, 4H), 7.18 (t, *J* = 7.2 Hz, 1H), 7.07 (t, *J* = 7.5 Hz, 1H), 3.59 (s, 3H). ^13^C NMR (101 MHz, DMSO-*d*_6_) δ 153.72, 153.56, 139.19, 135.96, 133.14, 133.05, 132.71, 129.74, 129.02, 128.57, 128.40, 127.86, 127.81, 123.35, 122.08, 120.33, 119.99, 114.61, 111.45, 109.13, 29.28. This is a known structure. These data are similar to the reported ones [14].
*3-(1-phenyl-1H-indol-3-yl)-1-methylquinoxalin-2(1H)-one* (**3m**):
Isolated through filtration to produce yellow solid. Yield: 54%. ^1^H NMR (400 MHz, DMSO-*d*_6_) δ 9.09 (s, 1H), 9.03 (d, *J* = 7.4 Hz, 1H), 8.00 (d, *J* = 7.9 Hz, 1H), 7.73–7.57 (m, 7H), 7.56–7.48 (m, 1H), 7.42–7.31 (m, 2H), 3.76 (s, 3H). ^13^C NMR (101 MHz, DMSO-*d*_6_) δ 153.68, 144.99, 138.26, 135.79, 134.97, 132.84, 131.72, 130.14, 129.01, 128.76, 127.67, 127.40, 124.52, 123.76, 123.65, 122.25, 114.66, 112.78, 110.74, 29.24. This is a known structure. These data are similar to the reported ones [18].
*methyl 3-(4-methyl-3-oxo-3,4-dihydroquinoxalin-2-yl)-1H-indole-6-carboxylate* (**3n**):
Isolated through filtration to produce yellow solid. Yield: 66%. ^1^H NMR (400 MHz, DMSO-*d*_6_) δ 12.10 (s, 1H), 9.09 (s, 1H), 8.94 (d, *J* = 8.4 Hz, 1H), 8.15 (s, 1H), 7.94 (d, *J* = 7.9 Hz, 1H), 7.83 (d, *J* = 8.5 Hz, 1H), 7.56 (s, 2H), 7.41 (d, *J* = 5.0 Hz, 1H), 3.89 (s, 3H), 3.74 (s, 3H). ^13^C NMR (101 MHz, DMSO-*d*_6_) δ 166.97, 153.55, 150.16, 135.99, 135.65, 132.76, 131.60, 129.88, 128.59, 128.52, 123.43, 122.68, 121.46, 114.43, 113.64, 111.67, 51.93, 29.08. This is a known structure. These data are similar to the reported ones [18].
*3-(4-methyl-3-oxo-3,4-dihydroquinoxalin-2-yl)-1H-indole-5-carbonitrile* (**3o**):
Isolated through filtration to produce yellow solid. M.P.: >300 °C. Yield: 37%. ^1^H NMR (400 MHz, DMSO-*d*_6_) δ 12.20 (s, 1H), 9.15 (s, 1H), 8.98 (s, 1H), 7.92 (d, *J* = 7.9 Hz, 1H), 7.64 (d, *J* = 8.4 Hz, 1H), 7.61–7.44 (m, 3H), 7.37 (t, *J* = 7.4 Hz, 1H), 3.66 (s, 3H). ^13^C NMR (101 MHz, DMSO-*d*_6_) δ 153.44, 149.85, 138.14, 135.00, 132.62, 131.64, 128.91, 128.76, 127.98, 125.95, 125.39, 123.52, 120.73, 114.48, 113.31, 111.77, 103.06, 29.11. HRMS (ESI): *m/z* calcd for C_18_H_12_N_4_O [M + Na]^+^: 323.0909. Found: 323.0909.
*3-(1H-indol-3-yl)-1-methylquinoxalin-2(1H)-one* (**3p**):
Isolated through filtration to produce yellow solid. Yield: 86%. ^1^H NMR (400 MHz, DMSO-*d*_6_) δ 12.41 (s, 1H), 11.78 (s, 1H), 8.94 (s, 1H), 8.91–8.85 (m, 1H), 7.86 (d, *J* = 7.9 Hz, 1H), 7.54–7.47 (m, 1H), 7.43 (t, *J* = 7.6 Hz, 1H), 7.32 (d, *J* = 7.8 Hz, 2H), 7.27–7.20 (m, 2H). ^13^C NMR (101 MHz, DMSO-*d*_6_) δ 154.41, 151.98, 136.28, 133.09, 132.65, 130.18, 127.99, 127.61, 126.20, 123.24, 122.99, 122.56, 121.00, 114.94, 111.90, 111.32. This is a known structure. These data are similar to the reported ones [17].
*1-ethyl-3-(1H-indol-3-yl)quinoxalin-2(1H)-one* (**3q**):
Isolated through silica gel column chromatography with ethyl acetate and petrol ether (1:4) to produce red solid. M.P.: 175.0–176.5 °C. Yield: 91%. ^1^H NMR (400 MHz, DMSO-*d*_6_) δ 11.80 (s, 1H), 8.96–8.87 (m, 2H), 7.93 (d, *J* = 7.9 Hz, 1H), 7.63–7.49 (m, 3H), 7.38 (t, *J* = 7.5 Hz, 1H), 7.27–7.21 (m, 2H), 4.38 (q, *J* = 6.7, 2H), 1.30 (t, *J* = 6.9 Hz, 3H). ^13^C NMR (101 MHz, DMSO-*d*_6_) δ 153.17, 150.65, 136.29, 133.26, 133.21, 130.24, 128.73, 128.40, 126.33, 123.36, 123.01, 122.54, 121.00, 114.13, 111.89, 111.34, 36.89, 12.45. HRMS (ESI): *m/z* calcd for C_18_H_15_N_3_O [M + H]^+^: 290.1293. Found: 290.1295.
*1-benzyl-3-(1H-indol-3-yl)quinoxalin-2(1H)-one* (**3r**):
Isolated through silica gel column chromatography with ethyl acetate and petrol ether (1:4) to produce orange solid. Yield: 68%. ^1^H NMR (400 MHz, DMSO-*d*_6_) δ 11.85 (s, 1H), 8.98–8.90 (m, 2H), 7.95 (d, *J* = 7.8 Hz, 1H), 7.56–7.50 (m, 1H), 7.43 (d, *J* = 3.2 Hz, 2H), 7.38–7.29 (m, 5H), 7.29–7.23 (m, 3H), 5.63 (s, 2H). ^13^C NMR (101 MHz, DMSO-*d*_6_) δ 153.84, 150.77, 136.33, 136.19, 133.39, 133.29, 130.61, 128.73, 128.64, 128.27, 127.26, 126.76, 126.33, 123.62, 123.02, 122.62, 121.10, 114.80, 111.94, 111.40, 44.98. This is a known structure. These data are similar to the reported ones [17].
*1-allyl-3-(1H-indol-3-yl)quinoxalin-2(1H)-one* (**3s**):
Isolated through silica gel column chromatography with ethyl acetate and petrol ether (1:4) to produce orange solid. Yield: 72%. ^1^H NMR (400 MHz, DMSO-*d*_6_) δ 11.82 (s, 1H), 8.95–8.87 (m, 2H), 7.94 (d, *J* = 7.9 Hz, 1H), 7.55–7.44 (m, 3H), 7.38 (m, 1H), 7.28–7.21 (m, 2H), 6.08–5.94 (m, 1H), 5.20 (d, *J* = 10.5 Hz, 1H), 5.10 (d, *J* = 16.9 Hz, 1H), 5.01 (d, *J* = 2.4 Hz, 2H). ^13^C NMR (101 MHz, DMSO-*d*_6_) δ 153.41, 150.75, 136.40, 133.36, 133.26, 131.94, 130.63, 128.69, 128.42, 126.39, 123.69, 123.10, 122.75, 121.21, 117.06, 114.90, 112.05, 111.45, 44.05. This is a known structure. These data are similar to the reported ones [17].
*3-(1H-indol-3-yl)-1-(prop-2-yn-1-yl)quinoxalin-2(1H)-one* (**3t**):
Isolated through silica gel column chromatography with ethyl acetate and petrol ether (1:4) to produce yellow solid. Yield: 73%. ^1^H NMR (400 MHz, DMSO-*d*_6_) δ 11.85 (s, 1H), 8.93–8.85 (m, 2H), 7.95 (d, *J* = 7.7 Hz, 1H), 7.60 (t, *J* = 9.0 Hz, 2H), 7.52 (d, *J* = 5.2 Hz, 1H), 7.43 (t, *J* = 7.5 Hz, 1H), 7.28–7.22 (m, 2H), 5.21 (s, 2H), 3.35 (s, 1H). ^13^C NMR (101 MHz, DMSO-*d*_6_) δ 152.78, 150.47, 136.32, 133.34, 133.19, 129.84, 128.60, 128.37, 126.22, 123.92, 122.97, 122.67, 121.14, 114.69, 111.96, 111.20, 78.37, 75.07, 31.34. This is a known structure. These data are similar to the reported ones [17].

### 3.5. Gram Scale Reaction

A 100 mL round bottle equipped with a magnetic stir bar was charged with **1** (5.0 mmol) and **2** (10.0 mmol, 2.0 equiv). FeCl_3_ solution (0.01 mmol/mL in CH_3_CN, 50 mL) and di-tert-butyl peroxide (DTBP, 10.0 mmol, 2.0 equiv) were added. Then, the reaction mixture was stirred at 40 °C for 24 h to produce the suspension. The resulting mixture was analyzed by HPLC. The mixture was filtered to produce pure yellow product **3p** in a 94% yield.

## 4. Patents

The application of a Chinese patent related to the structure and bioactivity of compound **3e** is pending.

## Figures and Tables

**Table 1:** Optimization of reaction conditions *^a^*.

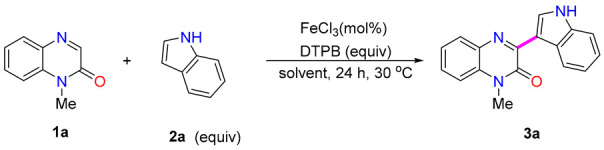					
Entry	2a (equiv)	FeCl_3_ (mol%)	DTPB (equiv)	Solvent	Yield (%) *^b^*
1	1.5	10	2.0	CH_3_CN	81%
2	1.0	10	2.0	CH_3_CN	74%
3	2.0	10	2.0	CH_3_CN	88%
4	3.0	10	2.0	CH_3_CN	86%
5	2.0	1	2.0	CH_3_CN	17%
6	2.0	5	2.0	CH_3_CN	82%
7	2.0	15	2.0	CH_3_CN	81%
8	2.0	10	1.5	CH_3_CN	83%
9	2.0	10	2.5	CH_3_CN	86%
10	2.0	10	2.0	DMF	12%
11	2.0	10	2.0	DCM	52%
12 *^c^*	2.0	10	2.0	CH_3_CN	74%

*^a^* All reactions were conducted at 0.1 mmol scale of **1a** in 1.0 mL of CH_3_CN in a closed flask at 30 °C for 24 h. *^b^* Yield determined by HPLC with biphenyl as the internal standard. *^c^* Reaction conducted for 12 h.

## Data Availability

Data are contained within the article and Supplementary Materials.

## Supplementary Materials

The following supporting information can be downloaded at: https://www.mdpi.com/article/10.3390/molecules29112649/s1, The supporting information include control experiment, characterization data of products, and ^1^H NMR and ^13^C NMR spectra of the products.
