# Supplementary material for: Mild Iron-Catalyzed Oxidative Cross-Coupling of Quinoxalinones with Indoles
Source: Molecules. 2024 Jun 4;29(11):2649. doi: 10.3390/molecules29112649 (PMC11173961; doi:10.3390/molecules29112649)
Supplement: Supplementary file 1 [file molecules-29-02649-s001.zip › molecules-3035880-supplementary.pdf]

Mild Iron-Catalyzed Oxidative Cross-Coupling of Quinoxalinones with Indoles

Hangcheng Ni [1,*], Hui Mao [1,2], Ying Huang [1], Yi Lu [1] and Zhenxiang Liu[1,*]

1 College of Pharmacy, Jinhua Polytechnic, Jinhua 321007, China

2 Key Laboratory of the Ministry of Education for Advanced Catalysis Materials, Zhejiang Normal University, Jinhua 321004, China

* Correspondence: nihc@hotmail.com (H.N.); liuzhenxiang86@163.com (Z.L.)

1. General Information……………..…………………………..…..S2
2. General procedure for the synthesis of starting materials…………......S2
3. General procedure for the synthesis of product…………..................S4
4. Gram scale reaction……….…………….…………….................S9
5. Control experiment………..……..……………………………S9
6. References……………………..S11
7. NMR spectra…………………...S12

1. General Information

^1H NMR and ^{13}C NMR spectra were recorded on a Bruker AVANCE NEO 400MHZ. Spectra were calibrated relative to resonances of the deuterated solvents for proton and carbon chemical shifts: DMSO (δ = 2.50 for ^1H NMR and δ= 39.50 for ^{13}C NMR), $CDCl_3$ (δ = 7.26 for ^1H NMR and δ = 77.16 for ^{13}C NMR). Data are reported as follows: chemical shift δ/ppm, integration (1H only), multiplicity (s = singlet, d = doublet, t =triplet, dd = doublet of doublets, m = multiplet; ^{13}C signals are singlets unless otherwise stated), coupling constants *J* in Hz. HRMS spectra were obtained on a waters G2-XS Qtof. High performance liquid Chromatographic (HPLC) was carried out on a Shimadzu LC-20ADxr. Melting point was recorded on Shanghai INESA Physico-Optical Instrument INESA SGM X-4A. Compounds **1a** and **1c-1f** were prepared using literature methods.[1,2] All other chemicals were purchased from Chemical Co. and used as received unless otherwise specified.

2. General procedure for the synthesis of starting materials (1a and 1c-1f).[1,2]

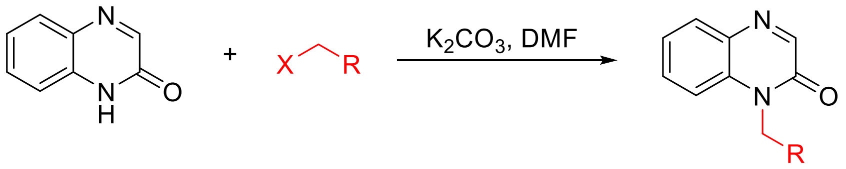

A typical procedure: To a stirred solution of quinoxalin-2(*1H*)-ones (3 mmol) in DMF (10 mL) was added the corresponding halide (1.6 equiv) and potassium carbonate (1.2 equiv.) at room temperature, which was stirred overnight. Then the resulting mixture was added with water (50 mL), and extracted with ethyl acetate (50 mL) for three times. The combined organic layers were dried over Na_2SO_4, filtered and evaporated under reduced pressure. The residue was purified by column chromatography on silica gel to give the desired product **1**.

1-Methylquinoxalin-2(1*H*)-one (1a):

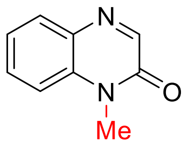

White solid. Yield: 73%. ^1H NMR (600 MHz, DMSO-d_6): δ 8.24 (s, 1H), 7.83 (d, *J* = 8.0 Hz, 1H), 7.67 (t, *J* = 7.5 Hz, 1H), 7.59 (d, *J* = 8.3 Hz, 1H), 7.39 (t, *J* = 7.5 Hz, 1H) (aromatic *H*), 3.61 (s, 3H) (CH_3). This is a known structure. These data are similar to the reported one.[1]

1-Ethylquinoxalin-2(1H)-one (1c):

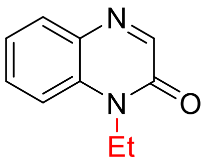

White solid. Yield: 67%. ^1H NMR (600 MHz, DMSO-d_6): δ 8.23 (s, 1H), 7.84 (d, J = 8.4 Hz, 1H), 7.69-7.65 (m, 2H), 7.40-7.38 (m, 1H) (aromatic H), 4.26-4.23 (m, 2H) (CH_2), 1.23 (t, J = 7.2 Hz, 3H) (CH_3). ^{13}C NMR (150 MHz, DMSO-d_6): δ 153.86 (CO), 150.10, 132.91, 131.91, 131.16, 129.87, 123.36, 114.54 (aromatic C), 36.40 (CH_2), 12.29 (CH_3). This is a known structure. These data are similar to the reported one.[1]

1-Benzylquinoxalin-2(1*H*)-one (1d):

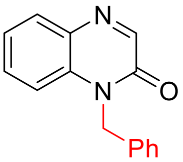

White solid. Yield: 68%. ^1H NMR (600 MHz, DMSO-d_6): δ 8.36 (s, 1H), 7.86 (d, J = 7.9 Hz, 1H), 7.56 (t, J = 7.8 Hz, 1H), 7.46 (d, J = 8.4 Hz, 1H), 7.36 (t, J = 7.6 Hz, 1H), 7.32 (t, J = 7.5 Hz, 2H), 7.28 – 7.24 (m, 3H) (aromatic H), 5.49 (s, 2H) (CH_2). This is a known structure. These data are similar to the reported one.[2]

1-Allylquinoxalin-2(1*H*)-one (1e):

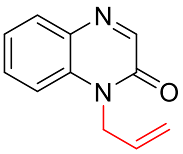

White solid. Yield: 66%. ^1H NMR (600 MHz, DMSO-d_6): δ 8.28 (s, 1H), 7.85 (dd, J = 7.9, 1.2 Hz, 2H), 7.64 (ddd, J = 8.6, 7.2, 1.6 Hz, 1H), 7.51 (dd, J = 8.5, 1.2 Hz, 1H), 7.38 (ddd, J = 8.1, 7.3, 1.2 Hz, 1H) (aromatic H), 5.96 – 5.90 (m, 1H), 5.17 (dd, J = 10.5, 1.5 Hz, 1H), 5.05 (dd, J = 17.3, 1.5 Hz, 1H) (olefinic H), 4.92 – 4.83 (m, 2H) (CH_2). This is a known structure. These data are similar to the reported one.[2]

1-(2-Propynyl)quinoxalin-2(1*H*)-one (1f):

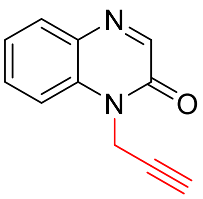

Yellow solid. Yield: 79%. ^1H NMR (600 MHz, DMSO-d_6): δ 8.29 (s, 1H), 7.87 (d, J = 7.9 Hz, 1H), 7.72 (t, J = 7.8 Hz, 1H), 7.65 (d, J = 8.4 Hz, 1H), 7.43 (t, J = 7.6 Hz, 1H) (aromatic H), 5.07 (d, J = 2.5 Hz, 2H) (CH_2), 3.36 (t, J = 2.4 Hz, 1H) (alkynyl H). ^{13}C NMR (151 MHz, DMSO-d_6): δ 153.32 (CO), 150.04, 132.83, 131.45, 131.18, 129.82, 123.91, 115.08 (aromatic C), 77.83, 75.25 (alkynyl C), 30.83 (CH_2). This is a known structure. These data are similar to the reported one.[2]

3. General procedure for the synthesis of product (3a-3t).

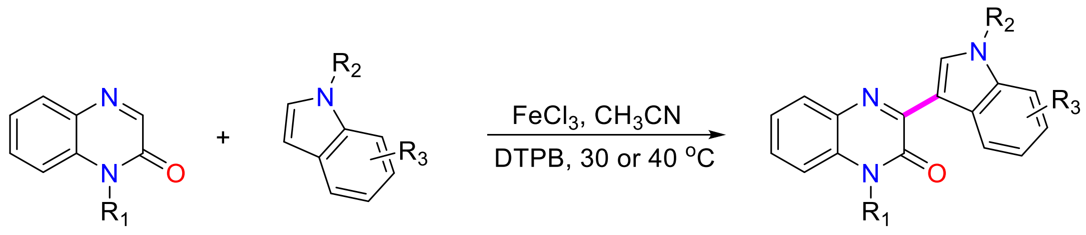

A Schlenk-tube equipped with a magnetic stir bar was charged with **1** (0.5 mmol) and **2** (1.0 mmol, 2.0 equiv). $FeCl_3$ solution (0.01 mmol/mL in CH_3CN, 5 mL) and di-tert-butyl peroxide (DTBP, 1.0 mmol, 2.0 equiv) were added. Then, the reaction mixture was stirred at 30 or 40 °C for 24h. After that, the resulting mixture was analyzed by HPLC, and handled in two ways: (1) For the reaction with precipitant, the mixture was filtered to give the product **3**, which was washed with a small amount of acetonitrile. The solution was further condensed to precipitate the product, and the mixture was filtered; (2) For the reaction without precipitant, the reaction solution was removed under reduced pressure with a rotary evaporator. The crude residue was purified by silica gel column chromatography with ethyl acetate and petrol ether (1:4) as eluent to give the pure product **3**.

3-(*1H*-indol-3-yl)-1-methylquinoxalin-2(*1H*)-one(3a):

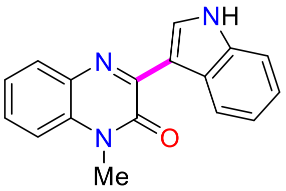

Isolated through filtration to give the yellow solid. Yield: 81%. ^1H NMR (400 MHz, DMSO-d_6) δ 11.79 (s, 1H), 9.03 – 8.85 (m, 2H), 7.92 (d, J = 7.9 Hz, 1H), 7.61 – 7.48 (m, 3H), 7.46 – 7.33 (m, 1H), 7.33 – 7.18 (m, 2H), 3.74 (s, 3H). ^{13}C NMR (101 MHz, DMSO-d_6) δ 153.66, 150.62, 136.29, 133.18, 132.96, 131.49, 128.36, 128.25, 126.31, 123.42, 123.02, 122.53, 120.98, 114.42, 111.88, 111.41, 29.09. This is a known structure. These data are similar to the reported one.[3]

3-(5-iodo-*1H*-indol-3-yl)-1-methylquinoxalin-2(*1H*)-one(3b):

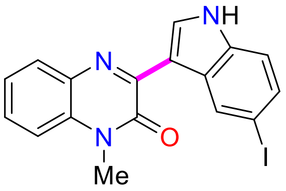

Isolated through filtration to give the yellow solid. M.P.: >300 °C. Yield: 79%. ^1H NMR (400 MHz, DMSO-d_6) δ 11.93 (s, 1H), 9.23 (s, 1H), 8.89 (s, 1H), 7.85 (d, J = 7.9 Hz, 1H), 7.60 – 7.47 (m, 3H), 7.47 – 7.32 (m, 2H), 3.71 (s, 3H). ^{13}C NMR (101 MHz, DMSO-d_6) δ 153.54, 150.22, 135.39, 133.80, 132.72, 131.55, 131.26, 130.51, 128.75, 128.54, 128.34, 123.53, 114.50, 114.36, 110.57, 85.43, 29.10. HRMS (ESI): m/z calcd for $C_{17}H_{12}N_3OI$ $[M+Na]^+$: 423.9923. Found: 423.9923.

3-(5-bromo-*1H*-indol-3-yl)-1-methylquinoxalin-2(*1H*)-one(3c):

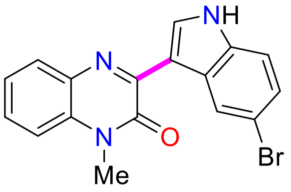

Isolated through filtration to give the yellow solid. Yield: 67%. ^{1}H NMR (400 MHz, DMSO-d_6) δ 11.96 (s, 1H), 9.01 (s, 1H), 8.93 (s, 1H), 7.87 (d, J = 7.9 Hz, 1H), 7.72 – 6.40 (m, 5H), 3.71 (s, 3H). ^{13}C NMR (101 MHz, DMSO-d_6) δ 153.55, 150.23, 135.04, 134.27, 132.73, 131.56, 128.57, 128.40, 127.99, 125.05, 125.00, 123.53, 114.51, 113.94, 113.78, 110.90, 29.11. This is a known structure. These data are similar to the reported one.[3]

3-(5-chloro-*1H*-indol-3-yl)-1-methylquinoxalin-2(*1H*)-one(3d):

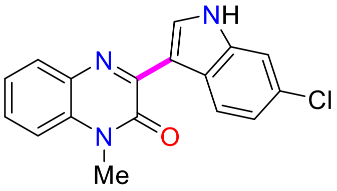

Isolated through filtration to give the yellow solid. Yield: 59%. ^{1}H NMR (400 MHz, DMSO-d_6) δ 11.86 (s, 1H), 8.92 (s, 1H), 8.85 (s, 1H), 7.89 (s, 1H), 7.53 (m, 3H), 7.37 (s, 1H), 7.23 (s, 1H), 3.71 (s, 3H). ^{13}C NMR (101 MHz, DMSO-d_6) δ 153.53, 150.23, 136.80, 133.96, 132.78, 131.58, 128.52, 128.45, 127.09, 125.06, 124.26, 123.43, 121.11, 114.44, 111.58, 111.45, 29.08. This is a known structure. These data are similar to the reported one.[3]

3-(6-fluro-*1H*-indol-3-yl)-1-methylquinoxalin-2(*1H*)-one(3e):

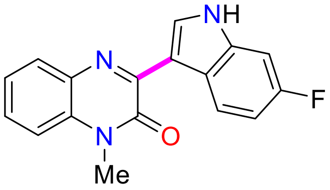

Isolated through filtration to give the yellow solid. M.P.: 261.0-263.0 °C. Yield: 63%. ^{1}H NMR (400 MHz, DMSO-d_6) δ 11.81 (s, 1H), 8.97 – 8.82 (m, 2H), 7.89 (d, J = 7.9 Hz, 1H), 7.52 (t, J = 2.8 Hz, 2H), 7.37 (s, 1H), 7.30 (d, J = 9.7 Hz, 1H), 7.07 (t, J = 9.3 Hz, 1H), 3.71 (d, J = 2.0 Hz, 3H). ^{13}C NMR (101 MHz, DMSO-d_6) δ 159.25 (d, J = 236.4 Hz), 153.55, 150.30, 136.40 (d, J = 12.5 Hz), 133.73 (d, J = 2.6 Hz), 132.82, 131.58, 128.43, 128.42, 124.14 (d, J = 9.6 Hz), 123.42, 123.01, 114.43, 111.45, 109.07 (d, J = 23.4 Hz), 98.06 (d, J = 25.7 Hz), 29.08. HRMS (ESI): m/z calcd for $C_{17}H_{12}N_3OF$ $[M+H]^+$: 294.1043. Found: 294.1046.

3-(5-methoxy-*1H*-indol-3-yl)-1-methylquinoxalin-2(*1H*)-one(3f):

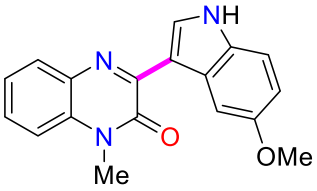

Isolated through filtration to give the yellow solid. Yield: 78%. ^{1}H NMR (400 MHz, DMSO-d_6) δ 11.68 (s, 1H), 8.88 (s, 1H), 8.47 (s, 1H), 7.90 (d, J = 7.9 Hz, 1H), 7.53 (s, 2H), 7.47 – 7.33 (m, 2H), 6.89 (d, J = 8.7 Hz, 1H), 3.88 (s, 3H), 3.72 (s, 3H). ^{13}C NMR (101 MHz, DMSO-d_6) δ 154.87, 153.67, 150.68, 133.52, 132.94, 131.42, 131.17, 128.31, 128.14, 126.99, 123.45, 114.44, 112.48, 112.08, 111.13, 105.06, 55.24, 29.09. This is a known structure. These data are similar to the reported one.[3]

3-(7-methyl-*1H*-indol-3-yl)-1-methylquinoxalin-2(*1H*)-one(3g):

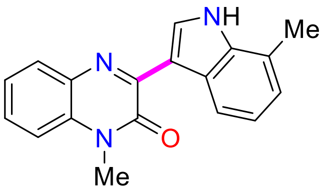

Isolated through filtration to give the yellow solid. M.P.: 289-291 °C. Yield: 83%. ^{1}H NMR (400 MHz, DMSO-d_6)

δ 11.78 (s, 1H), 8.92 (s, 1H), 8.73 (d, *J* = 8.0 Hz, 1H), 7.91 (d, *J* = 7.9 Hz, 1H), 7.53 (d, *J* = 3.9 Hz, 2H), 7.46 – 7.34 (m, 1H), 7.14 (t, *J* = 7.6 Hz, 1H), 7.04 (d, *J* = 7.1 Hz, 1H), 3.73 (s, 3H), 2.53 (s, 3H). ^{13}C NMR (101 MHz, DMSO-d_6) δ 153.70, 150.61, 135.71, 132.96, 132.86, 131.47, 128.36, 128.23, 126.08, 123.41, 123.17, 121.19, 120.93, 120.65, 114.41, 111.81, 29.08, 16.76. HRMS (ESI): *m/z* calcd for $C_{18}H_{15}N_3O$ $[M+H]^+$: 290.1293. Found: 290.1298.

3-(6-methyl-*1H*-indol-3-yl)-1-methylquinoxalin-2(*1H*)-one(3h):

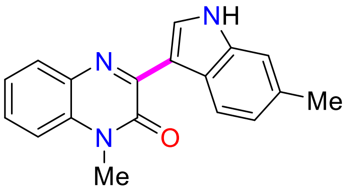

Isolated through filtration to give the yellow solid. Yield: 57%. ^{1}H NMR (400 MHz, DMSO-d_6) δ 11.65 (s, 1H), 8.85 (d, *J* = 2.9 Hz, 1H), 8.74 (d, *J* = 8.1 Hz, 1H), 7.90 (d, *J* = 7.9 Hz, 1H), 7.53 (s, 1H), 7.50 (d, *J* = 9.1 Hz, 1H), 7.42 – 7.34 (m, 1H), 7.30 (s, 1H), 7.06 (d, *J* = 8.2 Hz, 1H), 3.72 (s, 3H), 2.44 (s, 3H). ^{13}C NMR (101 MHz, DMSO-d_6) δ 153.64, 150.60, 136.71, 132.99, 132.73, 131.68, 131.46, 128.32, 128.15, 124.18, 123.39, 122.72, 122.63, 114.40, 111.70, 111.40, 29.07, 21.36. This is a known structure. These data are similar to the reported one.[4]

3-(5-methyl-*1H*-indol-3-yl)-1-methylquinoxalin-2(*1H*)-one(3i):

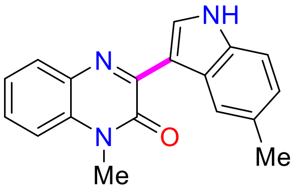

Isolated through filtration to give the yellow solid. M.P.: >300 °C. Yield: 69%. ^{1}H NMR (400 MHz, DMSO-d_6) δ 11.67 (s, 1H), 8.88 (d, *J* = 2.6 Hz, 1H), 8.71 (s, 1H), 7.93 (d, *J* = 7.9 Hz, 1H), 7.54 (d, *J* = 2.2 Hz, 2H), 7.39 (d, *J* = 6.0 Hz, 2H), 7.07 (d, *J* = 8.2 Hz, 1H), 3.73 (s, 3H). ^{13}C NMR (101 MHz, DMSO-d_6) δ 153.67, 150.67, 134.60, 133.22, 132.99, 131.43, 129.63, 128.35, 128.12, 126.57, 123.97, 123.38, 122.80, 114.40, 111.51, 111.02, 29.06, 21.66. HRMS (ESI): *m/z* calcd for $C_{18}H_{15}N_3O$ $[M+H]^+$: 290.1293. Found: 290.1295.

3-(2-methyl-*1H*-indol-3-yl)-1-methylquinoxalin-2(*1H*)-one(3j):

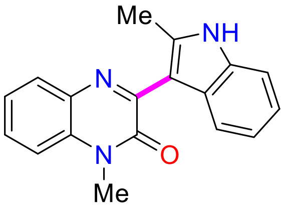

Isolated through silica gel column chromatography with ethyl acetate and petrol ether (1:4) to give the yellow solid. The yellow solid was washed with petrol ether. Yield: 69%. ^{1}H NMR (400 MHz, DMSO-d_6) δ 11.49 (s, 1H), 7.80 (d, *J* = 8.0 Hz, 1H), 7.75 (d, *J* = 7.9 Hz, 1H), 7.62 – 7.52 (m, 2H), 7.41 – 7.31 (m, 2H), 7.07 (t, *J* = 7.8 Hz, 1H), 7.03 – 6.97 (m, 1H), 3.71 (s, 3H), 2.55 (s, 3H). ^{13}C NMR (101 MHz, DMSO-d_6) δ 154.01, 153.14, 139.29, 135.08, 132.81, 132.53, 129.02, 128.65, 127.96, 123.30, 120.83, 120.80, 119.49, 114.42, 110.59, 109.48, 29.28, 14.24. This is a known structure. These data are similar to the reported one.[4]

3-(1-methyl-*1H*-indol-3-yl)-1-methylquinoxalin-2(*1H*)-one(3k):

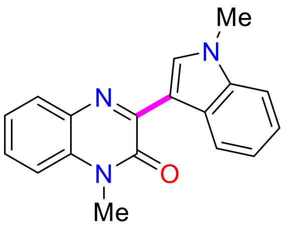

Isolated through filtration to give the yellow solid. Yield: 73%. ^{1}H NMR (400 MHz, DMSO-d_6) δ 8.95 – 8.88 (m, 2H), 7.92 (d, *J* = 7.4 Hz, 1H), 7.56 (d, *J* = 7.2 Hz, 3H), 7.44 – 7.36

(m, 1H), 7.35 – 7.25 (m, 2H), 3.93 (s, 3H), 3.75 (s, 3H). ^{13}C NMR (101 MHz, DMSO-d_6) δ 153.60, 150.32, 136.98, 136.87, 132.97, 131.49, 128.37, 128.31, 126.78, 123.48, 123.14, 122.63, 121.31, 114.48, 110.36, 110.25, 33.07, 29.11. This is a known structure. These data are similar to the reported one.[4]

3-(2-phenyl-*1H*-indol-3-yl)-1-methylquinoxalin-2(*1H*)-one(3l):

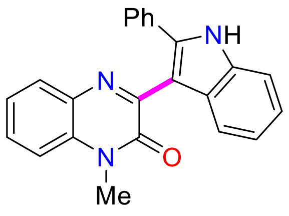

Isolated through silica gel column chromatography with ethyl acetate and petrol ether (1:4) to give the yellow solid. Yield: 57%. ^{1}H NMR (400 MHz, DMSO-d_6) δ 11.84 (s, 1H), 7.78 (d, J = 7.9 Hz, 1H), 7.70 – 7.44 (m, 6H), 7.42 – 7.28 (m, 4H), 7.18 (t, J = 7.2 Hz, 1H), 7.07 (t, J = 7.5 Hz, 1H), 3.59 (s, 3H). ^{13}C NMR (101 MHz, DMSO-d_6) δ 153.72, 153.56, 139.19, 135.96, 133.14, 133.05, 132.71, 129.74, 129.02, 128.57, 128.40, 127.86, 127.81, 123.35, 122.08, 120.33, 119.99, 114.61, 111.45, 109.13, 29.28. This is a known structure. These data are similar to the reported one.[4]

3-(1-phenyl-*1H*-indol-3-yl)-1-methylquinoxalin-2(*1H*)-one(3m):

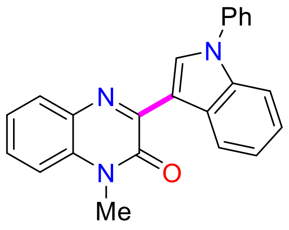

Isolated through filtration to give the yellow solid. Yield: 54%. ^{1}H NMR (400 MHz, DMSO-d_6) δ 9.09 (s, 1H), 9.03 (d, J = 7.4 Hz, 1H), 8.00 (d, J = 7.9 Hz, 1H), 7.73 – 7.57 (m, 7H), 7.56 – 7.48 (m, 1H), 7.42 – 7.31 (m, 2H), 3.76 (s, 3H). ^{13}C NMR (101 MHz, DMSO-d_6) δ 153.68, 144.99, 138.26, 135.79, 134.97, 132.84, 131.72, 130.14, 129.01, 128.76, 127.67, 127.40, 124.52, 123.76, 123.65, 122.25, 114.66, 112.78, 110.74, 29.24. This is a known structure. These data are similar to the reported one.[5]

methyl 3-(4-methyl-3-oxo-3,*4-d*ihydroquinoxalin-2-yl)-*1H*-indole-6-carboxylate (3n):

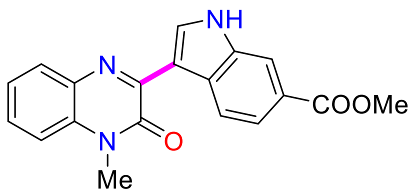

Isolated through filtration to give the yellow solid. Yield: 66%. ^{1}H NMR (400 MHz, DMSO-d_6) δ 12.10 (s, 1H), 9.09 (s, 1H), 8.94 (d, J = 8.4 Hz, 1H), 8.15 (s, 1H), 7.94 (d, J = 7.9 Hz, 1H), 7.83 (d, J = 8.5 Hz, 1H), 7.56 (s, 2H), 7.41 (d, J = 5.0 Hz, 1H), 3.89 (s, 3H), 3.74 (s, 3H). ^{13}C NMR (101 MHz, DMSO-d_6) δ 166.97, 153.55, 150.16, 135.99, 135.65, 132.76, 131.60, 129.88, 128.59, 128.52, 123.43, 122.68, 121.46, 114.43, 113.64, 111.67, 51.93, 29.08. This is a known structure. These data are similar to the reported one.[4]

3-(4-methyl-3-oxo-3,4-dihydroquinoxalin-2-yl)-*1H*-indole-5-carbonitrile(3o):

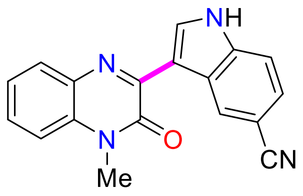

Isolated through filtration to give the yellow solid. M.P.: >300 °C. Yield: 37%. ^{1}H NMR (400 MHz, DMSO-d_6) δ 12.20 (s, 1H), 9.15 (s, 1H), 8.98 (s, 1H), 7.92 (d, J = 7.9 Hz, 1H), 7.64 (d, J = 8.4 Hz, 1H), 7.61 – 7.44 (m, 3H), 7.37 (t, J = 7.4 Hz, 1H), 3.66 (s, 3H). ^{13}C NMR (101 MHz,

DMSO-d_6) δ 153.44, 149.85, 138.14, 135.00, 132.62, 131.64, 128.91, 128.76, 127.98, 125.95, 125.39, 123.52, 120.73, 114.48, 113.31, 111.77, 103.06, 29.11. HRMS (ESI): *m/z* calcd for $C_{18}H_{12}N_4O$ [M+Na]$^+$: 323.0909. Found: 323.0909.

3-(*1H*-indol-3-yl)-1-methylquinoxalin-2(*1H*)-one(3p):

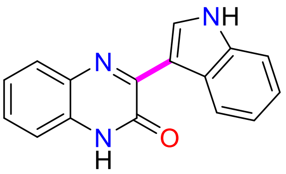

Isolated through filtration to give the yellow solid. Yield: 86%. ^{1}H NMR (400 MHz, DMSO-d_6) δ 12.41 (s, 1H), 11.78 (s, 1H), 8.94 (s, 1H), 8.91 – 8.85 (m, 1H), 7.86 (d, *J* = 7.9 Hz, 1H), 7.54 – 7.47 (m, 1H), 7.43 (t, *J* = 7.6 Hz, 1H), 7.32 (d, *J* = 7.8 Hz, 2H), 7.27 – 7.20 (m, 2H). ^{13}C NMR (101 MHz, DMSO-d_6) δ 154.41, 151.98, 136.28, 133.09, 132.65, 130.18, 127.99, 127.61, 126.20, 123.24, 122.99, 122.56, 121.00, 114.94, 111.90, 111.32. This is a known structure. These data are similar to the reported one.[4]

1-ethyl-3-(*1H*-indol-3-yl)quinoxalin-2(*1H*)-one(3q):

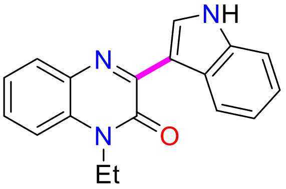

Isolated through silica gel column chromatography with ethyl acetate and petrol ether (1:4) to give the red solid. M.P.: 175-176.5 °C. Yield: 91%. ^{1}H NMR (400 MHz, DMSO-d_6) δ 11.80 (s, 1H), 8.96 – 8.87 (m, 2H), 7.93 (d, *J* = 7.9 Hz, 1H), 7.63 – 7.49 (m, 3H), 7.38 (t, *J* = 7.5 Hz, 1H), 7.27 – 7.21 (m, 2H), 4.38 (q, *J* = 6.7, 2H), 1.30 (t, *J* = 6.9 Hz, 3H). ^{13}C NMR (101 MHz, DMSO-d_6) δ 153.17, 150.65, 136.29, 133.26, 133.21, 130.24, 128.73, 128.40, 126.33, 123.36, 123.01, 122.54, 121.00, 114.13, 111.89, 111.34, 36.89, 12.45. HRMS (ESI): *m/z* calcd for $C_{18}H_{15}N_3O$ [M+H]$^+$: 290.1293. Found: 290.1295.

1-benzyl-3-(*1H*-indol-3-yl)quinoxalin-2(*1H*)-one(3r):

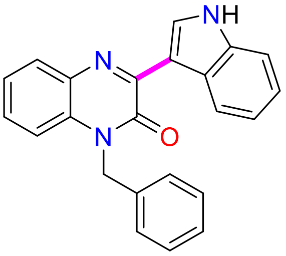

Isolated through silica gel column chromatography with ethyl acetate and petrol ether (1:4) to give the orange solid. Yield: 68%. ^{1}H NMR (400 MHz, DMSO-d_6) δ 11.85 (s, 1H), 8.98 – 8.90 (m, 2H), 7.95 (d, *J* = 7.8 Hz, 1H), 7.56 – 7.50 (m, 1H), 7.43 (d, *J* = 3.2 Hz, 2H), 7.38 – 7.29 (m, 5H), 7.29 – 7.23 (m, 3H), 5.63 (s, 2H). ^{13}C NMR (101 MHz, DMSO-d_6) δ 153.84, 150.77, 136.33, 136.19, 133.39, 133.29, 130.61, 128.73, 128.64, 128.27, 127.26, 126.76, 126.33, 123.62, 123.02, 122.62, 121.10, 114.80, 111.94, 111.40, 44.98. This is a known structure. These data are similar to the reported one.[4]

1-allyl-3-(*1H*-indol-3-yl)quinoxalin-2(*1H*)-one(3s):

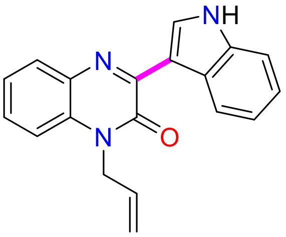

Isolated through silica gel column chromatography with ethyl acetate and petrol ether (1:4) to give the orange solid. Yield: 72%. ^{1}H NMR (400 MHz, DMSO-d_6) δ 11.82 (s, 1H), 8.95 – 8.87 (m, 2H), 7.94 (d, *J* = 7.9 Hz, 1H), 7.55 – 7.44 (m, 3H), 7.38 (m, 1H), 7.28 – 7.21 (m, 2H), 6.08 – 5.94 (m, 1H), 5.20 (d, *J* = 10.5 Hz, 1H), 5.10 (d, *J* = 16.9 Hz, 1H), 5.01 (d, *J* =

2.4 Hz, 2H). ^{13}C NMR (101 MHz, DMSO-d_6) δ 153.41, 150.75, 136.40, 133.36, 133.26, 131.94, 130.63, 128.69, 128.42, 126.39, 123.69, 123.10, 122.75, 121.21, 117.06, 114.90, 112.05, 111.45, 44.05. This is a known structure. These data are similar to the reported one.[4]

3-(*1H*-indol-3-yl)-1-(prop-2-yn-1-yl)quinoxalin-2(*1H*)-one(3t):

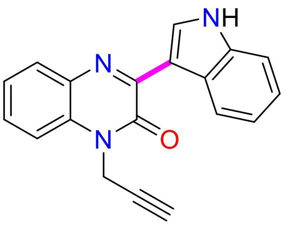

Isolated through silica gel column chromatography with ethyl acetate and petrol ether (1:4) to give the yellow solid. Yield: 73%. ^{1}H NMR (400 MHz, DMSO-d_6) δ 11.85 (s, 1H), 8.93 – 8.85 (m, 2H), 7.95 (d, J = 7.7 Hz, 1H), 7.60 (t, J = 9.0 Hz, 2H), 7.52 (d, J = 5.2 Hz, 1H), 7.43 (t, J = 7.5 Hz, 1H), 7.28 – 7.22 (m, 2H), 5.21 (s, 2H), 3.35 (s, 1H). ^{13}C NMR (101 MHz, DMSO-d_6) δ 152.78, 150.47, 136.32, 133.34, 133.19, 129.84, 128.60, 128.37, 126.22, 123.92, 122.97, 122.67, 121.14, 114.69, 111.96, 111.20, 78.37, 75.07, 31.34. This is a known structure. These data are similar to the reported one.[4]

4. Gram scale reaction.

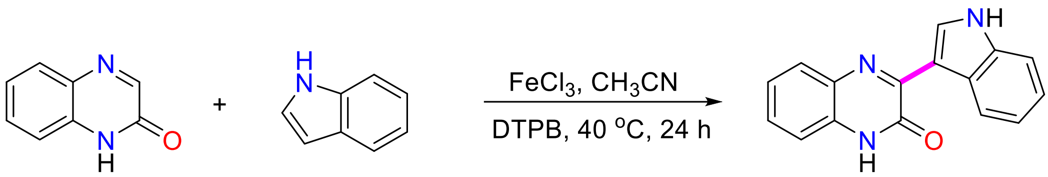

1b (0.73g) **2a** (1.17g) **3p** (1.23g)
Yield: 94%

A 100 mL round-bottle equipped with a magnetic stir bar was charged with **1** (5.0 mmol) and **2** (10.0 mmol, 2.0 equiv). $FeCl_3$ solution (0.01 mmol/mL in CH_3CN, 50 mL) and di-tert-butyl peroxide (DTBP, 10.0 mmol, 2.0 equiv) were added. Then, the reaction mixture was stirred at 40 °C for 24h to give the suspension. The resulting mixture was analyzed by HPLC. The mixture was filtered to give the pure yellow product **3p** in 94% yield.

5. Control experiment

(1) Isolation of intermediate 4a

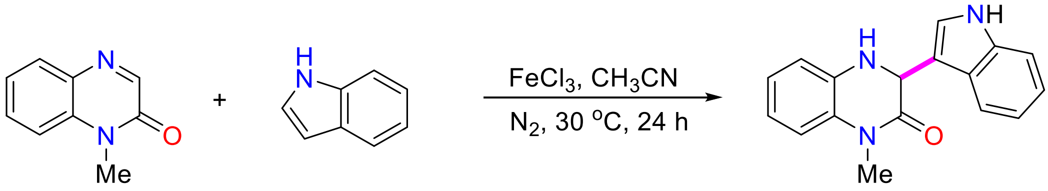

1a **2a** **4a**
Yield: 42%

A 10 mL Schlenk-tube equipped with a magnetic stir bar was charged with **1a** (0.5

mmol) and **2a** (1.0 mmol, 2.0 equiv). $FeCl_3$ solution (0.01 mmol/mL in CH_3CN, 5.0 mL) was added. The mixture was bubbled with nitrogen for 20 min. Then, the reaction mixture was stirred at 30 °C for 24h to give the suspension. The resulting mixture was analyzed by HPLC. The mixture was filtered to give the pale-yellow product **4a** in 42% yield (purity: 87% with 13% **3a**). ^1H NMR (400 MHz, DMSO-d_6) δ 10.93 (s, 1H), 7.63 (d, J = 8.0 Hz, 1H), 7.33 (d, J = 8.1 Hz, 1H), 7.07 (t, J = 7.7 Hz, 1H), 7.03 – 6.92 (m, 3H), 6.90 – 6.80 (m, 2H), 6.74 (t, J = 7.6 Hz, 1H), 6.59 (s, 1H), 5.20 (s, 1H), 3.29 (s, 3H). HRMS (ESI): m/z calcd for $C_{17}H_{15}N_3O$ $[M+Na]^+$: 300.1113. Found: 300.1309.

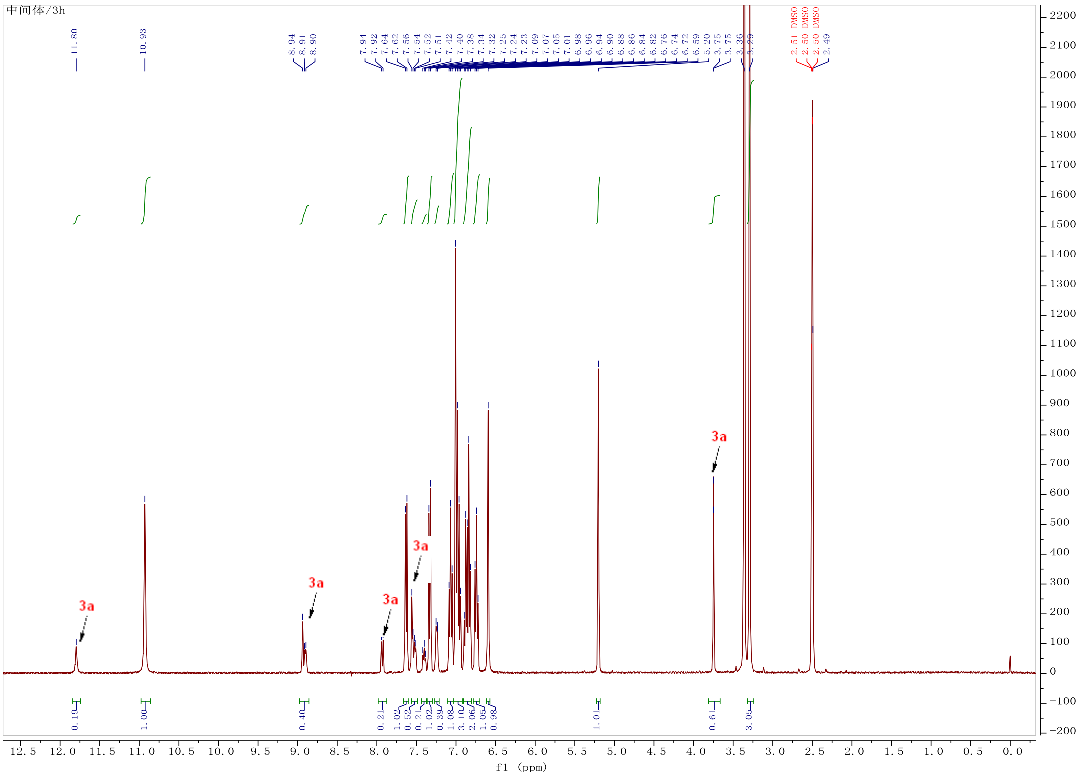

Figure S1. NMR spectra of **4a.**

(2) Conversion of intermediate 4a to 3a

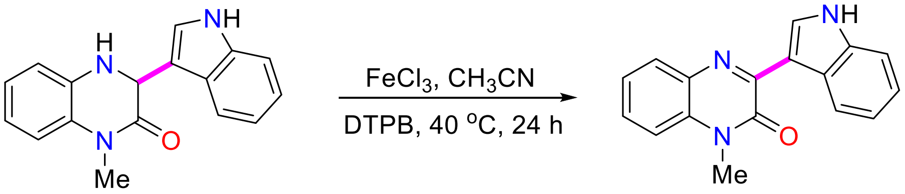

4a

3a
Yield: 63%

A 10 mL Schlenk-tube equipped with a magnetic stir bar was charged with **4a**

(0.1mmol). $FeCl_3$ solution (0.01 mmol/mL in CH_3CN, 1.0 mL) and di-tert-butyl peroxide (DTBP, 0.2 mmol, 2.0 equiv) were added. Then, the reaction mixture was stirred at 40 °C for 24h. The resulting mixture was analyzed by HPLC with biphenyl as the external standard. Yield of product **3a** was determined to be 63%.

6 References:

1 H. Ni, Y. Li, X. Shi, Y. Pang, C. Jin, F. Zhao. *Tetrahedron Lett.*, **2021**, 68, 152915.

2 H. Ni, X. Shi, Y. Li, X. Zhang, J. Zhao, F. Zhao. *Org. Biomol. Chem.*, **2020**, *18*, 6558.

3 Y.-Y. Han, Z.-J. Wu, X.-M. Zhang, W.-C.Yuan, *Tetrahedron Lett.,* **2010**, *51*, 2023.

4 J. Huang, L. Wang, X.-Y. Tang, *Org. Biomol. Chem.*, **2023**, *21*, 2709.

5 M. Shen, L. Li, Q. Zhou, J. Wang, L. Wang, *Chin. J. Org. Chem.*, **2023**, *43*, 697.

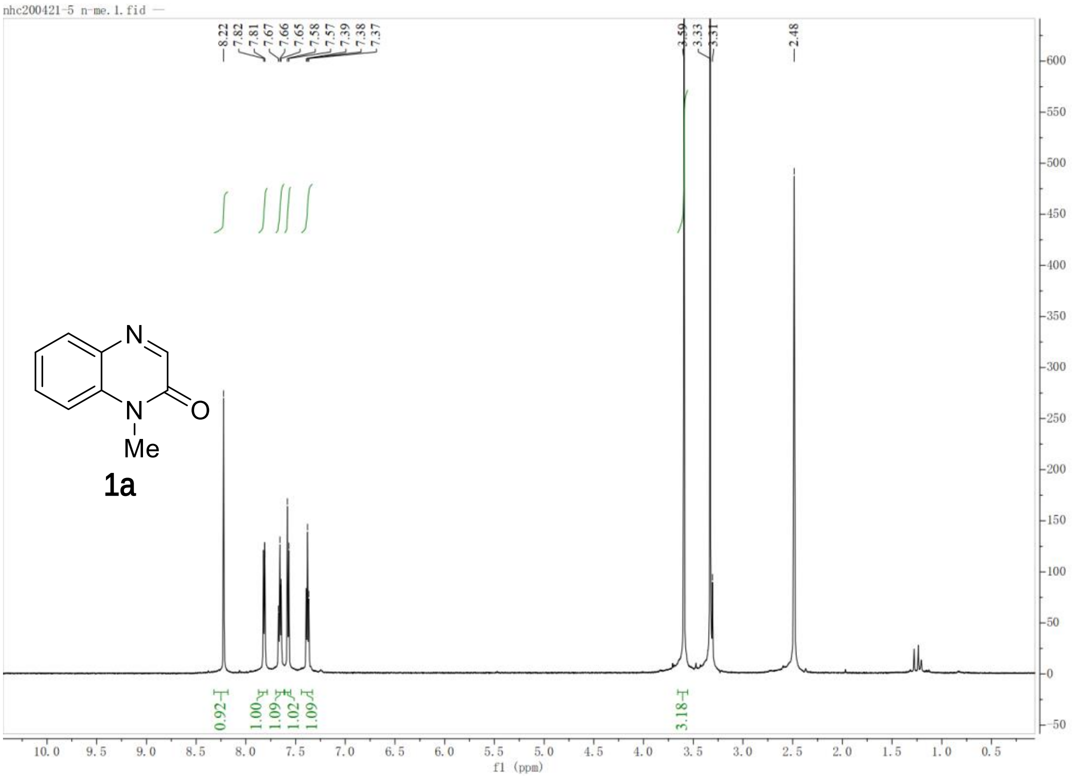
nhc200421-5 n-me.1.fid
8.22
7.82
7.81
7.67
7.66
7.65
7.58
7.57
7.39
7.38
7.37
3.59
3.33
3.31
2.48
0.92
1.00
1.09
1.02
1.09
3.18
1a
f1 (ppm)

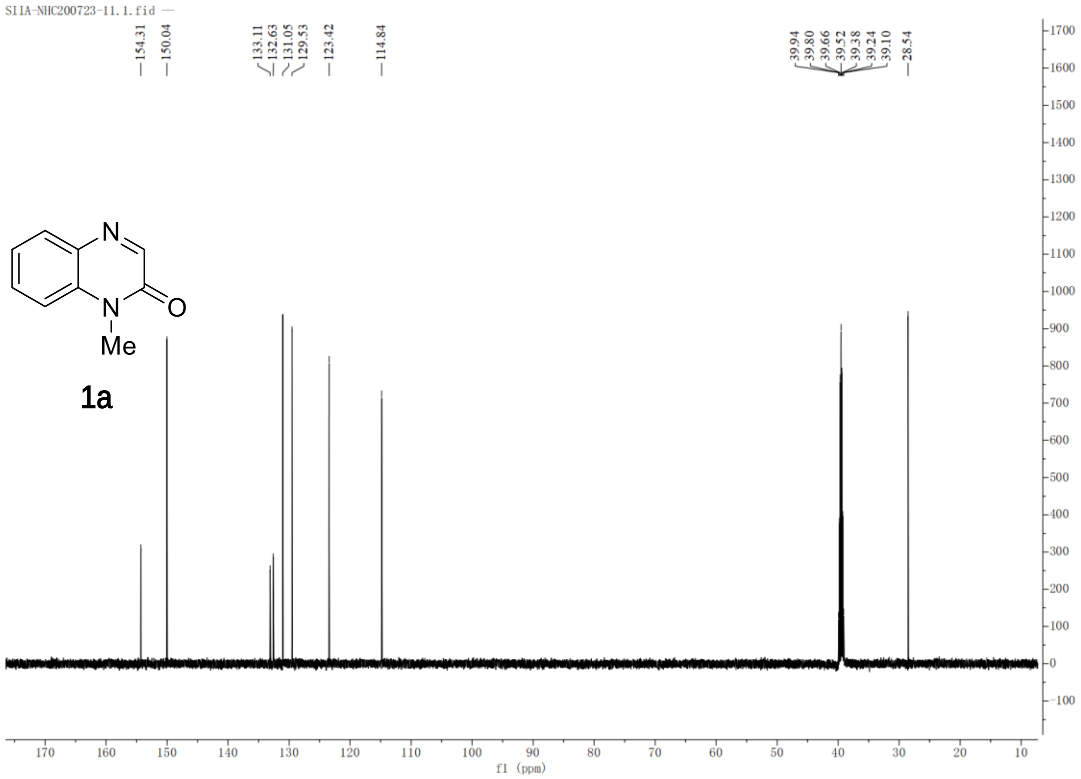
SIIA-NHC200723-11.1.fid
154.31
150.04
133.11
132.63
131.05
129.53
123.42
114.84
39.94
39.80
39.66
39.52
39.38
39.24
39.10
28.54
1a
f1 (ppm)

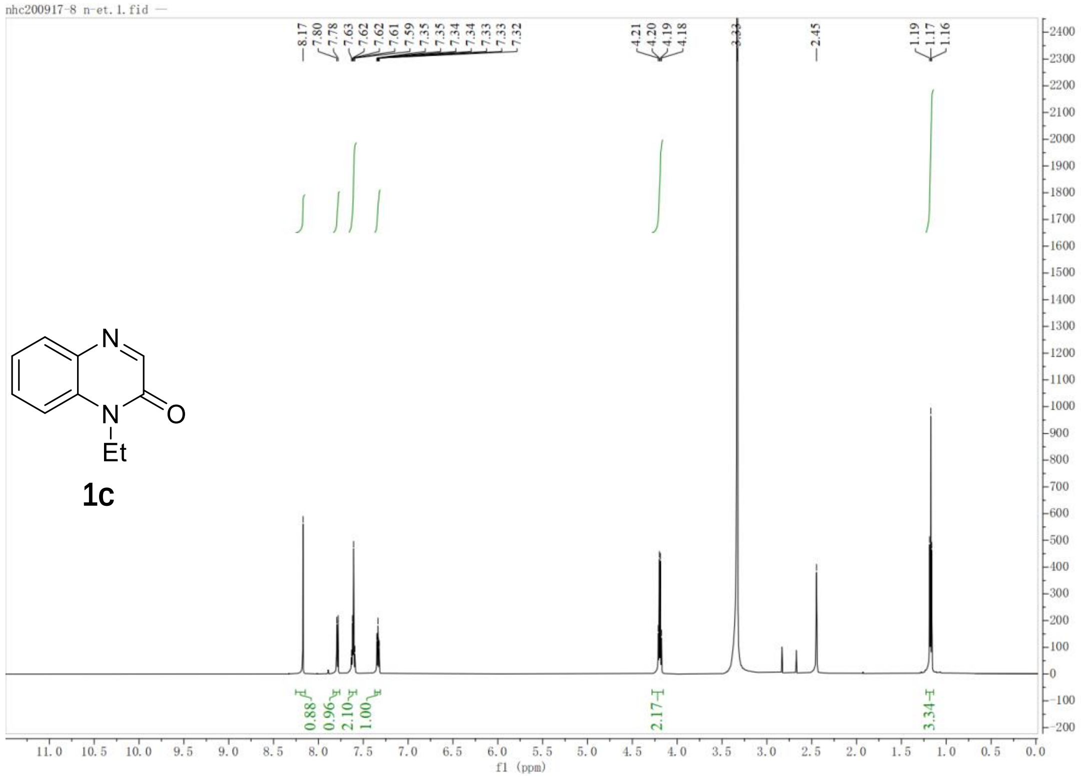
nhc200917-8 n-et.1.fid
8.17
7.80
7.78
7.63
7.62
7.62
7.61
7.59
7.35
7.35
7.34
7.34
7.33
7.33
7.32
4.21
4.20
4.19
4.18
3.33
2.45
1.19
1.17
1.16
0.88
0.96
2.10
1.00
2.17
3.34
f1 (ppm)

1c

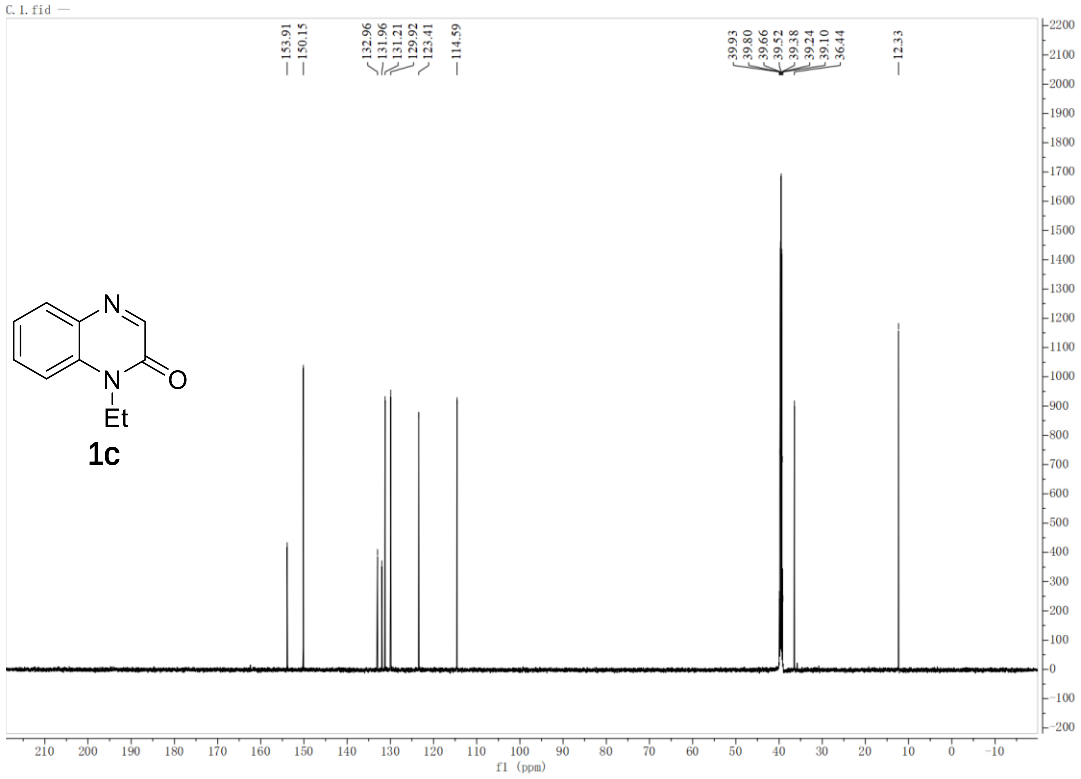
C.1.fid
153.91
150.15
132.96
131.96
131.21
129.92
123.41
114.59
39.93
39.80
39.66
39.52
39.38
39.24
39.10
36.44
12.33
f1 (ppm)

1c

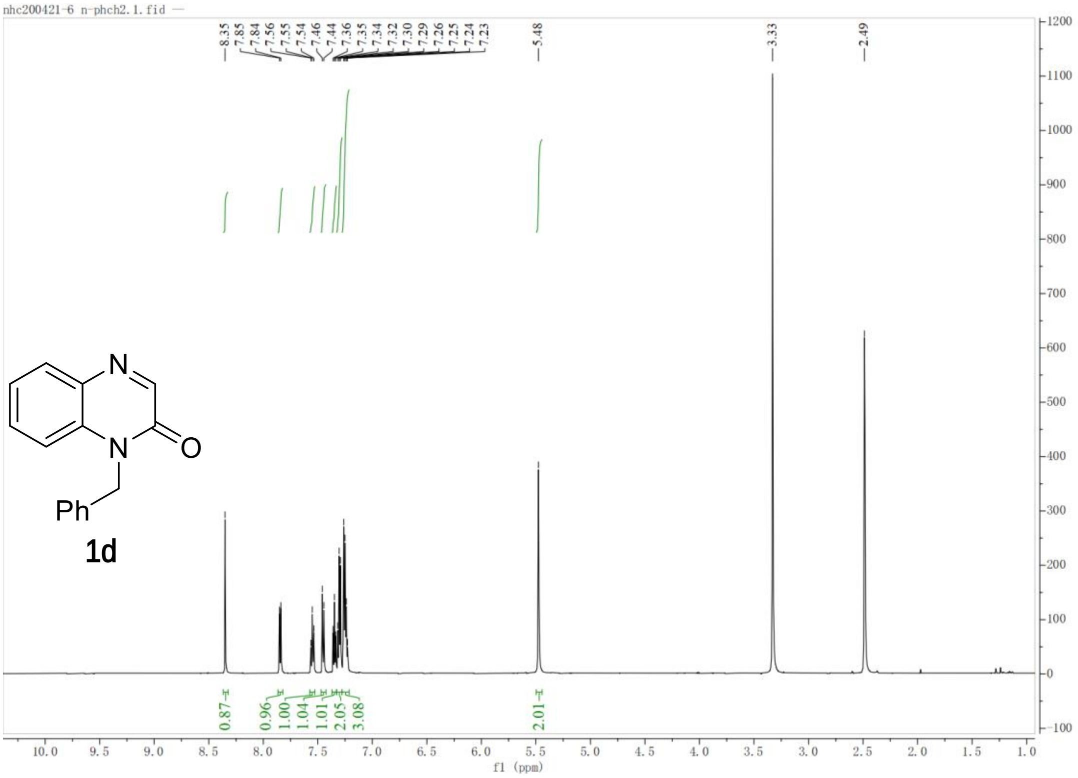
nhc200421-6 n-phch2.1.fid
8.35
7.85
7.84
7.56
7.55
7.54
7.46
7.44
7.36
7.35
7.34
7.32
7.30
7.29
7.26
7.25
7.24
7.23
5.48
3.33
2.49
N
N
O
Ph
0.87
0.96
1.00
1.04
1.01
2.05
3.08
2.01
f1 (ppm)

1d

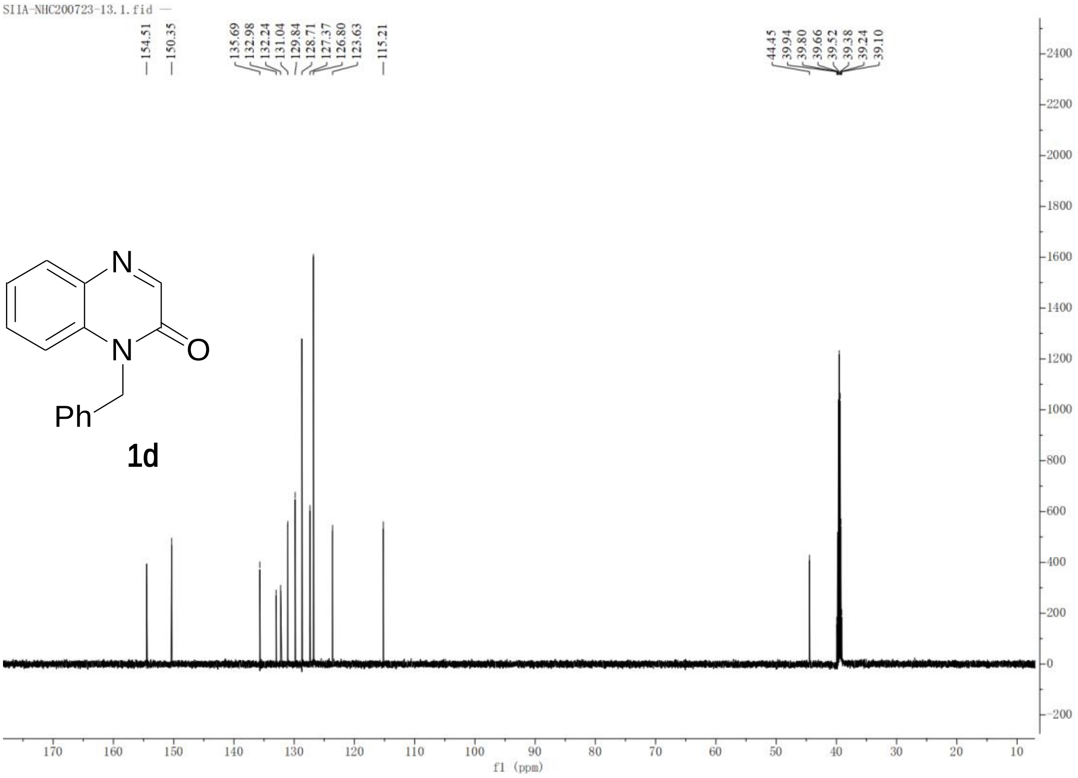
SIIA-NHC200723-13.1.fid
154.51
150.35
135.69
132.98
132.24
131.04
129.84
128.71
127.37
126.80
123.63
115.21
44.45
39.94
39.80
39.66
39.52
39.38
39.24
39.10
N
N
O
Ph
f1 (ppm)

1d

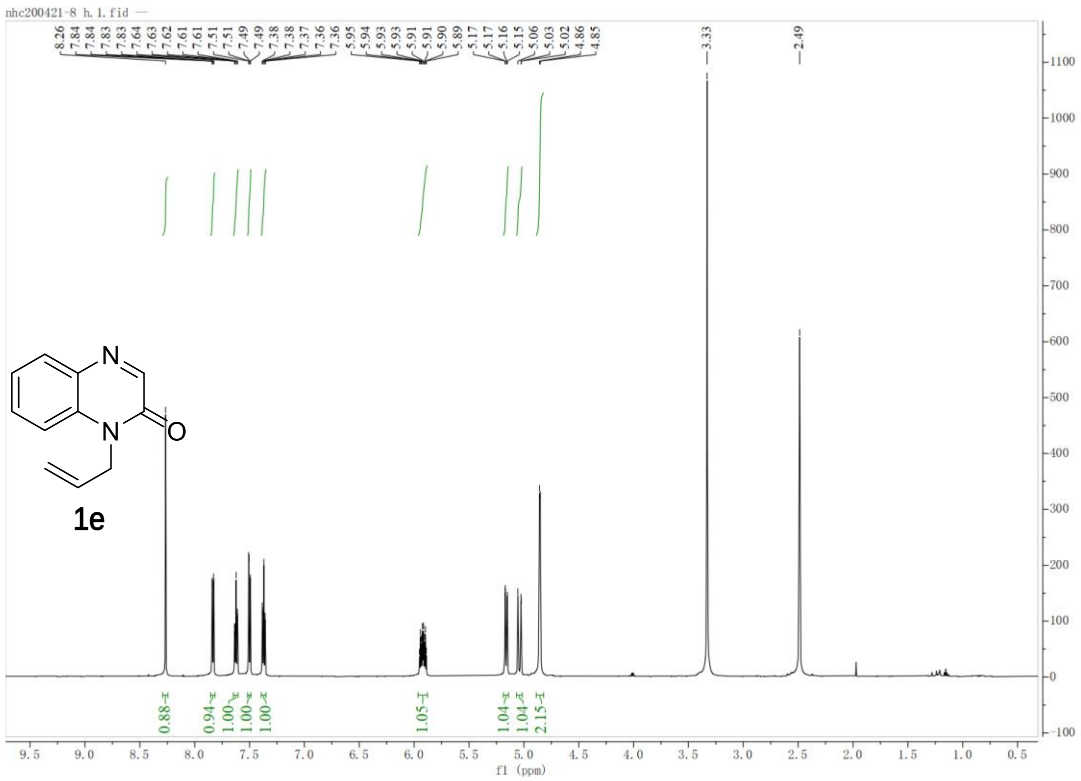
nhc200421-8 h.1.fid
8.26 7.84 7.83 7.83 7.64 7.63 7.62 7.61 7.61 7.51 7.51 7.49 7.49 7.38 7.38 7.37 7.36 7.36 5.95 5.94 5.93 5.93 5.91 5.91 5.90 5.89 5.17 5.17 5.16 5.15 5.06 5.03 5.02 4.86 4.85
3.33
2.49
N
N
O
1e
0.88 0.94 1.00 1.00 1.00 1.05 1.04 1.04 2.15
f1 (ppm)

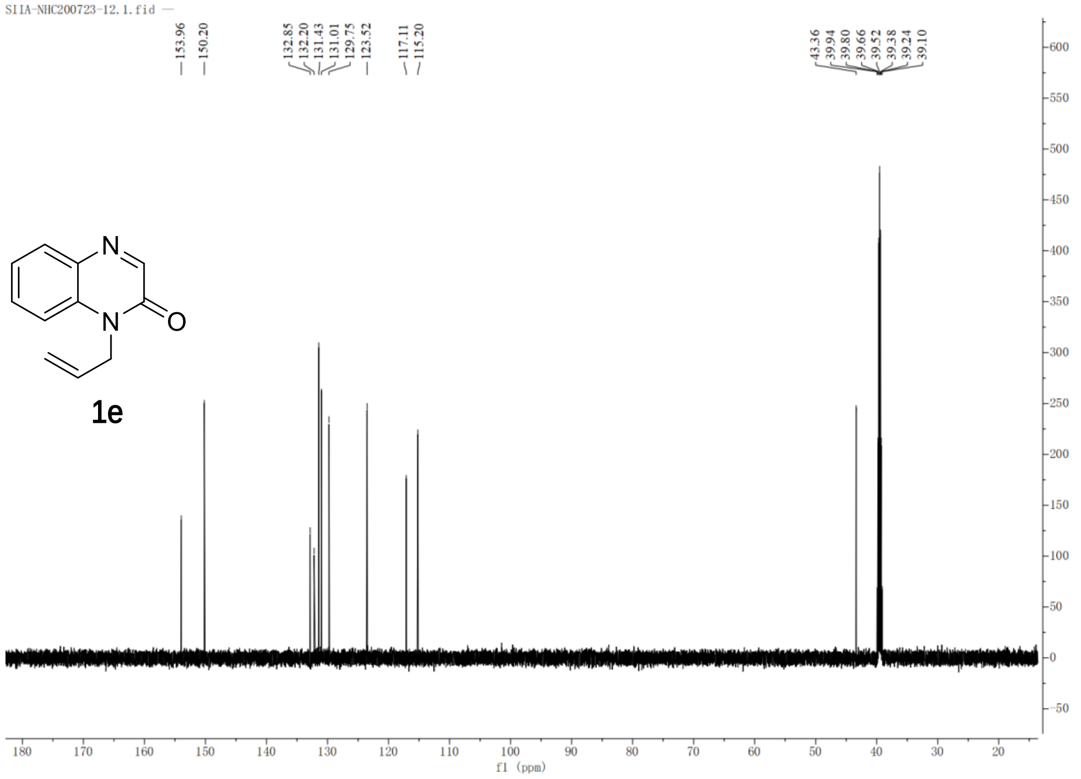
SIIA-NHC200723-12.1.fid
153.96
150.20
132.85 132.20 131.43 131.01 129.75 123.52
117.11 115.20
43.36 39.94 39.80 39.66 39.52 39.38 39.24 39.10
N
N
O
1e
f1 (ppm)

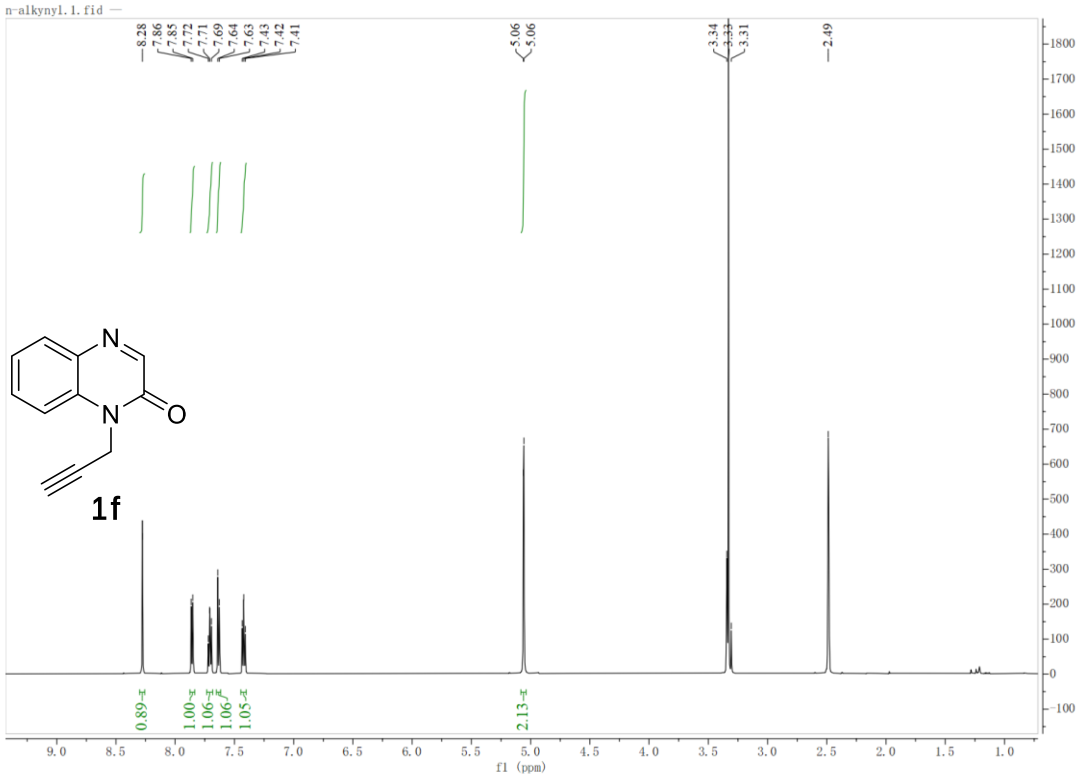
n-alkynyl.1.fid —
8.28
7.86
7.85
7.72
7.71
7.69
7.64
7.63
7.43
7.42
7.41
5.06
5.06
3.34
3.33
3.31
2.49
0.89
1.00
1.06
1.06
1.05
2.13
N
N
O
1f
f1 (ppm)

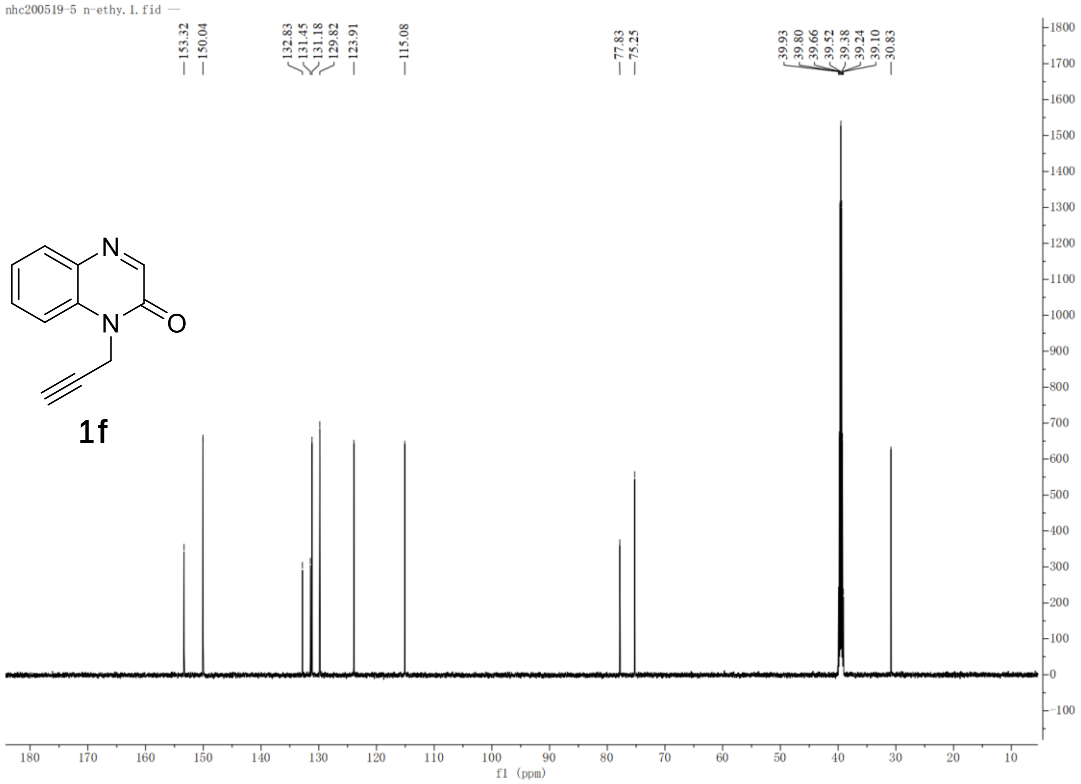
nhc200519-5 n-ethy.1.fid —
153.32
150.04
132.83
131.45
131.18
129.82
123.91
115.08
77.83
75.25
39.93
39.80
39.66
39.52
39.38
39.24
39.10
30.83
N
N
O
1f
f1 (ppm)

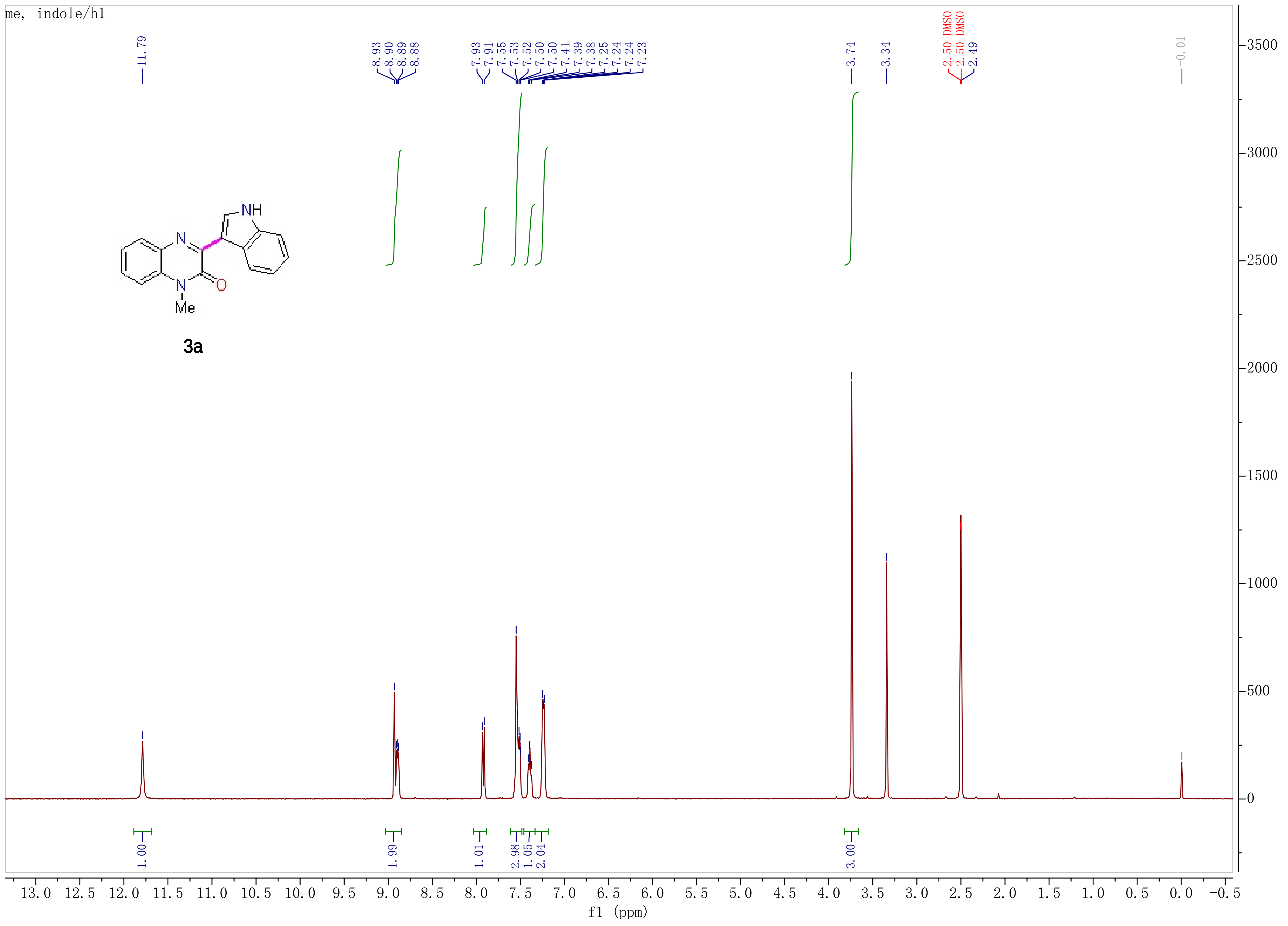
me, indole/h1
11.79
8.93
8.90
8.89
8.88
7.93
7.91
7.55
7.53
7.52
7.50
7.50
7.41
7.39
7.38
7.25
7.24
7.24
7.23
3.74
3.34
2.50 DMSO
2.50 DMSO
2.49
-0.01
NH
N
N
O
Me
3a
1.00
1.99
1.01
2.98
1.05
2.04
3.00
3500
3000
2500
2000
1500
1000
500
0
13.0
12.5
12.0
11.5
11.0
10.5
10.0
9.5
9.0
8.5
8.0
7.5
7.0
6.5
6.0
5.5
5.0
4.5
4.0
3.5
3.0
2.5
2.0
1.5
1.0
0.5
0.0
-0.5
f1 (ppm)

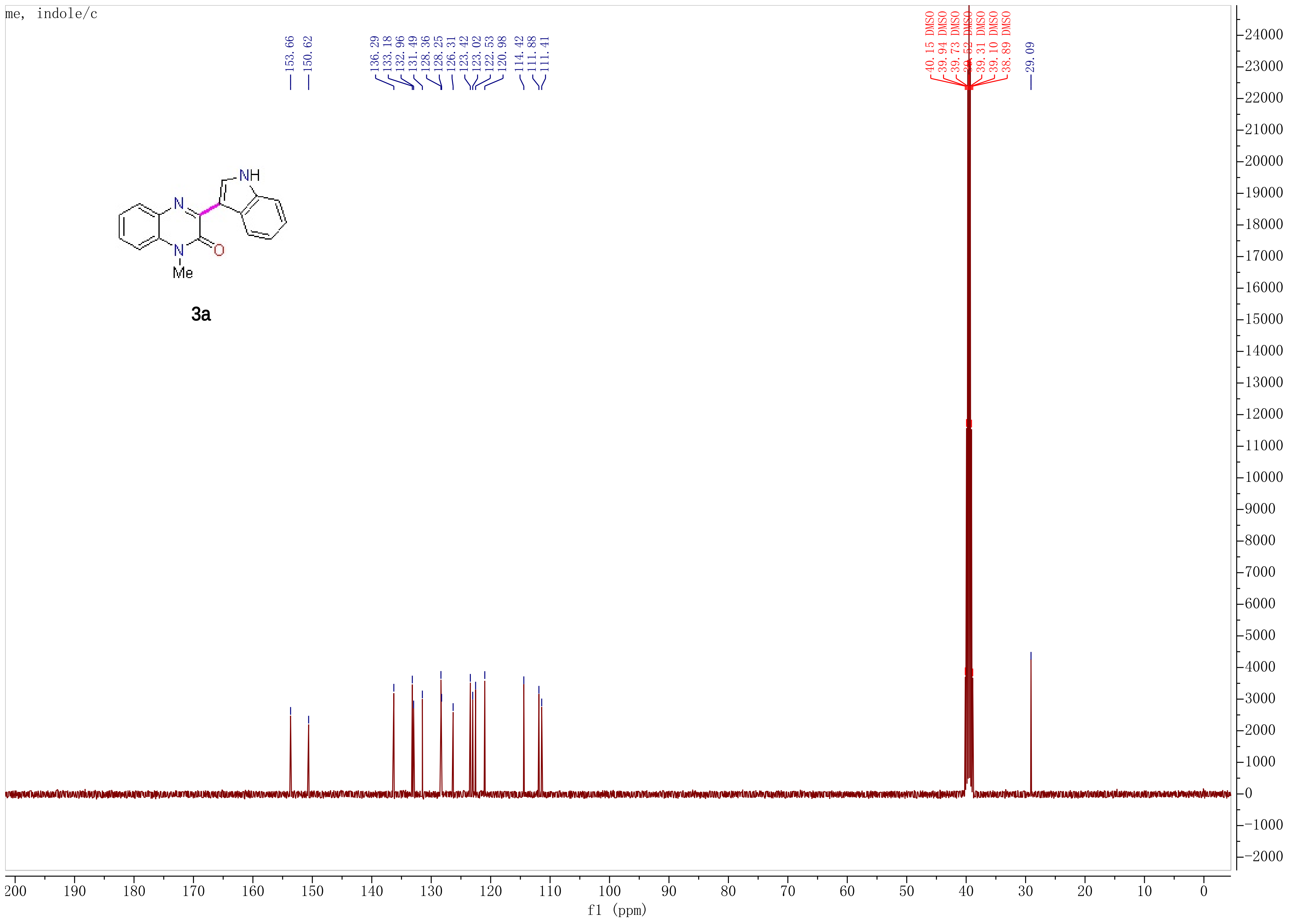

me, indole/c
153.66
150.62
136.29
133.18
132.96
131.49
128.36
128.25
126.31
123.42
123.02
122.53
120.98
114.42
111.88
111.41
40.15 DMSO
39.94 DMSO
39.73 DMSO
39.52 DMSO
39.31 DMSO
39.10 DMSO
38.89 DMSO
29.09
NH
N
N
O
Me
3a
f1 (ppm)

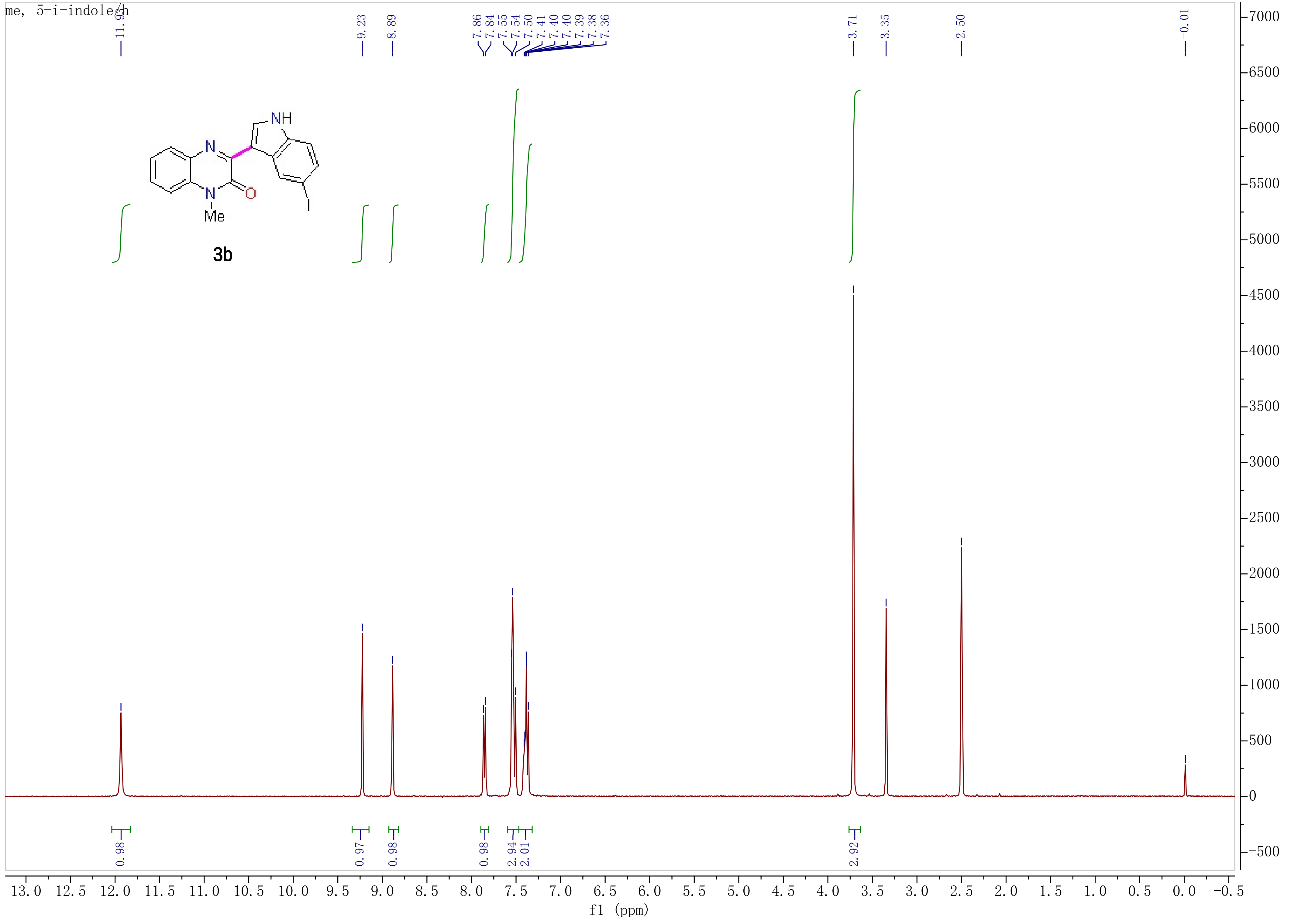
me, 5-i-indole/h
11.93
9.23
8.89
7.86
7.84
7.55
7.54
7.50
7.41
7.40
7.40
7.39
7.38
7.36
3.71
3.35
2.50
-0.01
NH
N
N
O
Me
I
3b
0.98
0.97
0.98
0.98
2.94
2.01
2.92
f1 (ppm)

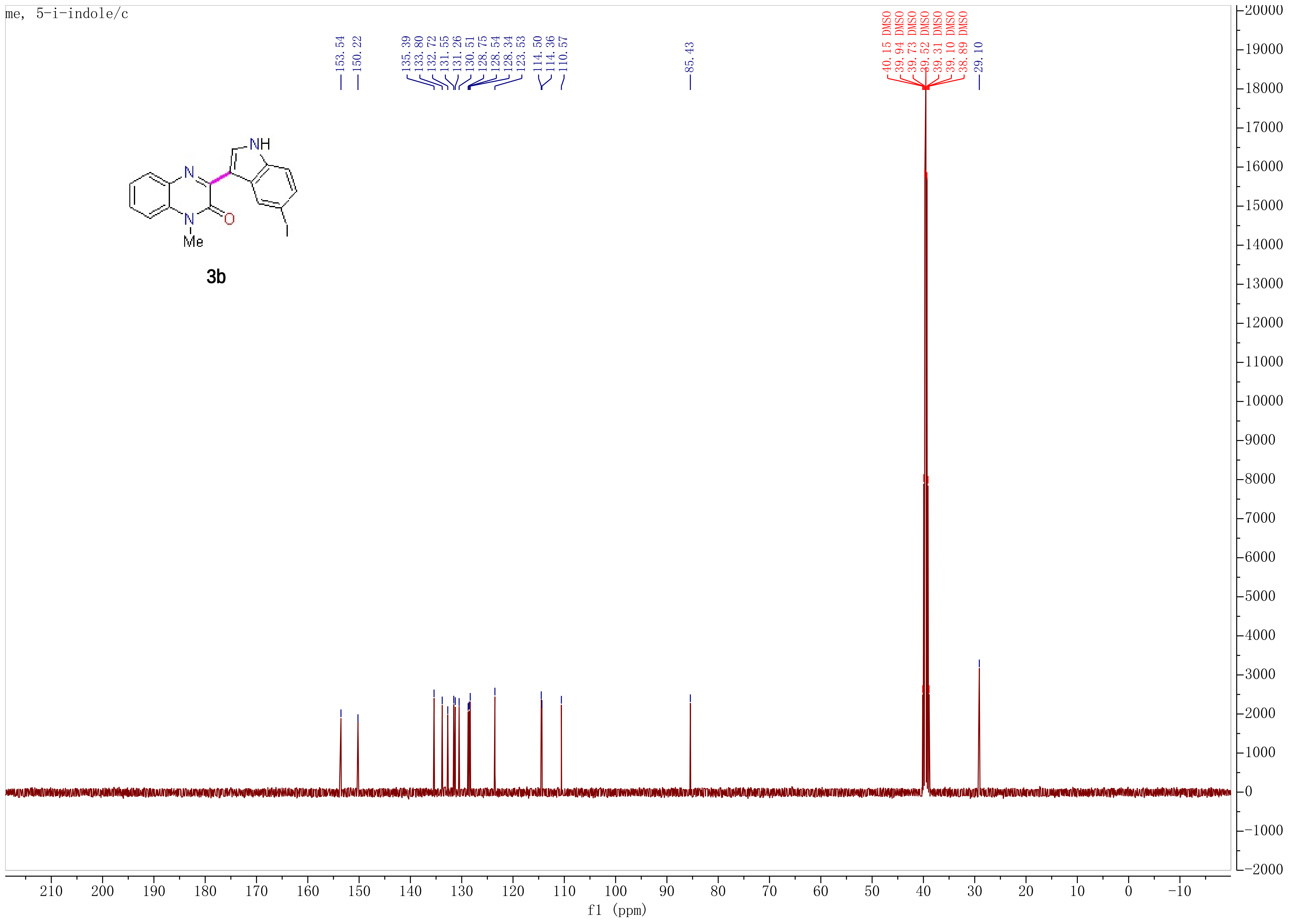
me, 5-i-indole/c
153.54
150.22
135.39
133.80
132.72
131.55
131.26
130.51
128.75
128.54
128.34
123.53
114.50
114.36
110.57
85.43
40.15 DMSO
39.94 DMSO
39.73 DMSO
39.52 DMSO
39.31 DMSO
39.10 DMSO
38.89 DMSO
29.10
NH
N
N
O
Me
I
3b
f1 (ppm)

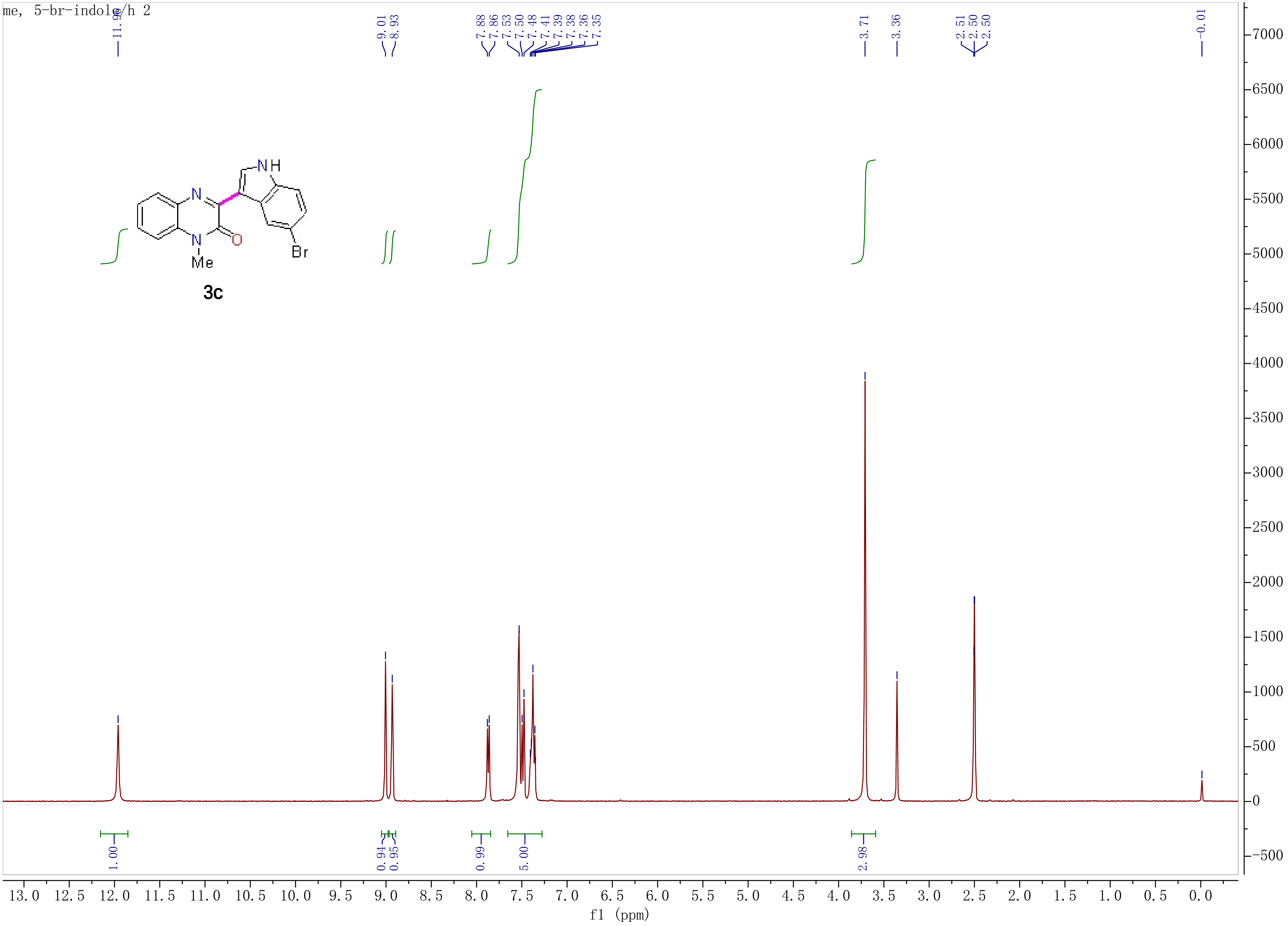
me, 5-br-indole/h 2
9.01
8.93
7.88
7.86
7.53
7.50
7.48
7.41
7.39
7.38
7.36
7.35
3.71
3.36
2.51
2.50
2.50
-0.01
NH
N
N
O
Me
Br
3c
1.00
0.94
0.95
0.99
5.00
2.98
f1 (ppm)

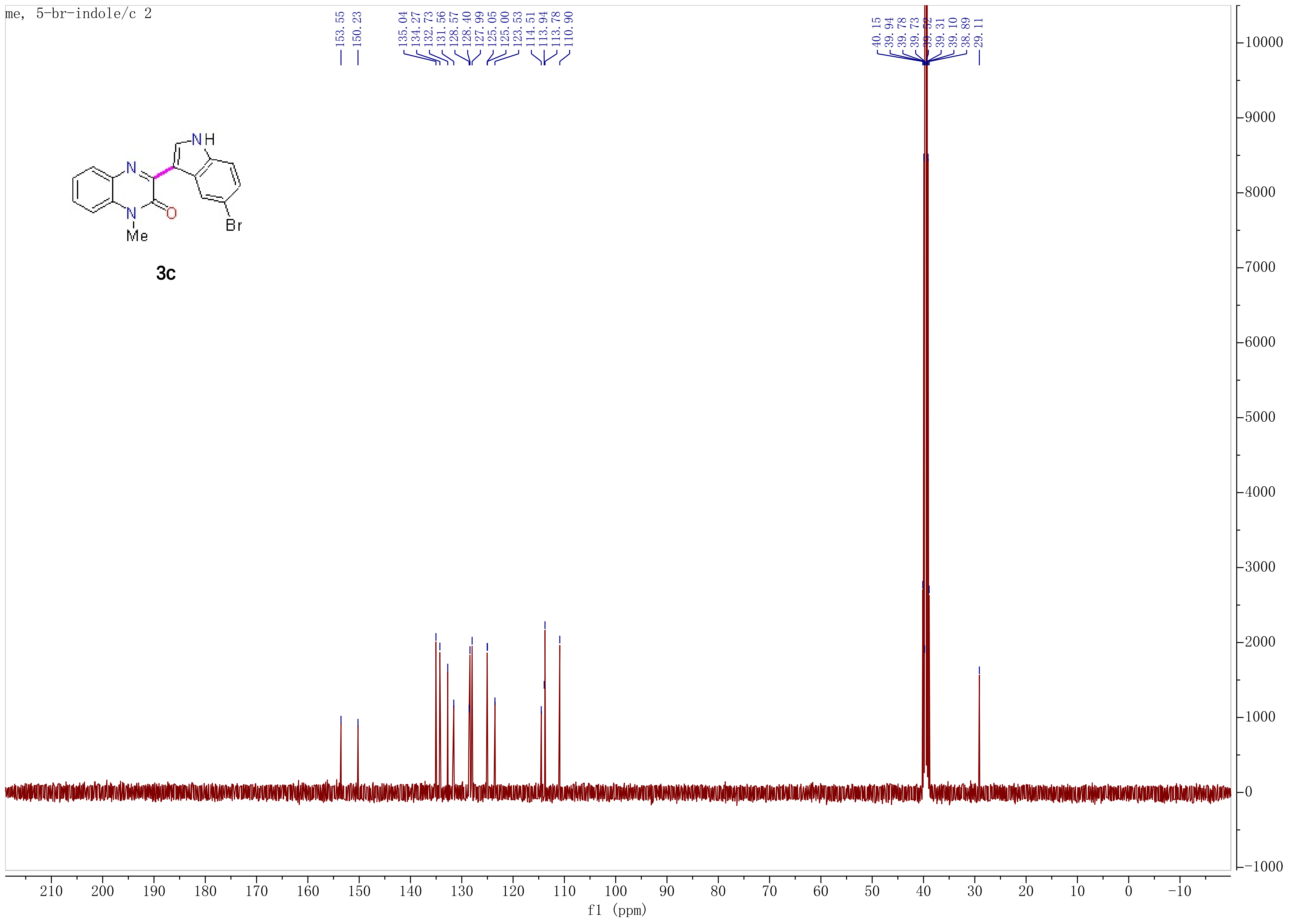
me, 5-br-indole/c 2
153.55
150.23
135.04
134.27
132.73
131.56
128.57
128.40
127.99
125.05
125.00
123.53
114.51
113.94
113.78
110.90
40.15
39.94
39.78
39.73
39.52
39.31
39.10
38.89
29.11
NH
N
N
O
Me
Br
3c
10000
9000
8000
7000
6000
5000
4000
3000
2000
1000
0
−1000
210 200 190 180 170 160 150 140 130 120 110 100 90 80 70 60 50 40 30 20 10 0 −10
f1 (ppm)

me,6-cl/3 h

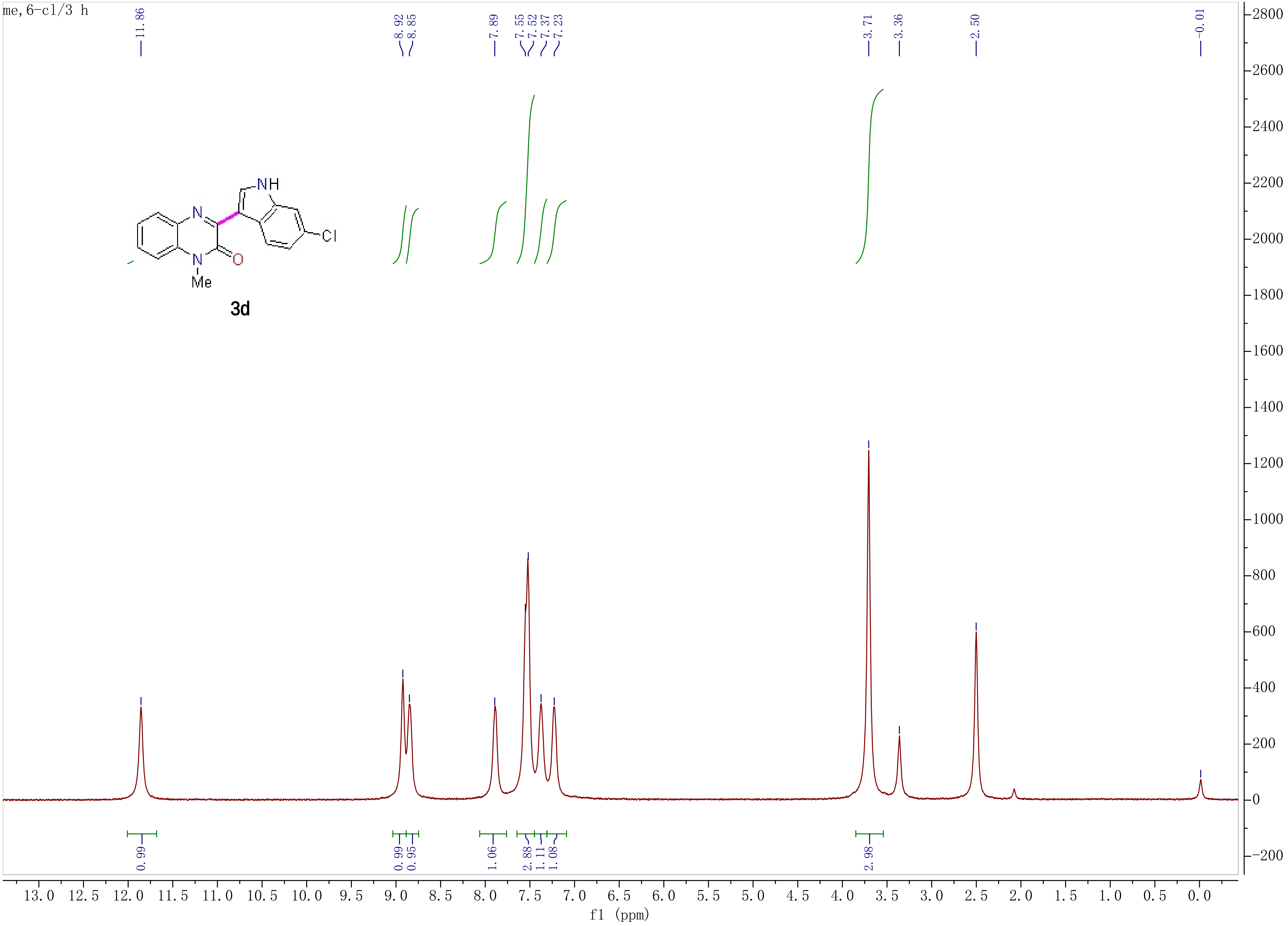
NH
N
N
O
Cl
Me
3d
11.86
8.92
8.85
7.89
7.55
7.52
7.37
7.23
3.71
3.36
2.50
-0.01
0.99
0.99
0.95
1.06
2.88
1.11
1.08
2.98
2800
2600
2400
2200
2000
1800
1600
1400
1200
1000
800
600
400
200
0
-200
13.0 12.5 12.0 11.5 11.0 10.5 10.0 9.5 9.0 8.5 8.0 7.5 7.0 6.5 6.0 5.5 5.0 4.5 4.0 3.5 3.0 2.5 2.0 1.5 1.0 0.5 0.0
f1 (ppm)

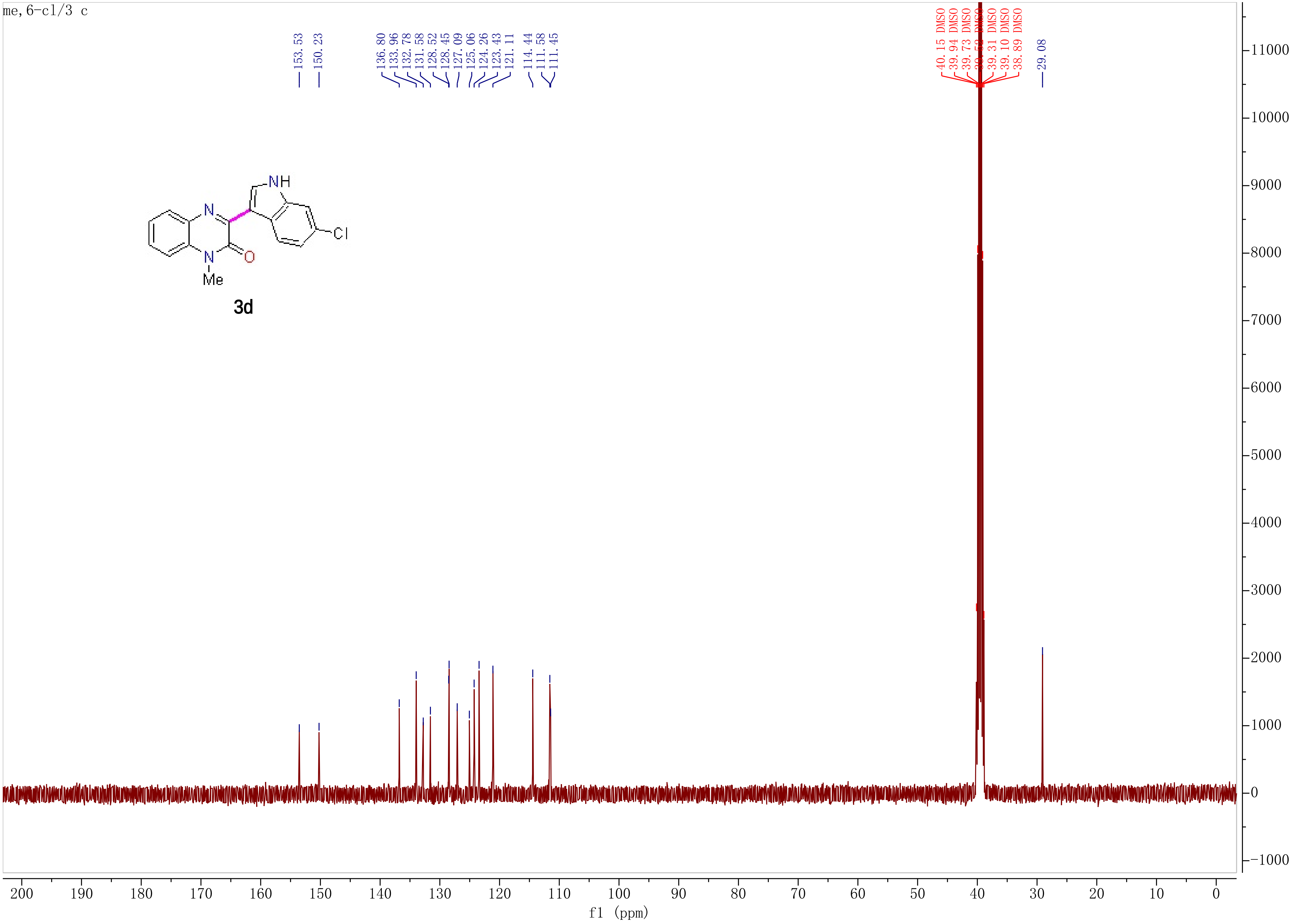

me,6-cl/3 c
153.53
150.23
136.80
133.96
132.78
131.58
128.52
128.45
127.09
125.06
124.26
123.43
121.11
114.44
111.58
111.45
40.15 DMSO
39.94 DMSO
39.73 DMSO
39.52 DMSO
39.31 DMSO
39.10 DMSO
38.89 DMSO
29.08
NH
N
N
O
Cl
Me
3d
200
190
180
170
160
150
140
130
120
110
100
90
80
70
60
50
40
30
20
10
0
f1 (ppm)
11000
10000
9000
8000
7000
6000
5000
4000
3000
2000
1000
0
−1000

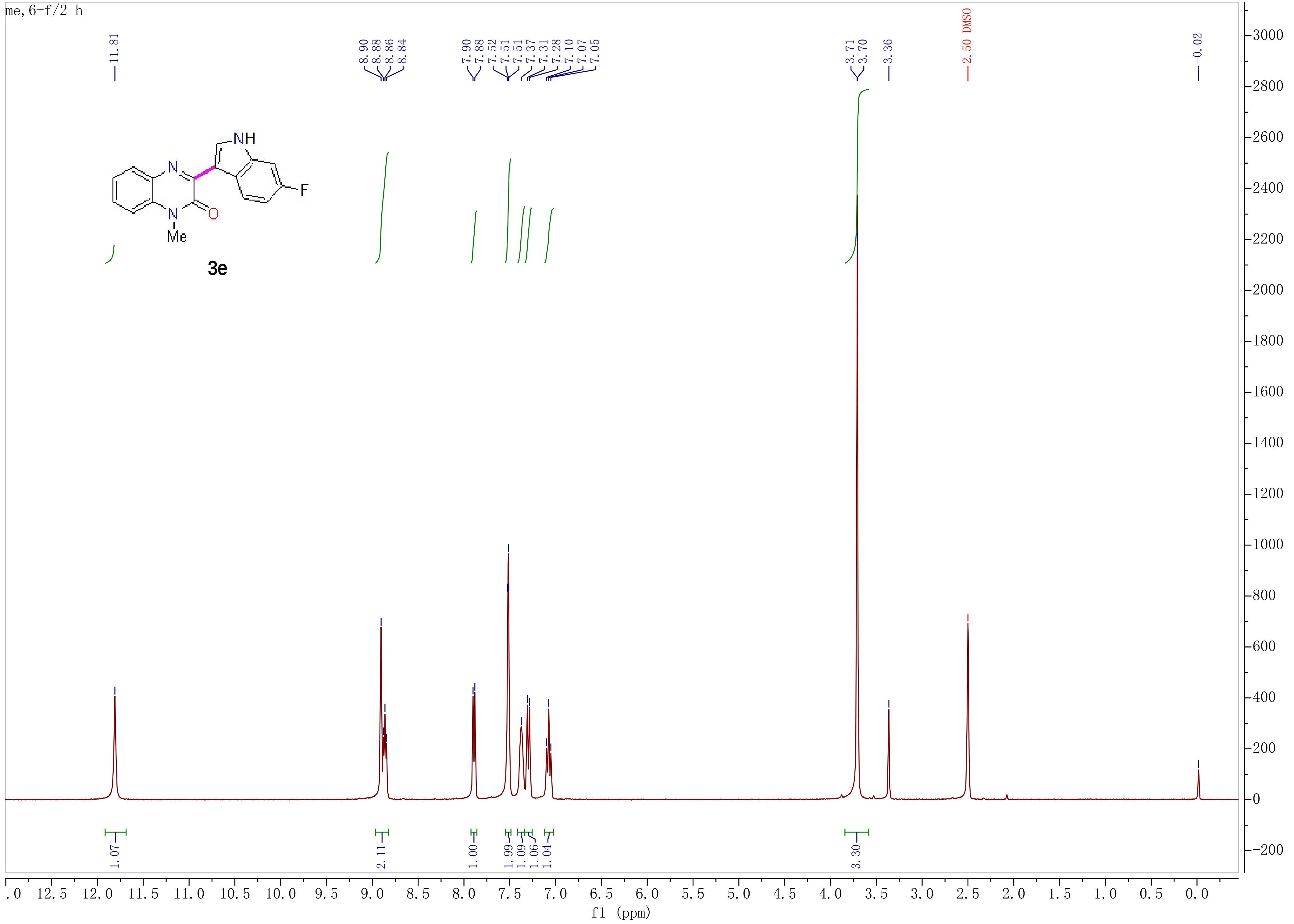
me,6-f/2 h
11.81
8.90
8.88
8.86
8.84
7.90
7.88
7.52
7.51
7.51
7.37
7.31
7.28
7.10
7.07
7.05
3.71
3.70
3.36
2.50 DMSO
-0.02
NH
N
F
N
O
Me
3e
1.07
2.11
1.00
1.99
1.09
1.06
1.04
3.30
f1 (ppm)

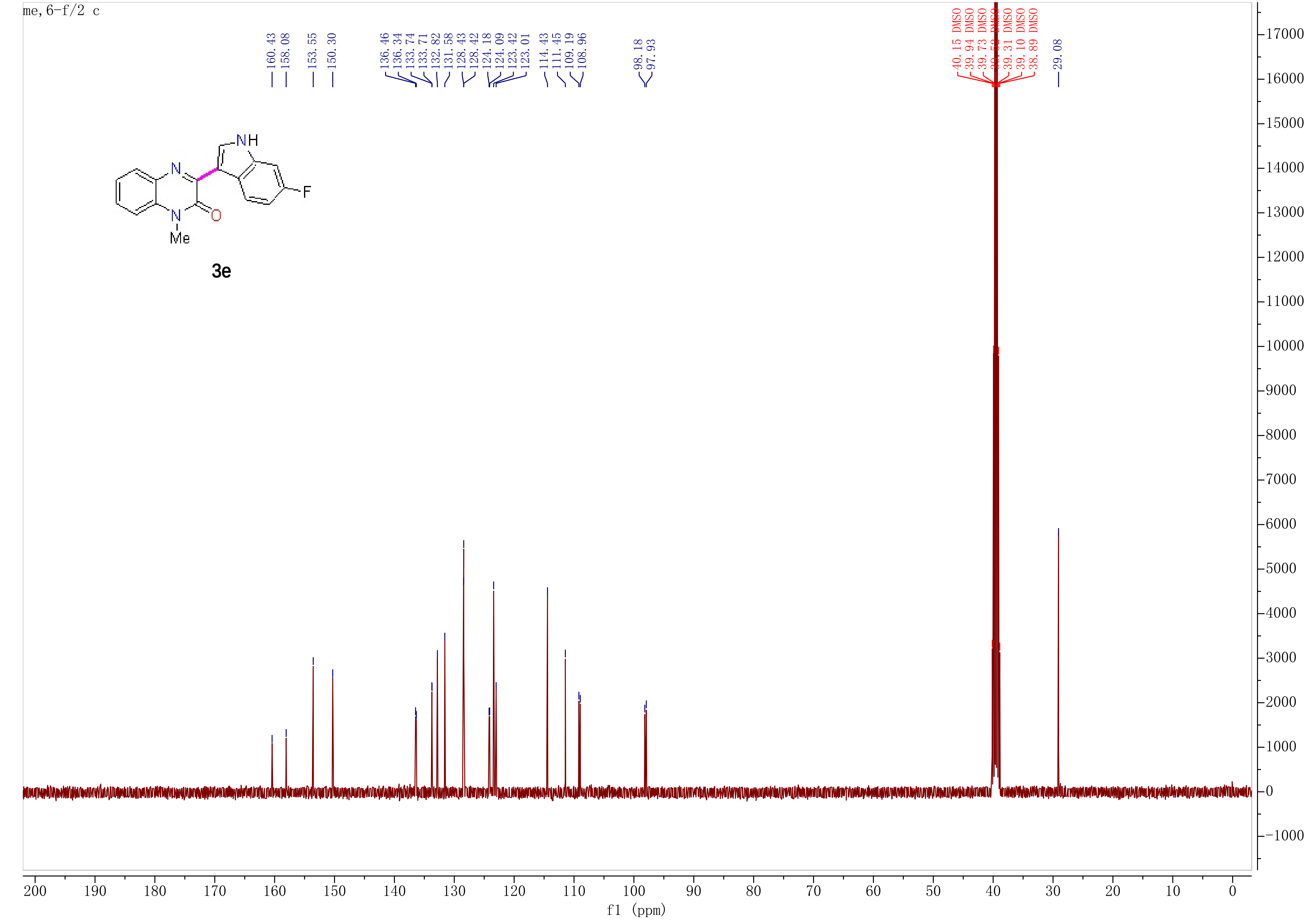
me,6-f/2 c
160.43
158.08
153.55
150.30
136.46
136.34
133.74
133.71
132.82
131.58
128.43
128.42
124.18
124.09
123.42
123.01
114.43
111.45
109.19
108.96
98.18
97.93
40.15 DMSO
39.94 DMSO
39.73 DMSO
39.52 DMSO
39.31 DMSO
39.10 DMSO
38.89 DMSO
29.08
NH
N
F
N
O
Me
3e
f1 (ppm)

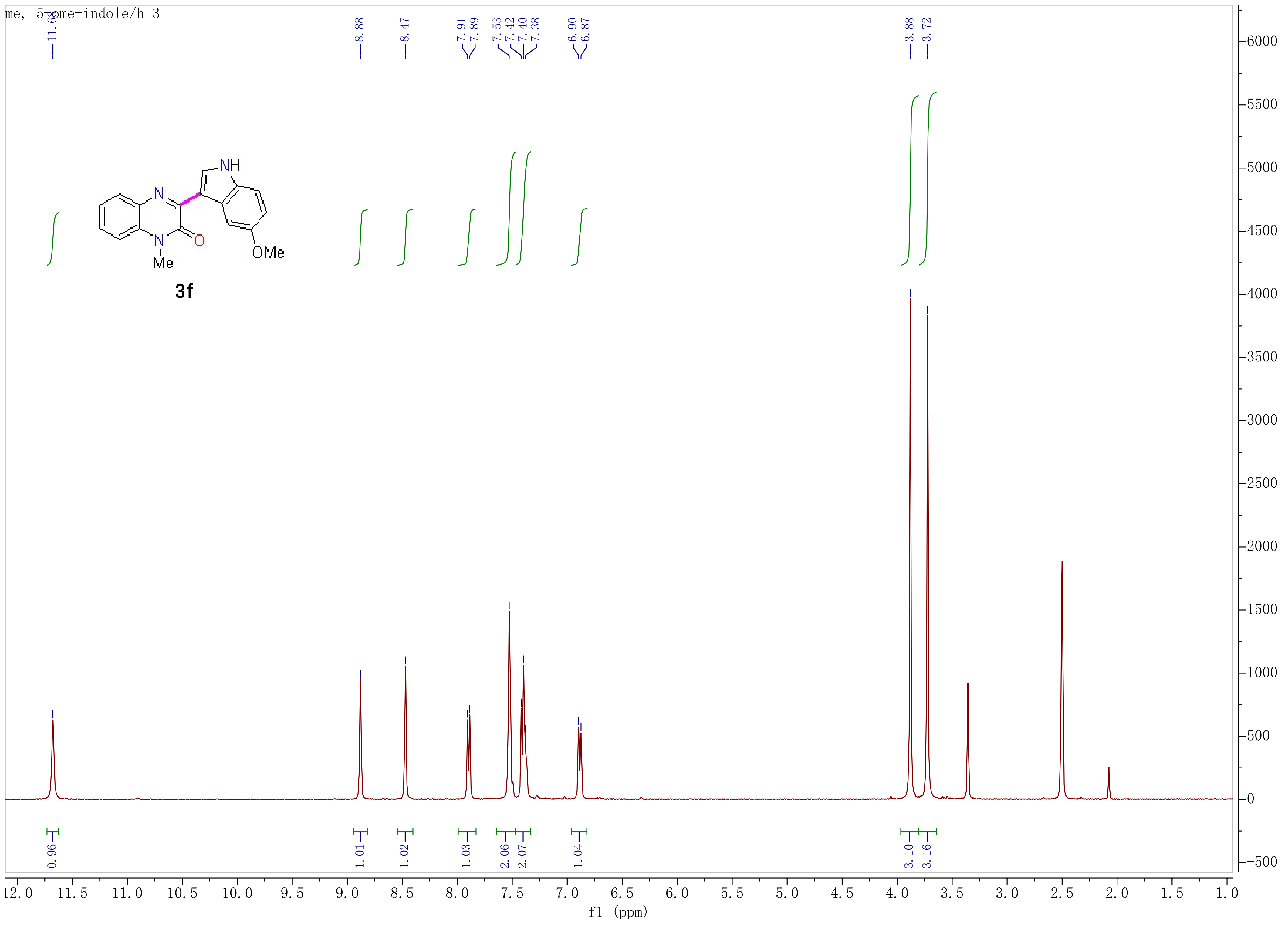
me, 5-ome-indole/h 3
11.69
8.88
8.47
7.91
7.89
7.53
7.42
7.40
7.38
6.90
6.87
3.88
3.72
NH
N
N
O
Me
OMe
3f
0.96
1.01
1.02
1.03
2.06
2.07
1.04
3.10
3.16
6000
5500
5000
4500
4000
3500
3000
2500
2000
1500
1000
500
0
-500
12.0 11.5 11.0 10.5 10.0 9.5 9.0 8.5 8.0 7.5 7.0 6.5 6.0 5.5 5.0 4.5 4.0 3.5 3.0 2.5 2.0 1.5 1.0
f1 (ppm)

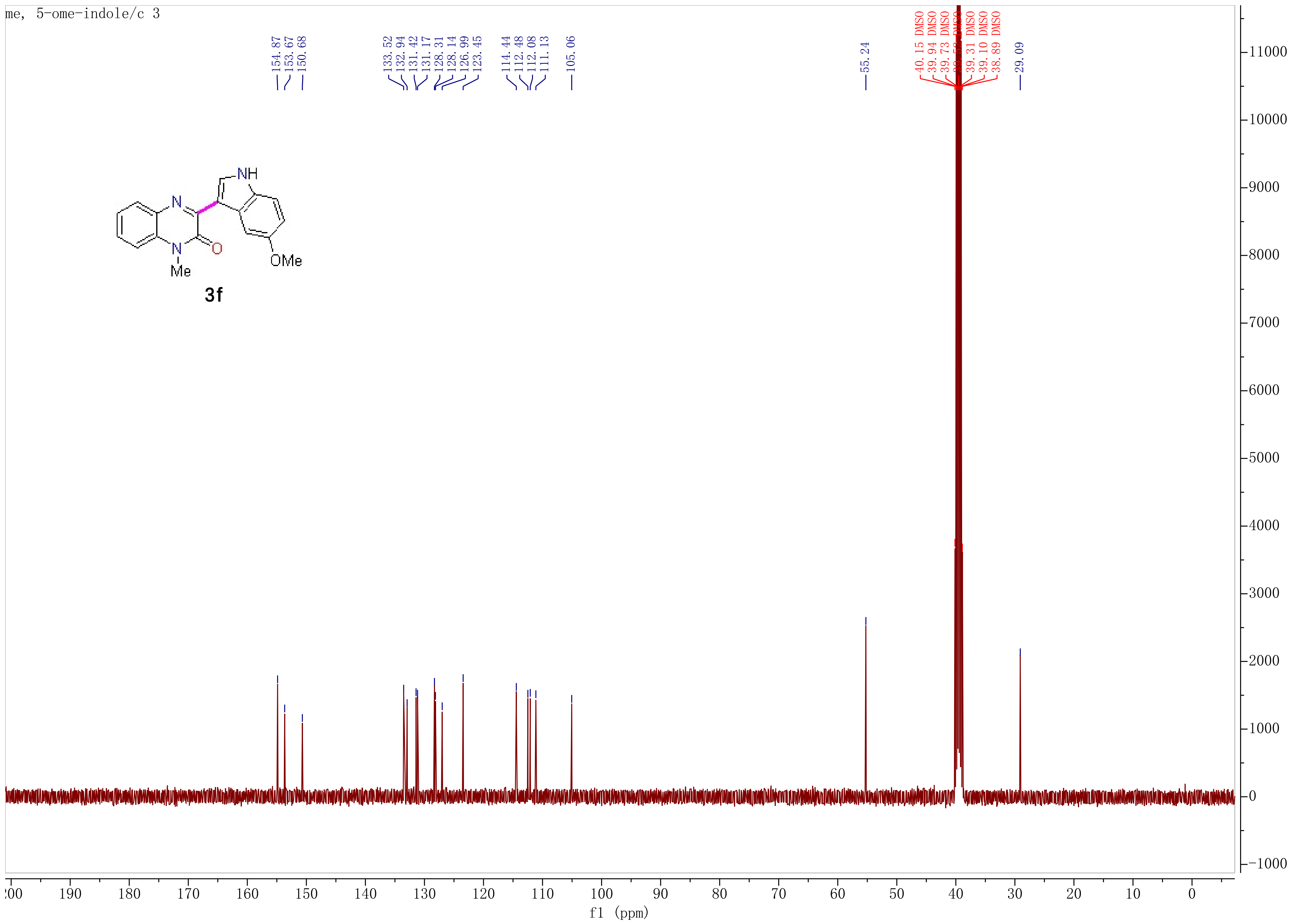
me, 5-ome-indole/c 3
154.87
153.67
150.68
133.52
132.94
131.42
131.17
128.31
128.14
126.99
123.45
114.44
112.48
112.08
111.13
105.06
55.24
40.15 DMSO
39.94 DMSO
39.73 DMSO
39.31 DMSO
39.10 DMSO
38.89 DMSO
29.09
NH
N
N
O
Me
OMe
3f
f1 (ppm)

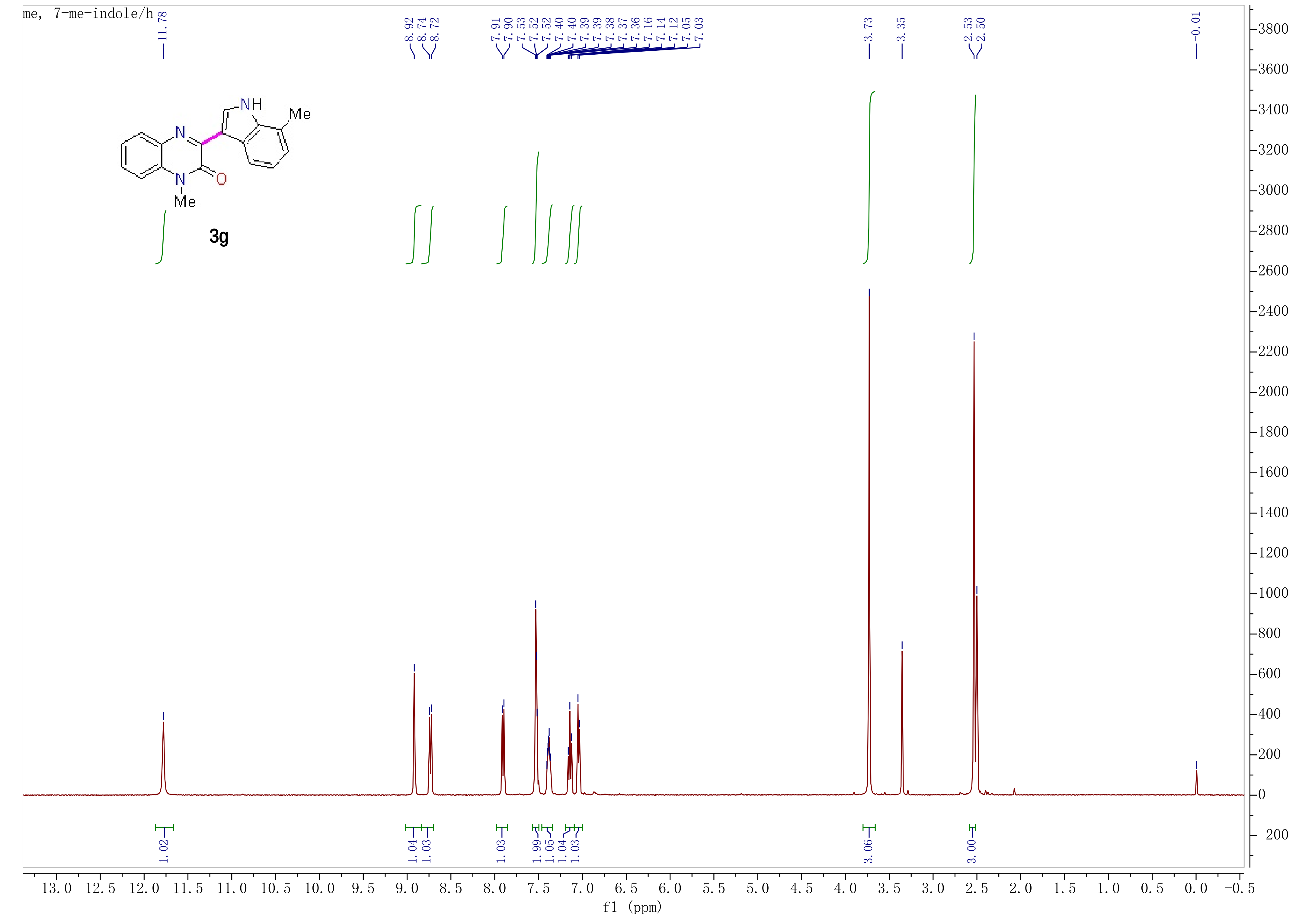
me, 7-me-indole/h
11.78
8.92 8.74 8.72
7.91 7.90 7.53 7.52 7.52 7.40 7.39 7.39 7.38 7.37 7.36 7.16 7.14 7.12 7.05 7.03
3.73
3.35
2.53 2.50
-0.01
NH
Me
N
N
O
Me
3g
1.02
1.04 1.03
1.03
1.99 1.05 1.04 1.03
3.06
3.00
f1 (ppm)

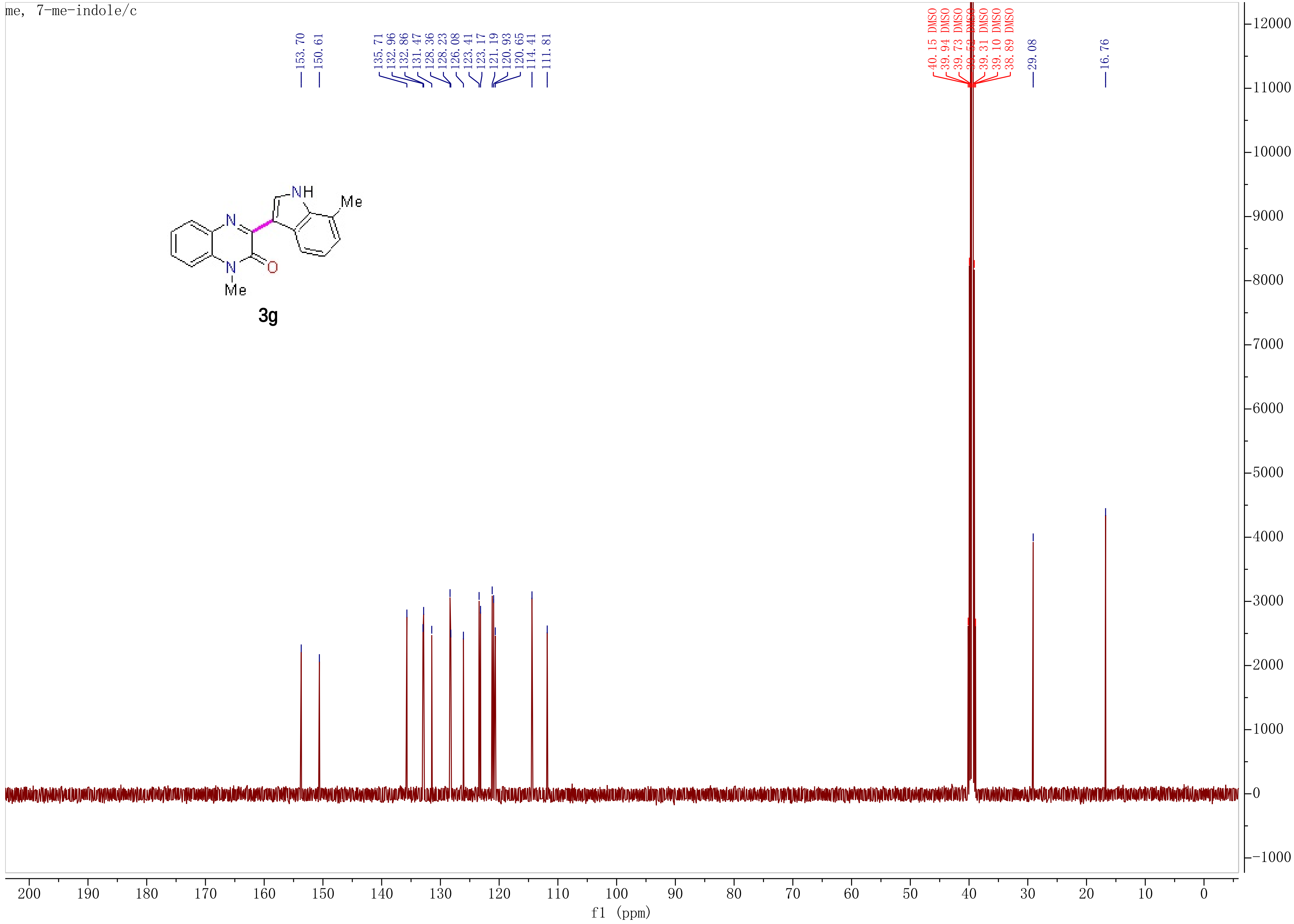
me, 7-me-indole/c
153.70
150.61
135.71
132.96
132.86
131.47
128.36
128.23
126.08
123.41
123.17
121.19
120.93
120.65
114.41
111.81
40.15 DMSO
39.94 DMSO
39.73 DMSO
39.31 DMSO
39.10 DMSO
38.89 DMSO
29.08
16.76
NH
Me
N
N
O
Me
3g
12000
11000
10000
9000
8000
7000
6000
5000
4000
3000
2000
1000
0
−1000
200
190
180
170
160
150
140
130
120
110
100
90
80
70
60
50
40
30
20
10
0
f1 (ppm)

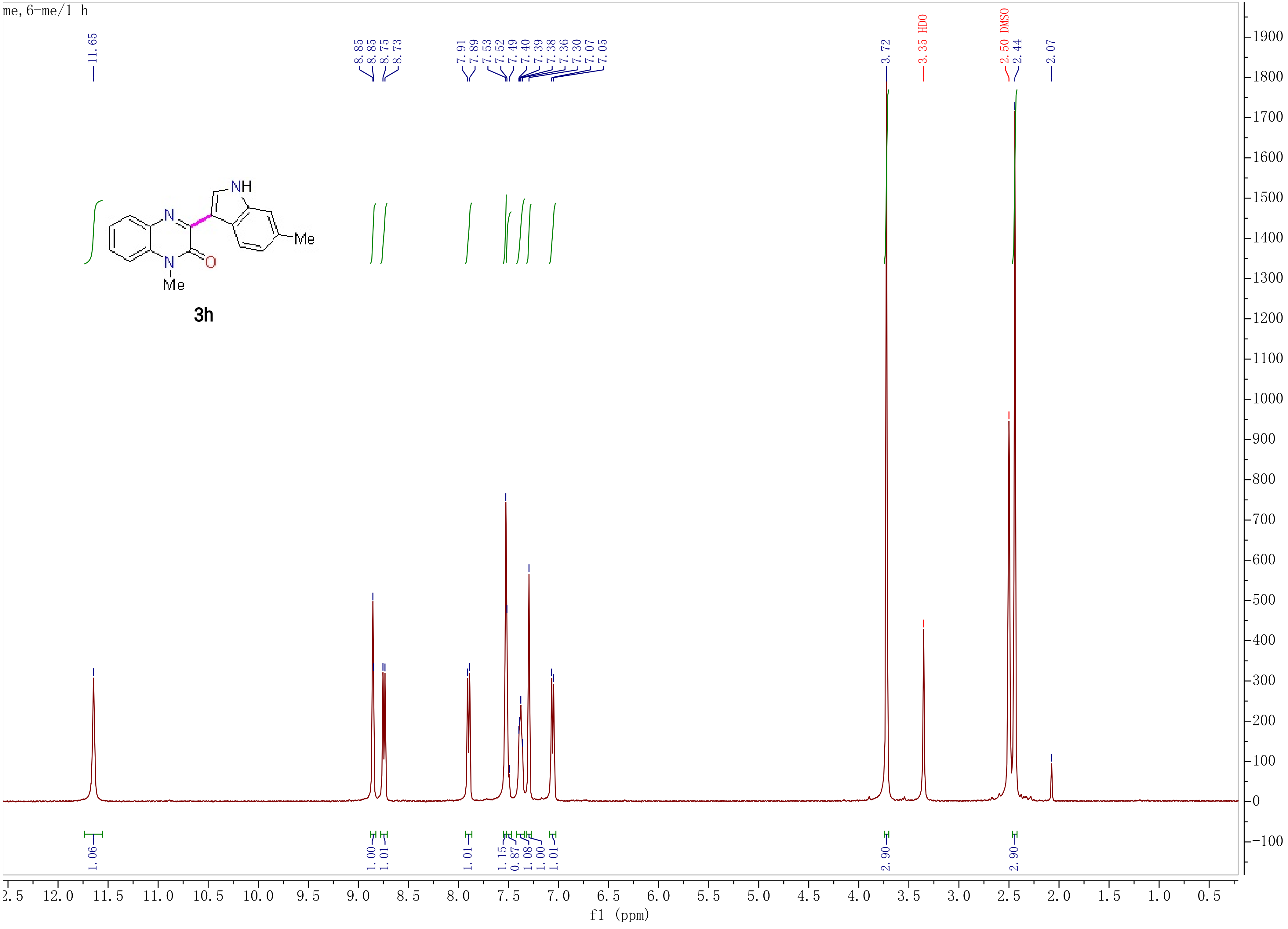
me,6-me/1 h
11.65
8.85 8.85 8.75 8.73
7.91 7.89 7.53 7.52 7.49 7.40 7.39 7.38 7.36 7.30 7.07 7.05
3.72
3.35 HDO
2.50 DMSO
2.44
2.07
NH
N
N
Me
O
Me
3h
1.06
1.00 1.01
1.01
1.15 0.87 1.08 1.00 1.01
2.90
2.90
f1 (ppm)

me,6-me/1 c

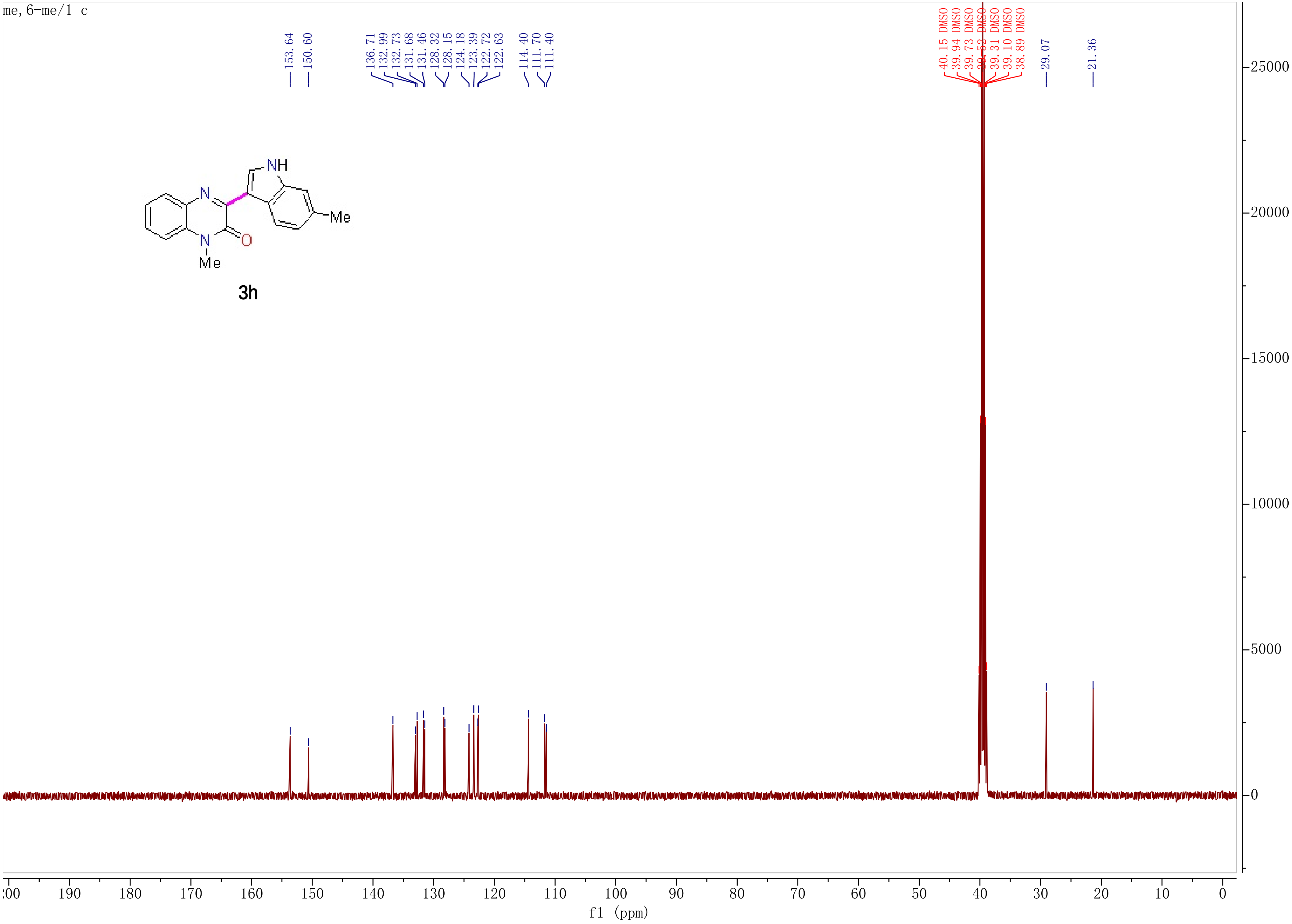
3h
153.64
150.60
136.71
132.99
132.73
131.68
131.46
128.32
128.15
124.18
123.39
122.72
122.63
114.40
111.70
111.40
40.15 DMSO
39.94 DMSO
39.73 DMSO
39.52 DMSO
39.31 DMSO
39.10 DMSO
38.89 DMSO
29.07
21.36
f1 (ppm)

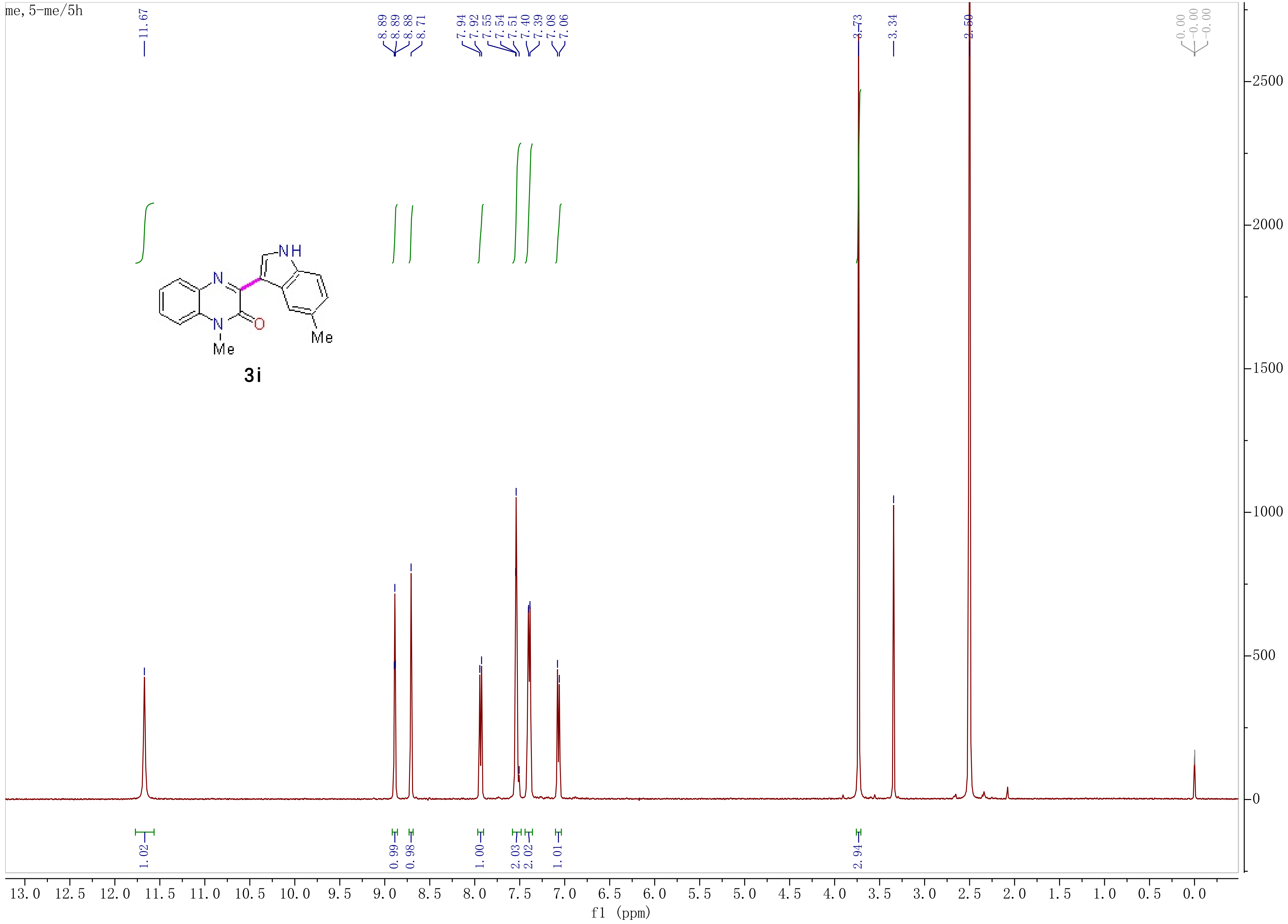
me,5-me/5h
11.67
8.89
8.89
8.88
8.71
7.94
7.92
7.55
7.54
7.51
7.40
7.39
7.08
7.06
3.73
3.34
2.50
0.00
-0.00
-0.00
NH
N
N
O
Me
Me
3i
1.02
0.99
0.98
1.00
2.03
2.02
1.01
2.94
2500
2000
1500
1000
500
0
13.0 12.5 12.0 11.5 11.0 10.5 10.0 9.5 9.0 8.5 8.0 7.5 7.0 6.5 6.0 5.5 5.0 4.5 4.0 3.5 3.0 2.5 2.0 1.5 1.0 0.5 0.0
f1 (ppm)

me,5-me.5.fid

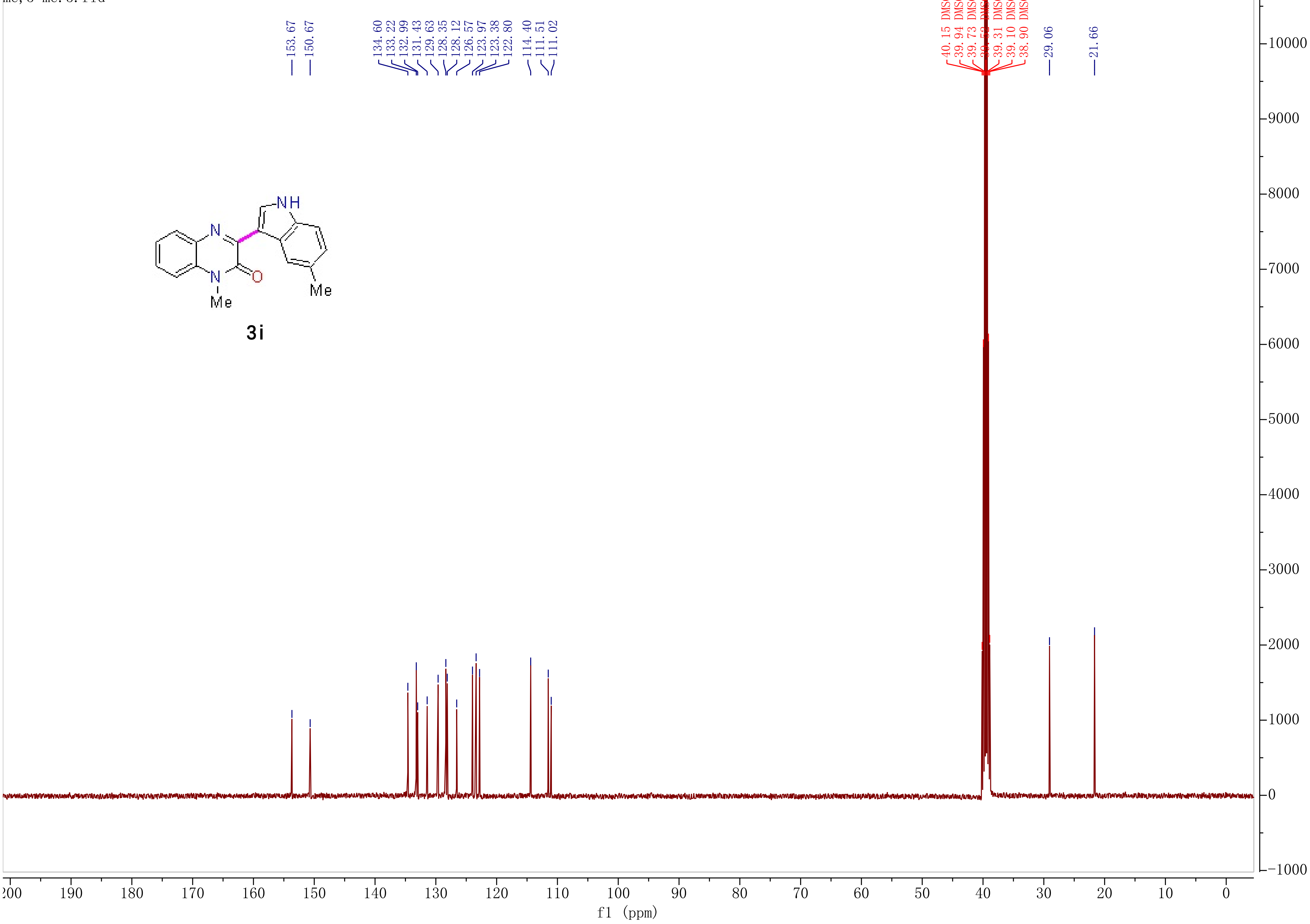

153.67
150.67
134.60
133.22
132.99
131.43
129.63
128.35
128.12
126.57
123.97
123.38
122.80
114.40
111.51
111.02
40.15 DMSO
39.94 DMSO
39.73 DMSO
39.31 DMSO
39.10 DMSO
38.90 DMSO
29.06
21.66
NH
N
N
O
Me
Me
3i
10000
9000
8000
7000
6000
5000
4000
3000
2000
1000
0
−1000
190
180
170
160
150
140
130
120
110
100
90
80
70
60
50
40
30
20
10
0
f1 (ppm)

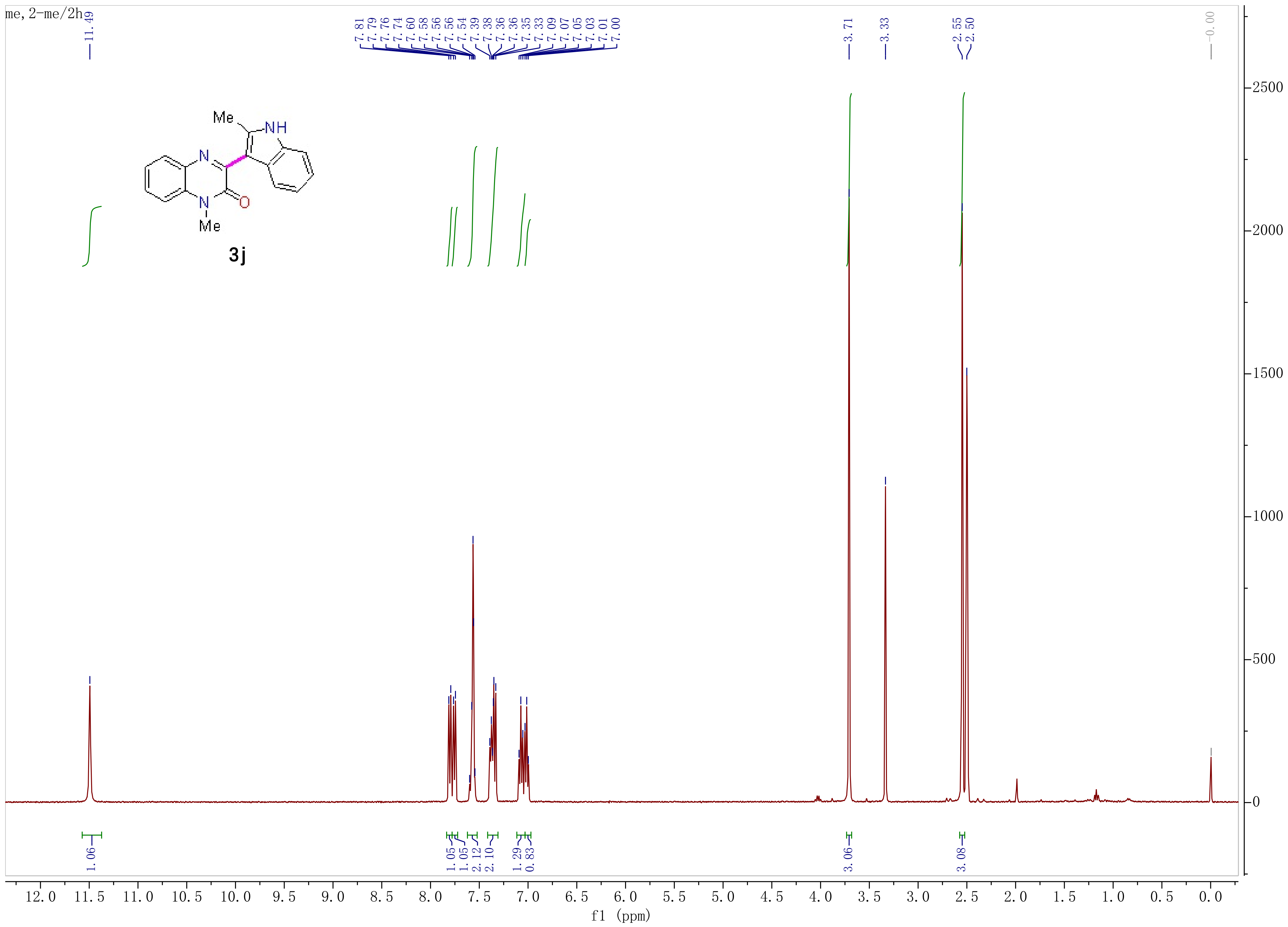
me,2-me/2h
11.49
7.81 7.79 7.76 7.74 7.60 7.58 7.56 7.56 7.54 7.39 7.38 7.36 7.36 7.35 7.33 7.09 7.07 7.05 7.03 7.01 7.00
3.71
3.33
2.55 2.50
-0.00
Me
NH
N
N
O
Me
3j
1.06
1.05 1.05 2.12 2.10 1.29 0.83
3.06
3.08
2500
2000
1500
1000
500
0
12.0 11.5 11.0 10.5 10.0 9.5 9.0 8.5 8.0 7.5 7.0 6.5 6.0 5.5 5.0 4.5 4.0 3.5 3.0 2.5 2.0 1.5 1.0 0.5 0.0
f1 (ppm)

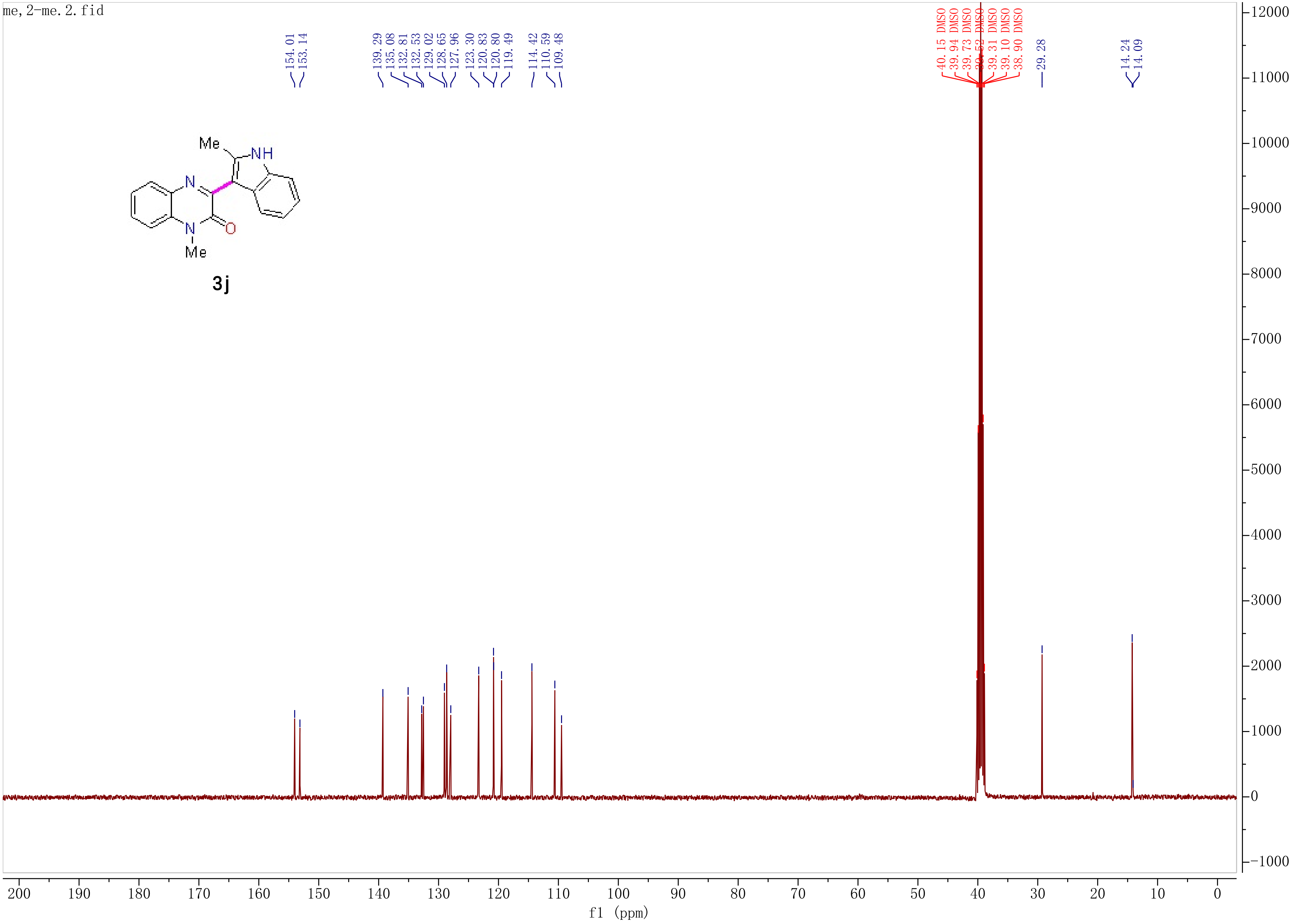

me,2-me.2.fid
154.01
153.14
139.29
135.08
132.81
132.53
129.02
128.65
127.96
123.30
120.83
120.80
119.49
114.42
110.59
109.48
40.15 DMSO
39.94 DMSO
39.73 DMSO
39.52 DMSO
39.31 DMSO
39.10 DMSO
38.90 DMSO
29.28
14.24
14.09
Me
NH
N
N
O
Me
3j
12000
11000
10000
9000
8000
7000
6000
5000
4000
3000
2000
1000
0
−1000
200 190 180 170 160 150 140 130 120 110 100 90 80 70 60 50 40 30 20 10 0
f1 (ppm)

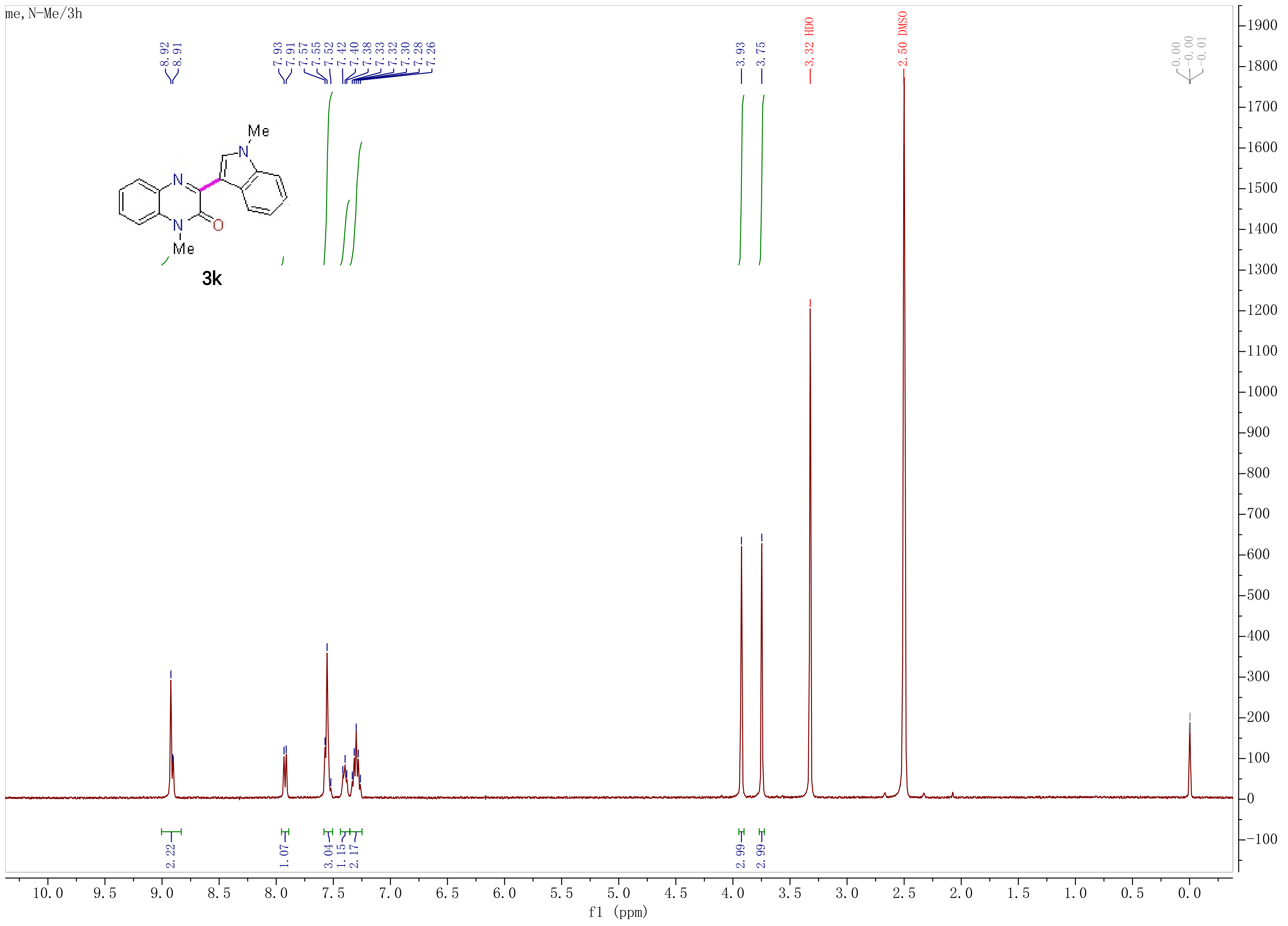
me,N-Me/3h
8.92
8.91
7.93
7.91
7.57
7.55
7.52
7.42
7.40
7.38
7.33
7.32
7.30
7.28
7.26
3.93
3.75
3.32 HDO
2.50 DMSO
0.00
-0.00
-0.01
Me
N
N
N
O
Me
3k
2.22
1.07
3.04
1.15
2.17
2.99
2.99
10.0
9.5
9.0
8.5
8.0
7.5
7.0
6.5
6.0
5.5
5.0
4.5
4.0
3.5
3.0
2.5
2.0
1.5
1.0
0.5
0.0
f1 (ppm)
1900
1800
1700
1600
1500
1400
1300
1200
1100
1000
900
800
700
600
500
400
300
200
100
0
-100

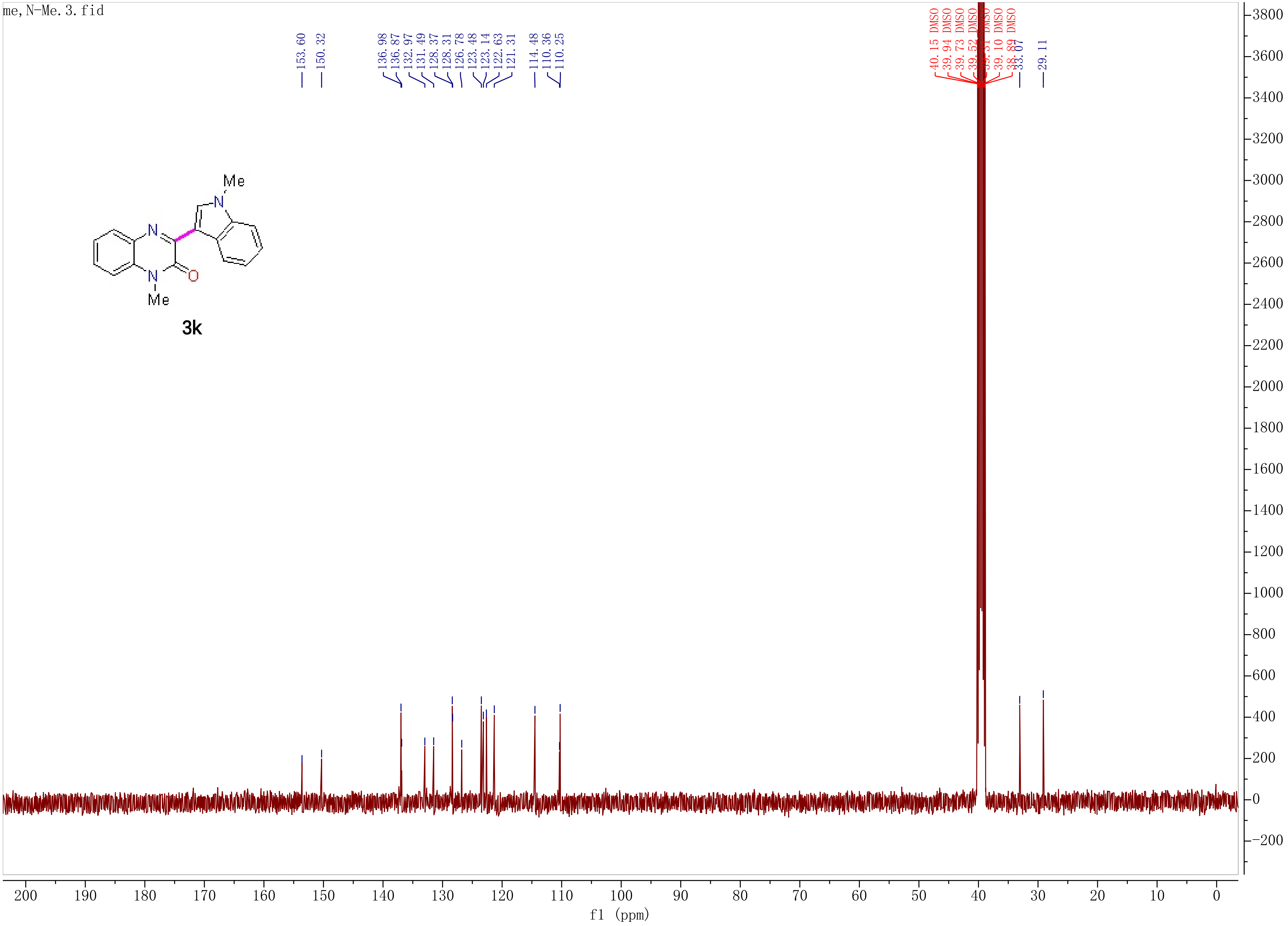

me,N-Me.3.fid
153.60
150.32
136.98
136.87
132.97
131.49
128.37
128.31
126.78
123.48
123.14
122.63
121.31
114.48
110.36
110.25
40.15 DMSO
39.94 DMSO
39.73 DMSO
39.52 DMSO
39.31 DMSO
39.10 DMSO
38.89 DMSO
33.07
29.11
Me
N
N
N
O
Me
3k
f1 (ppm)

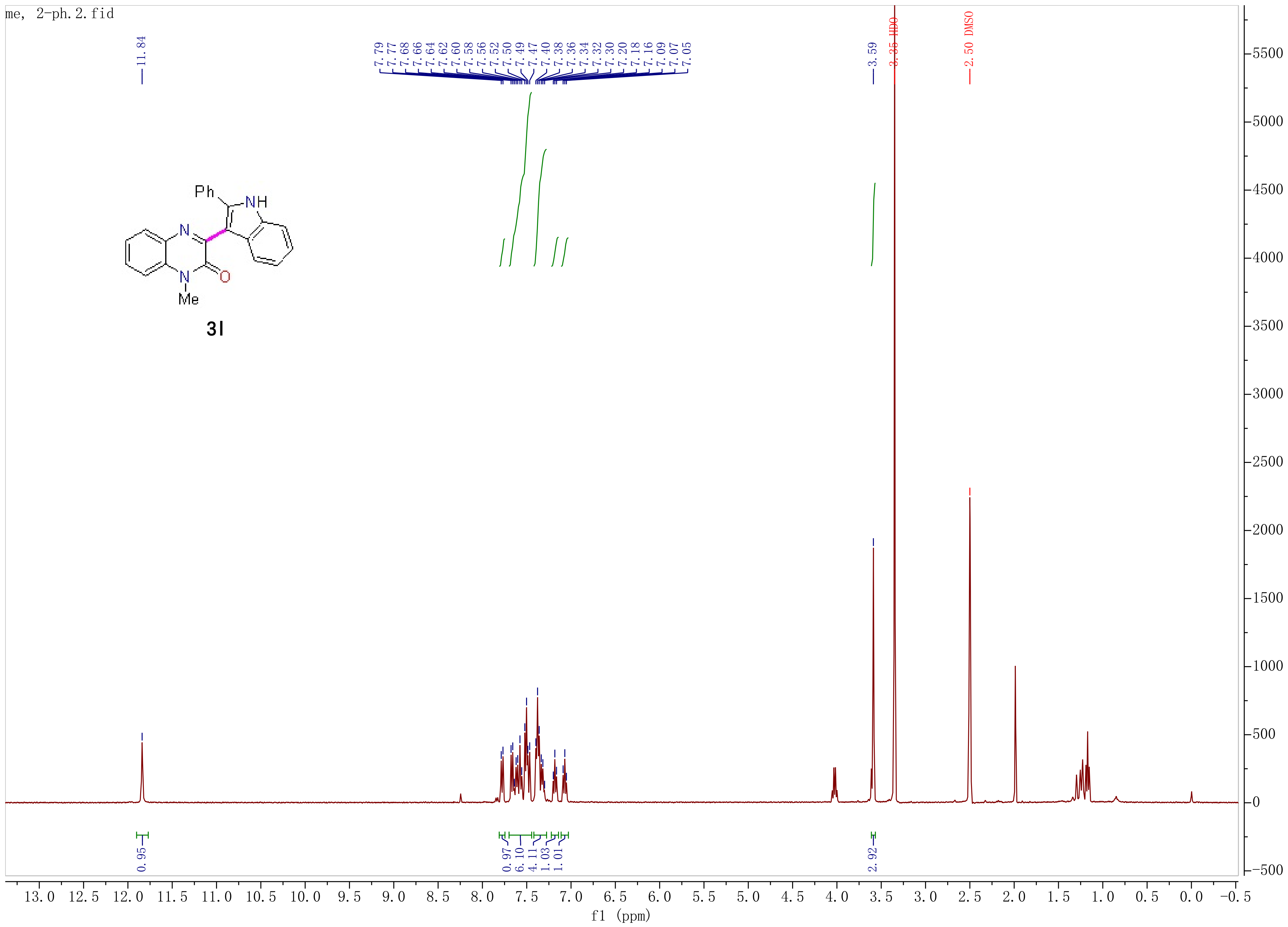
me, 2-ph.2.fid
11.84
7.79 7.77 7.68 7.66 7.64 7.62 7.60 7.58 7.56 7.52 7.50 7.49 7.47 7.40 7.38 7.36 7.34 7.32 7.30 7.20 7.18 7.16 7.09 7.07 7.05
3.59
3.35 HDO
2.50 DMSO
Ph
NH
N
N
O
Me
3l
0.95
0.97
6.10
4.11
1.03
1.01
2.92
f1 (ppm)
13.0 12.5 12.0 11.5 11.0 10.5 10.0 9.5 9.0 8.5 8.0 7.5 7.0 6.5 6.0 5.5 5.0 4.5 4.0 3.5 3.0 2.5 2.0 1.5 1.0 0.5 0.0 -0.5
5500 5000 4500 4000 3500 3000 2500 2000 1500 1000 500 0 -500

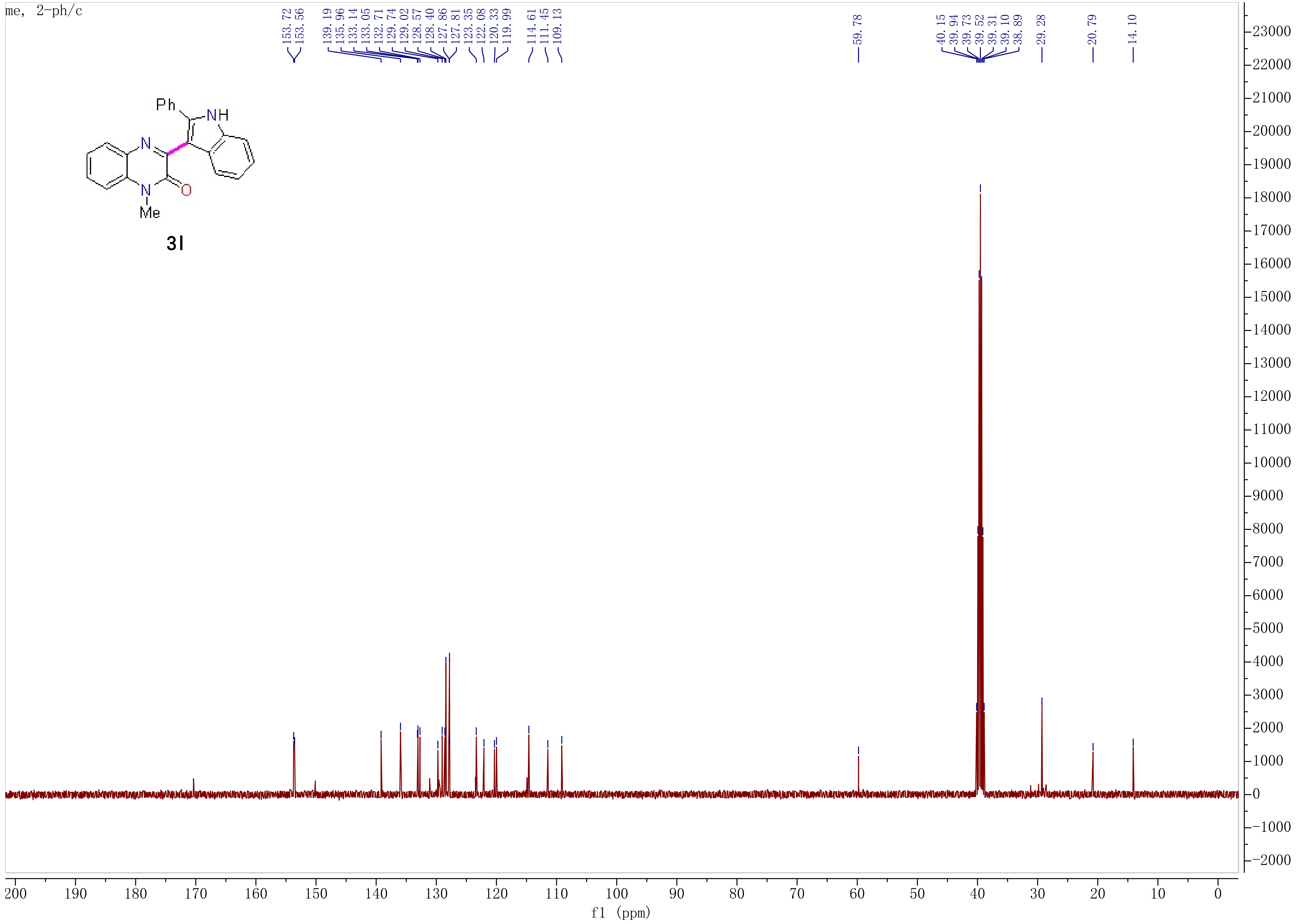
me, 2-ph/c
153.72
153.56
139.19
135.96
133.14
133.05
132.71
129.74
129.02
128.57
128.40
127.86
127.81
123.35
122.08
120.33
119.99
114.61
111.45
109.13
59.78
40.15
39.94
39.73
39.52
39.31
39.10
38.89
29.28
20.79
14.10
Ph
NH
N
N
O
Me
3l
f1 (ppm)

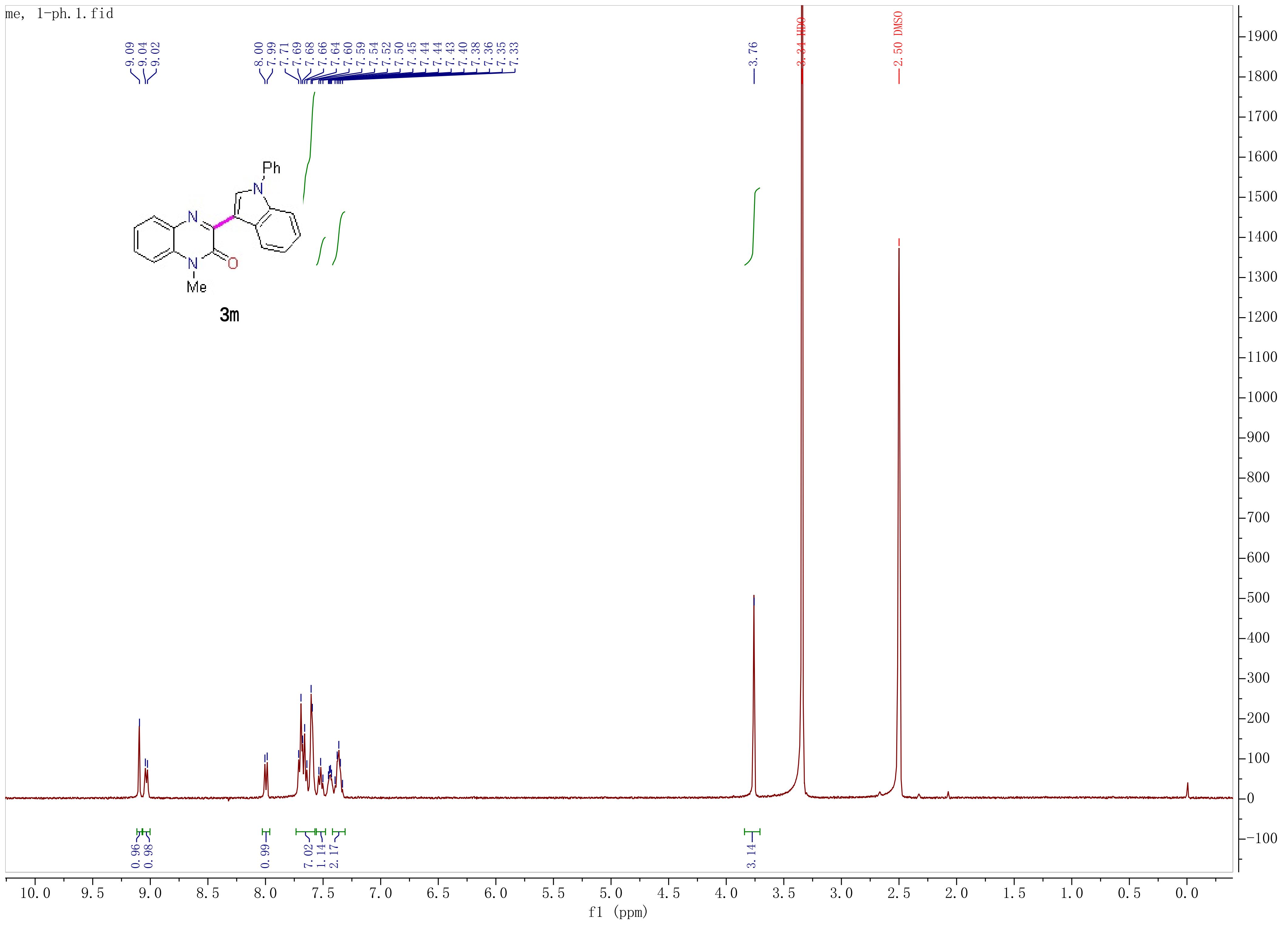
me, 1-ph.1.fid
9.09
9.04
9.02
8.00
7.99
7.71
7.69
7.68
7.66
7.64
7.60
7.59
7.54
7.52
7.50
7.45
7.44
7.44
7.43
7.40
7.38
7.36
7.35
7.33
3.76
3.34 HDO
2.50 DMSO
Ph
N
N
N
O
Me
3m
0.96
0.98
0.99
7.02
1.14
2.17
3.14
f1 (ppm)

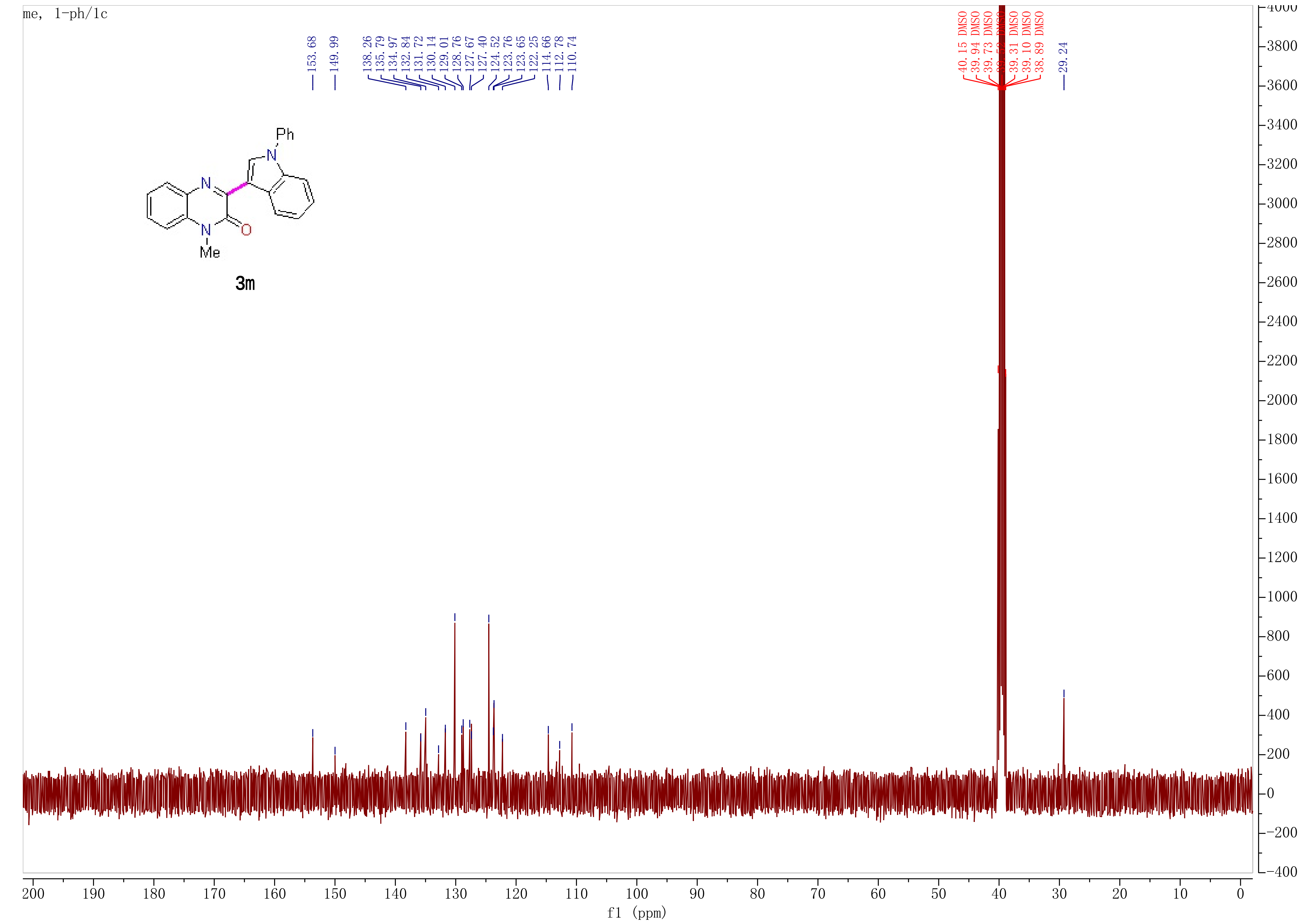
me, 1-ph/1c
153.68
149.99
138.26
135.79
134.97
132.84
131.72
130.14
129.01
128.76
127.67
127.40
124.52
123.76
123.65
122.25
114.66
112.78
110.74
40.15 DMSO
39.94 DMSO
39.73 DMSO
39.52 DMSO
39.31 DMSO
39.10 DMSO
38.89 DMSO
29.24
Ph
N
N
N
O
Me
3m
f1 (ppm)

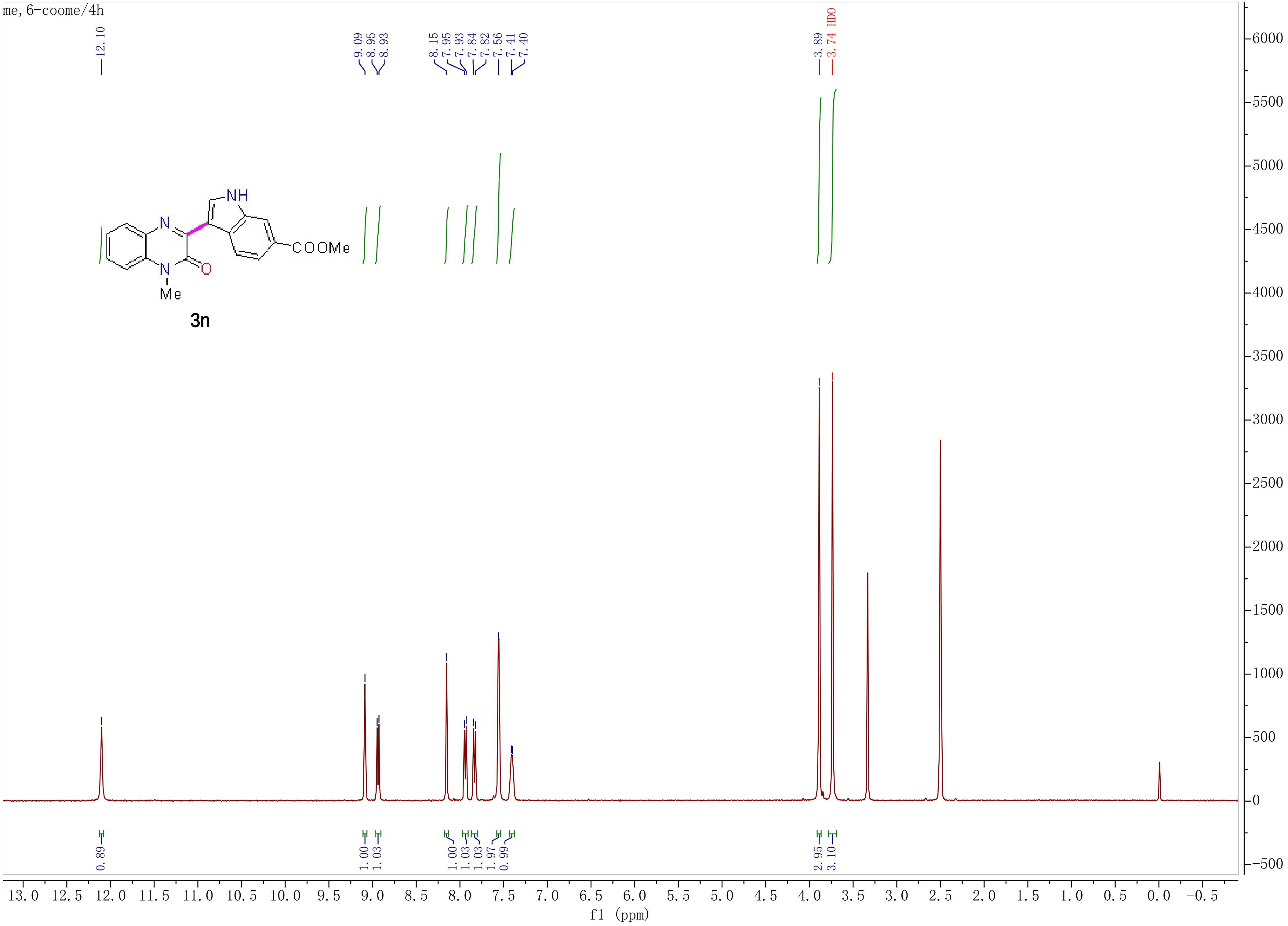

me,6-coome/4h
12.10
9.09
8.95
8.93
8.15
7.95
7.93
7.84
7.82
7.56
7.41
7.40
3.89
3.74 HDO
NH
N
N
O
Me
COOMe
3n
0.89
1.00
1.03
1.00
1.03
1.03
1.97
0.99
2.95
3.10
13.0 12.5 12.0 11.5 11.0 10.5 10.0 9.5 9.0 8.5 8.0 7.5 7.0 6.5 6.0 5.5 5.0 4.5 4.0 3.5 3.0 2.5 2.0 1.5 1.0 0.5 0.0 -0.5
f1 (ppm)
6000 5500 5000 4500 4000 3500 3000 2500 2000 1500 1000 500 0 -500

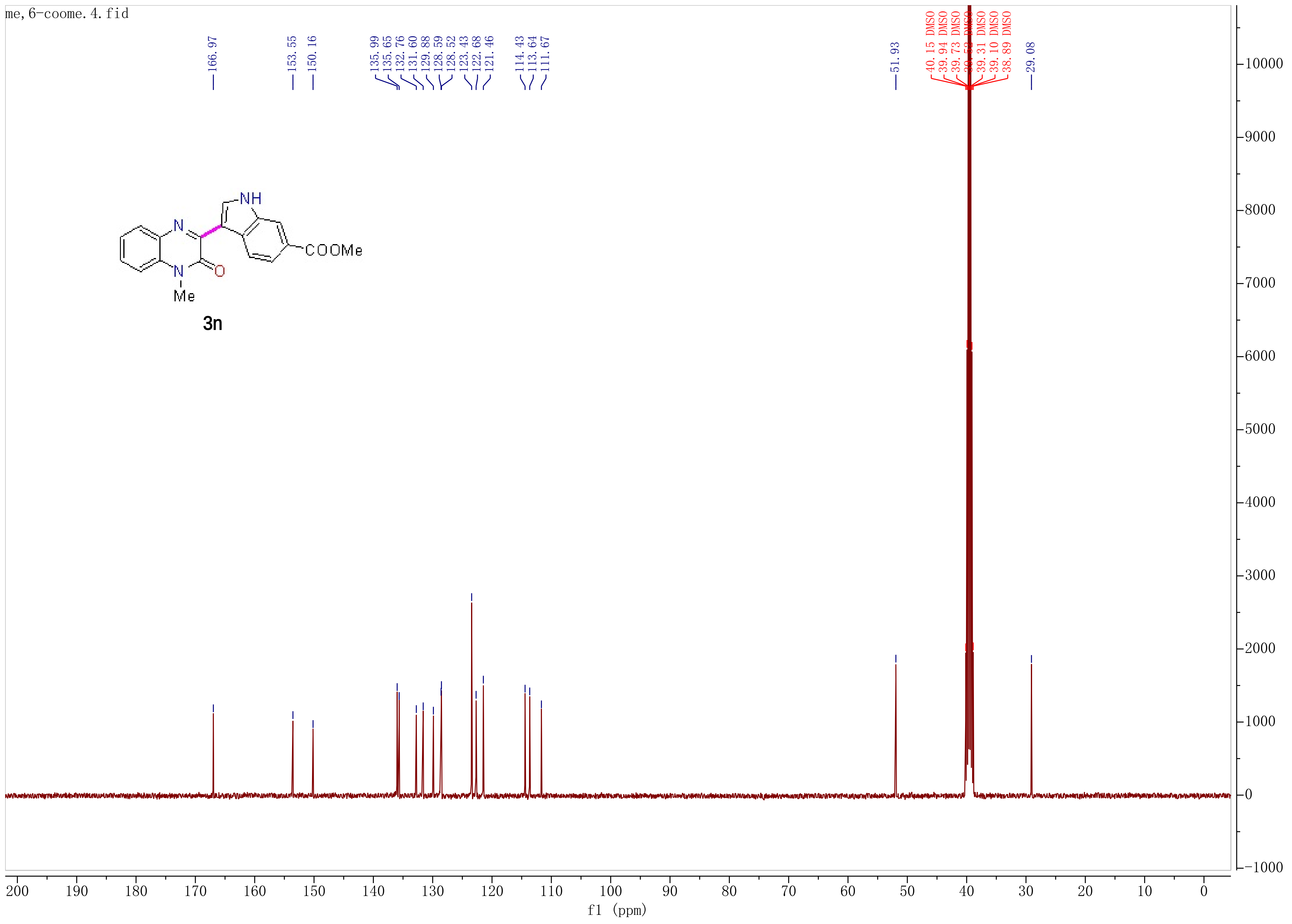
me,6-coome.4.fid
166.97
153.55
150.16
135.99
135.65
132.76
131.60
129.88
128.59
128.52
123.43
122.68
121.46
114.43
113.64
111.67
51.93
40.15 DMSO
39.94 DMSO
39.73 DMSO
39.31 DMSO
39.10 DMSO
38.89 DMSO
29.08
NH
N
N
O
Me
COOMe
3n
f1 (ppm)

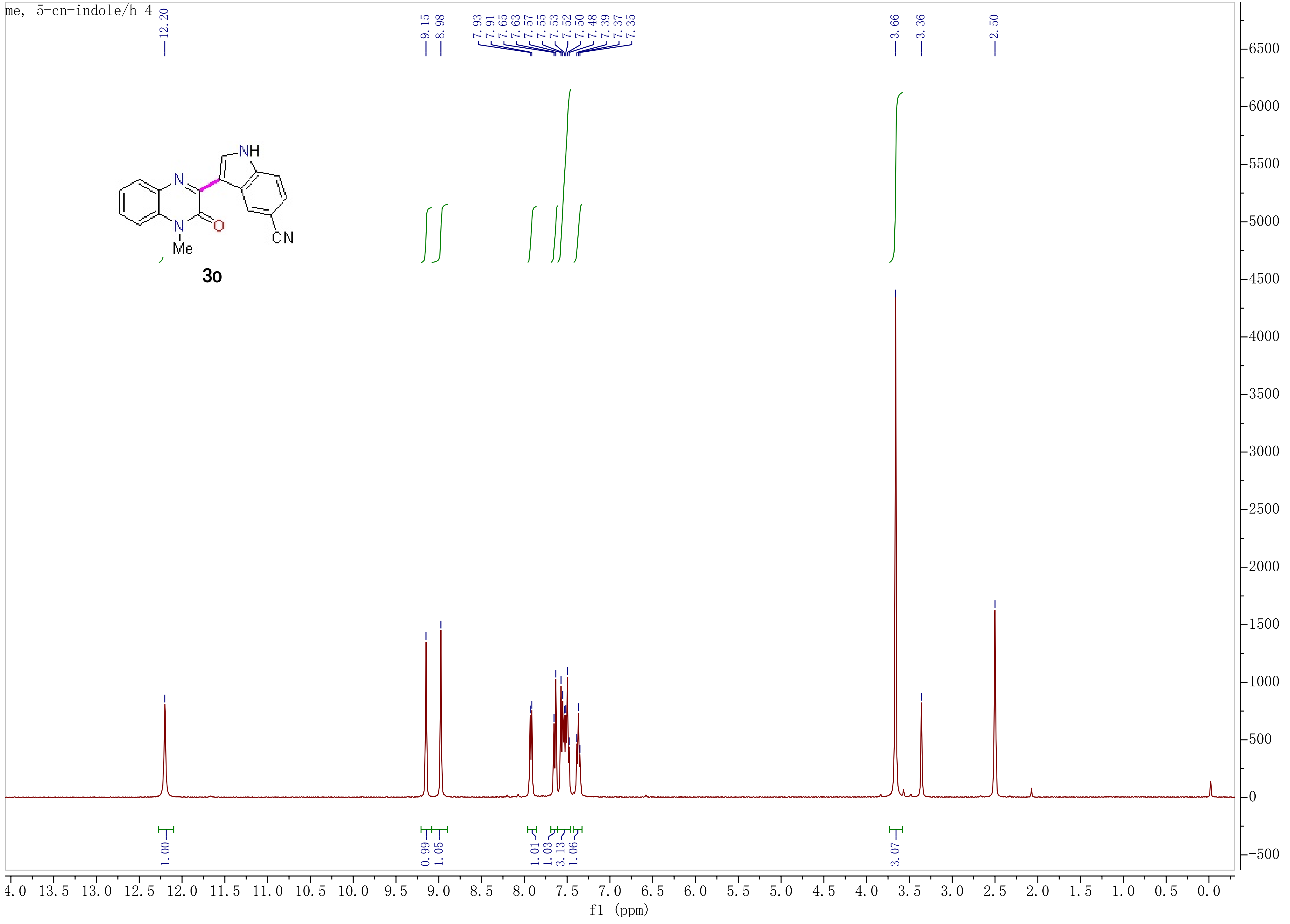
me, 5-cn-indole/h 4
12.20
9.15
8.98
7.93
7.91
7.65
7.63
7.57
7.55
7.53
7.52
7.50
7.48
7.39
7.37
7.35
3.66
3.36
2.50
NH
N
N
O
Me
CN
3o
1.00
0.99
1.05
1.01
1.03
3.13
1.06
3.07
f1 (ppm)

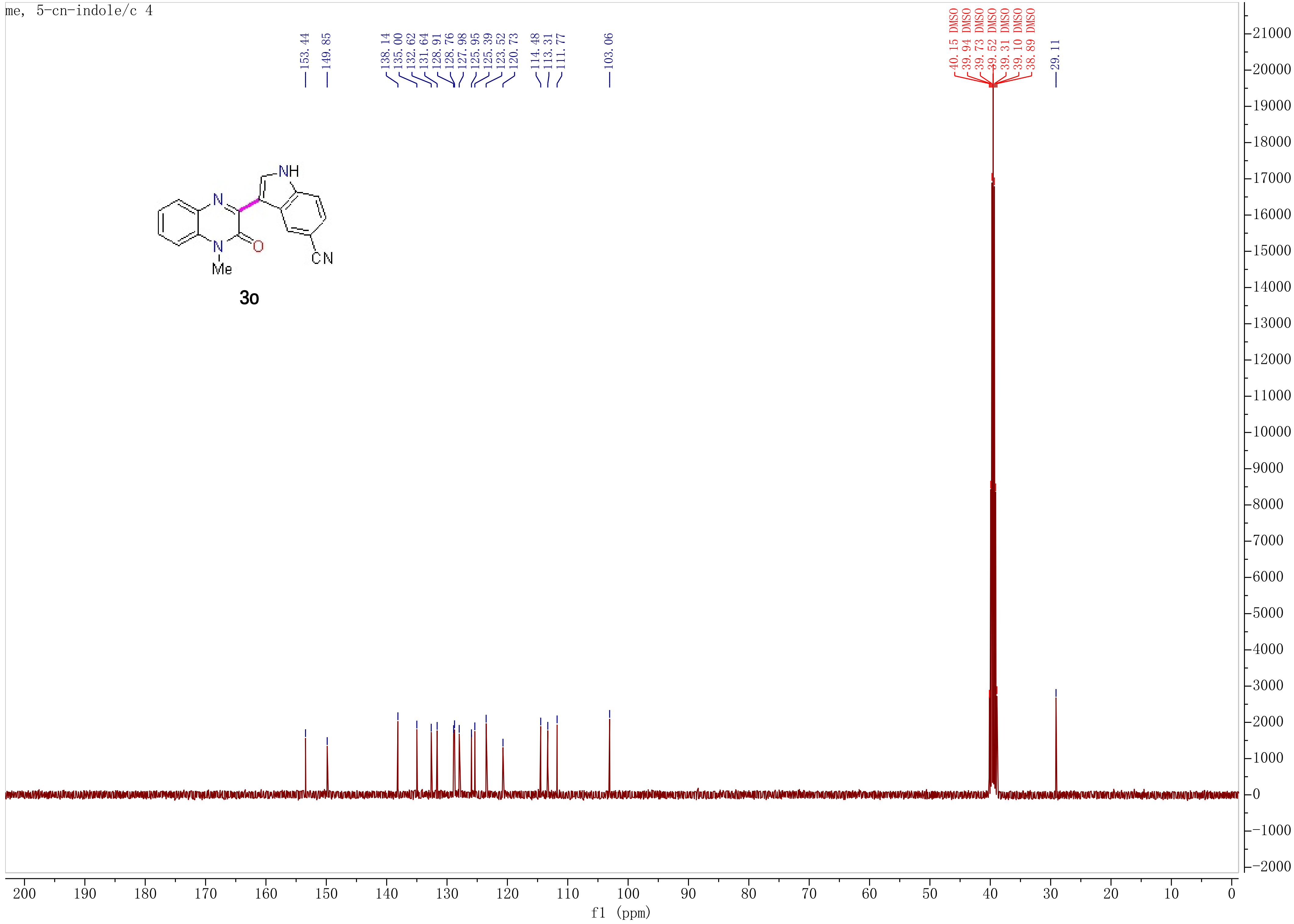
me, 5-cn-indole/c 4
153.44
149.85
138.14
135.00
132.62
131.64
128.91
128.76
127.98
125.95
125.39
123.52
120.73
114.48
113.31
111.77
103.06
40.15 DMSO
39.94 DMSO
39.73 DMSO
39.52 DMSO
39.31 DMSO
39.10 DMSO
38.89 DMSO
29.11
NH
N
N
O
Me
CN
3o
f1 (ppm)

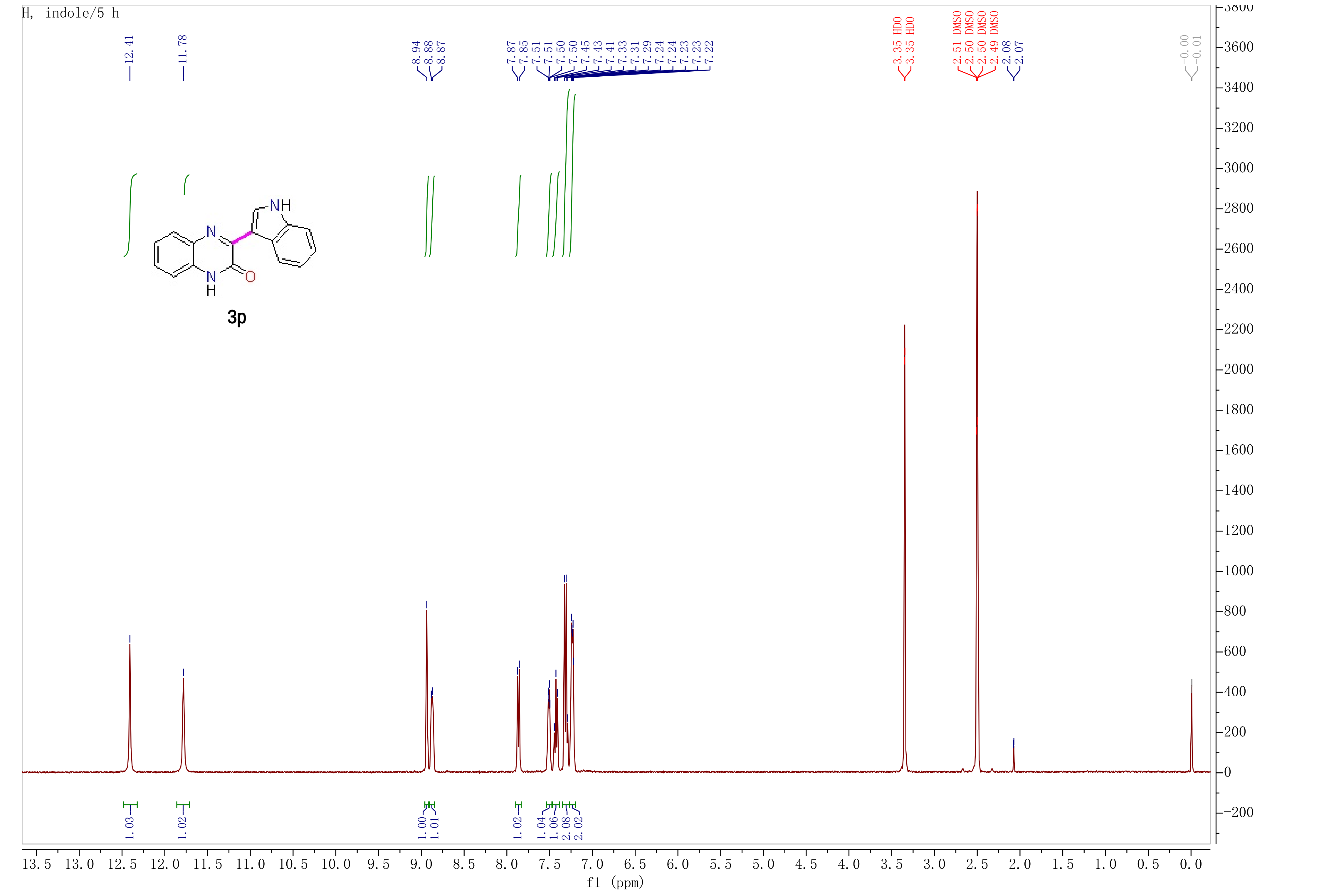

H, indole/5 h
12.41
11.78
8.94
8.88
8.87
7.87
7.85
7.51
7.51
7.50
7.50
7.45
7.43
7.41
7.33
7.31
7.29
7.24
7.24
7.23
7.23
7.22
3.35 HDO
3.35 HDO
2.51 DMSO
2.50 DMSO
2.50 DMSO
2.49 DMSO
2.08
2.07
-0.00
-0.01
NH
N
N
H
O
3p
1.03
1.02
1.00
1.01
1.02
1.04
1.06
2.08
2.02
f1 (ppm)

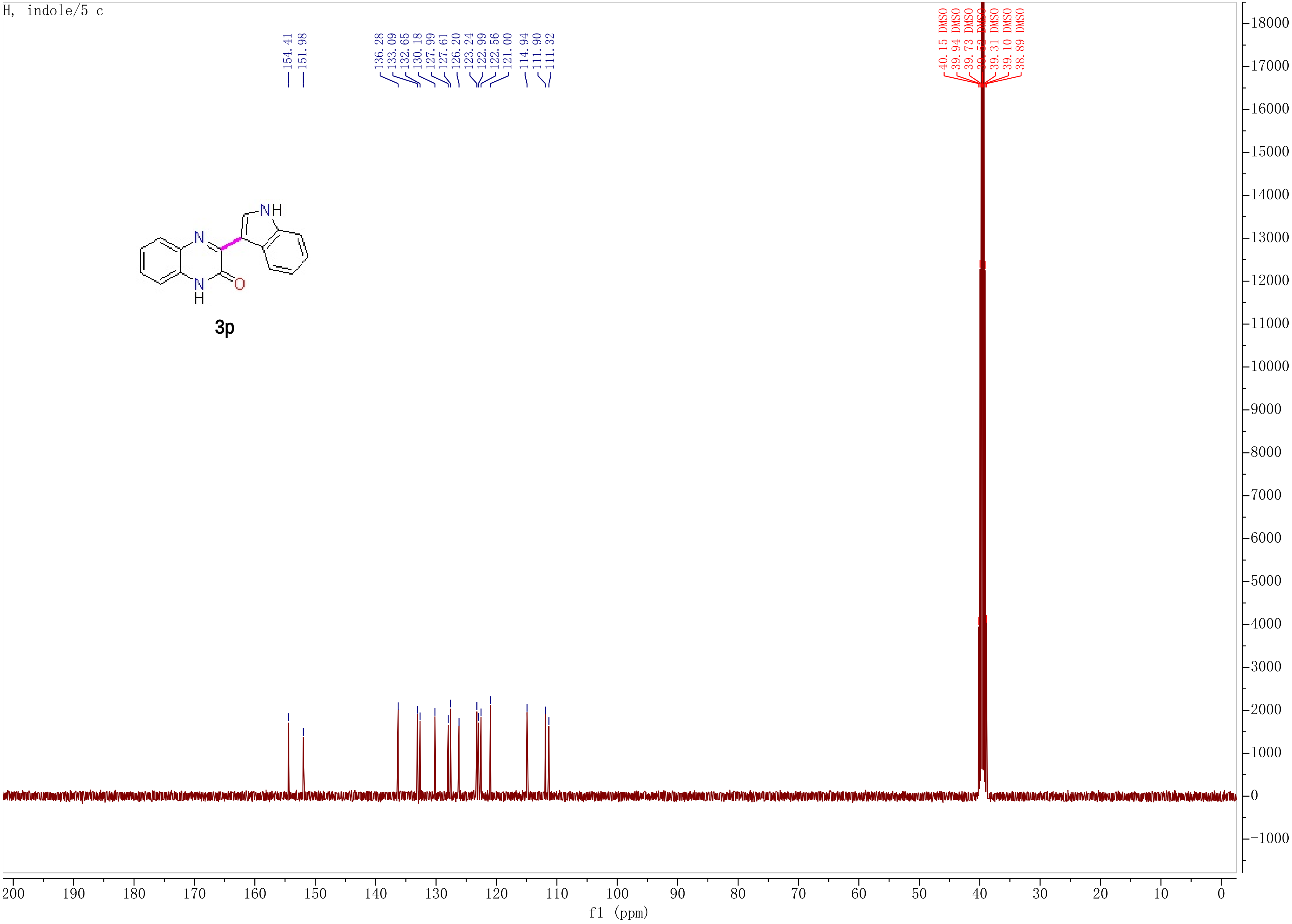

H, indole/5 c
154.41
151.98
136.28
133.09
132.65
130.18
127.99
127.61
126.20
123.24
122.99
122.56
121.00
114.94
111.90
111.32
40.15 DMSO
39.94 DMSO
39.73 DMSO
39.31 DMSO
39.10 DMSO
38.89 DMSO
NH
N
N
H
O
3p
f1 (ppm)

Et, indole/1 h

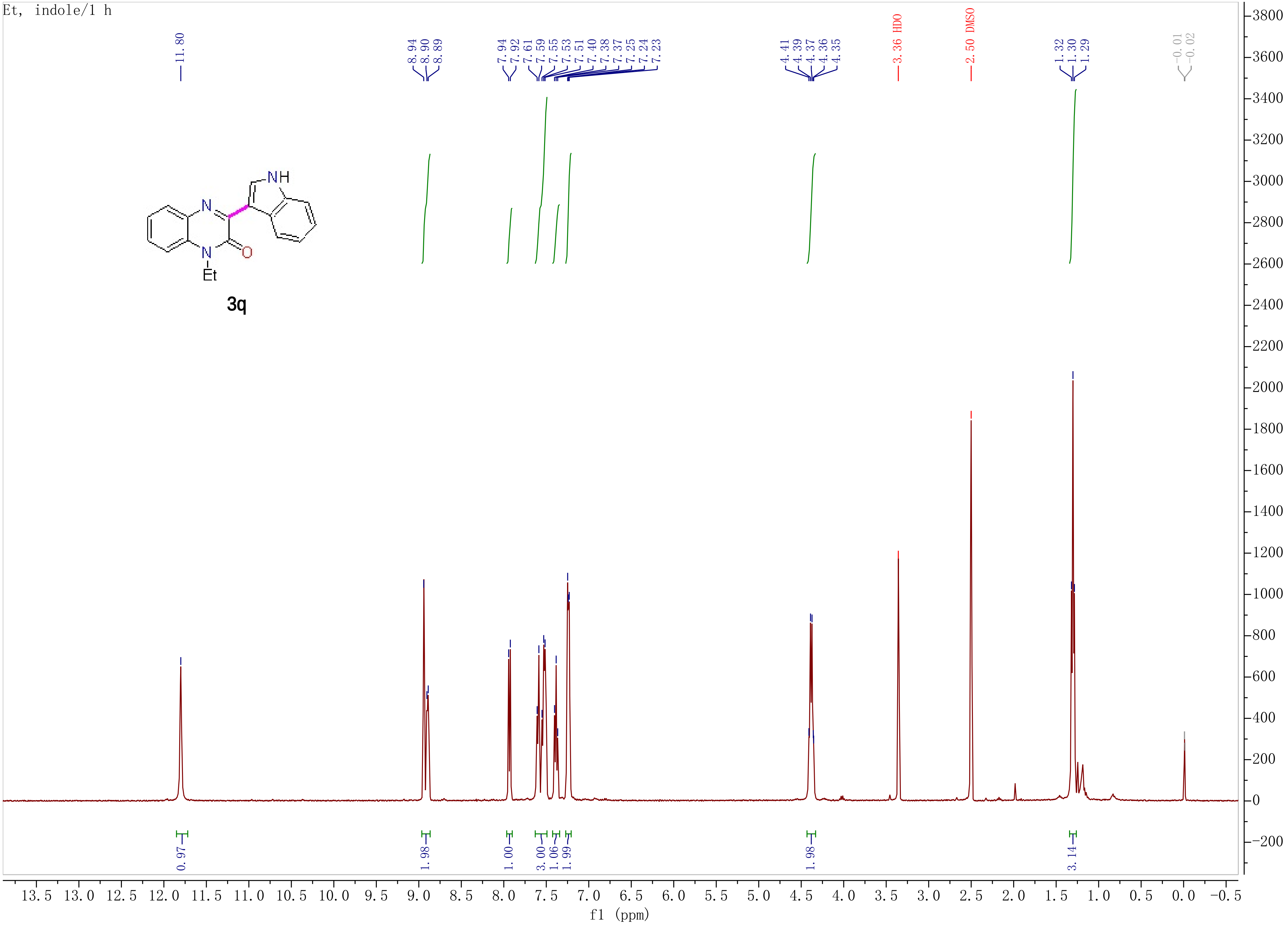

11.80
8.94
8.90
8.89
7.94
7.92
7.61
7.59
7.55
7.53
7.51
7.40
7.38
7.37
7.25
7.24
7.23
4.41
4.39
4.37
4.36
4.35
3.36 HDO
2.50 DMSO
1.32
1.30
1.29
-0.01
-0.02
NH
N
N
O
Et
3q
0.97
1.98
1.00
3.00
1.06
1.99
1.98
3.14
f1 (ppm)

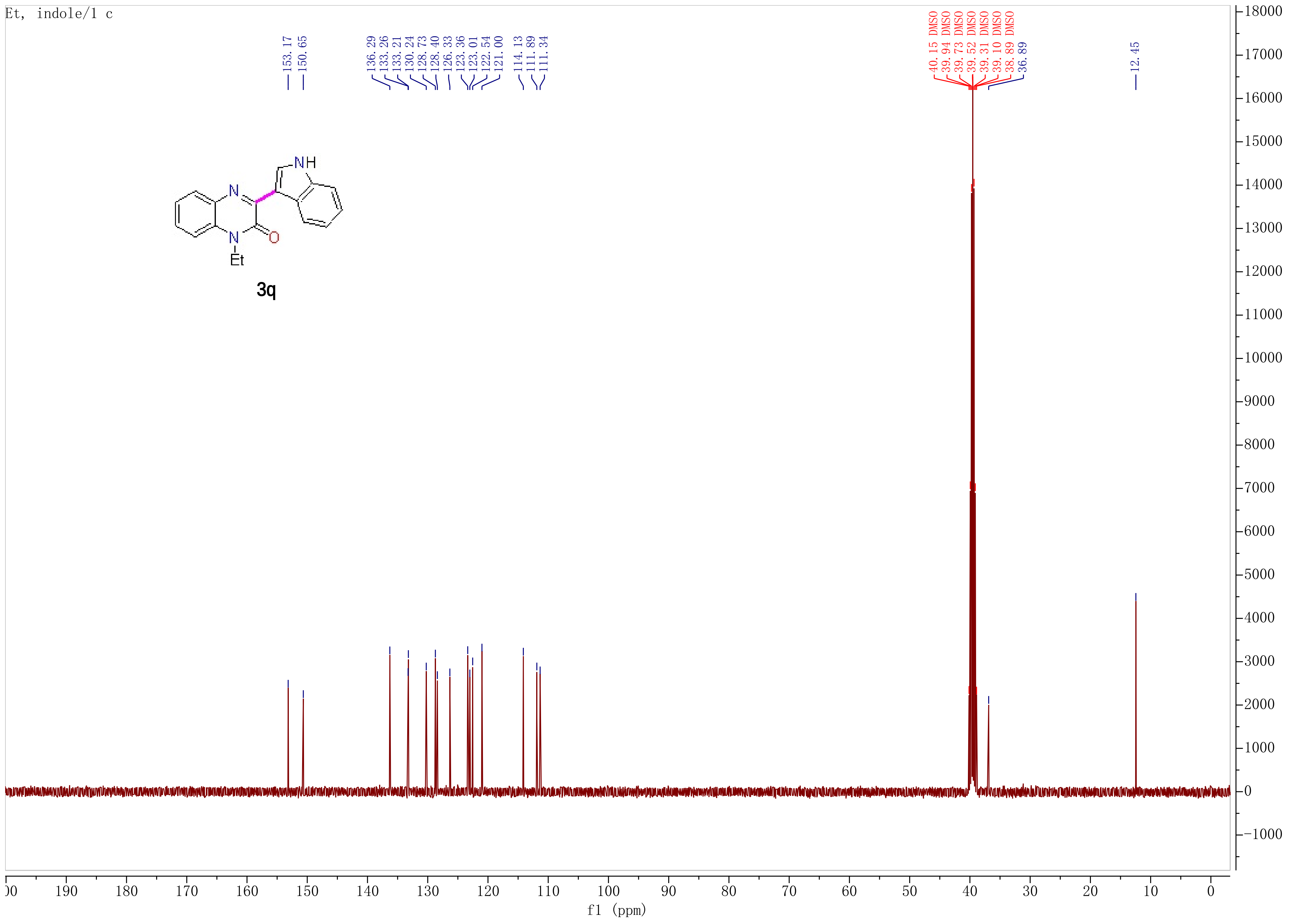
Et, indole/1 c
153.17
150.65
136.29
133.26
133.21
130.24
128.73
128.40
126.33
123.36
123.01
122.54
121.00
114.13
111.89
111.34
40.15 DMSO
39.94 DMSO
39.73 DMSO
39.52 DMSO
39.31 DMSO
39.10 DMSO
38.89 DMSO
36.89
12.45
NH
N
N
O
Et
3q
f1 (ppm)

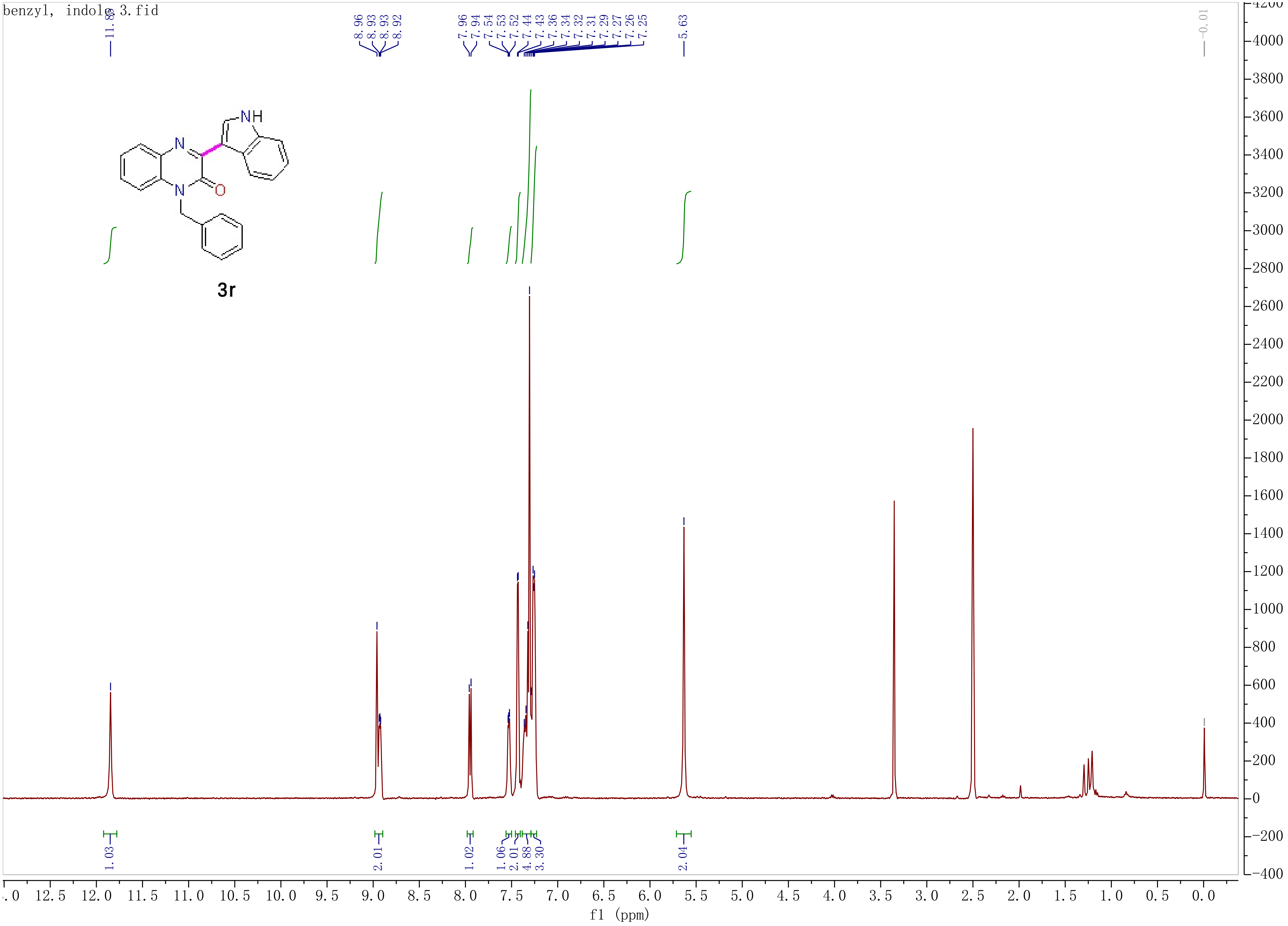

benzyl, indole 3.fid
11.83
8.96
8.93
8.93
8.92
7.96
7.94
7.54
7.53
7.52
7.44
7.43
7.36
7.34
7.32
7.31
7.29
7.27
7.26
7.25
5.63
-0.01
N
NH
N
O
3r
1.03
2.01
1.02
1.06
2.01
4.88
3.30
2.04
f1 (ppm)

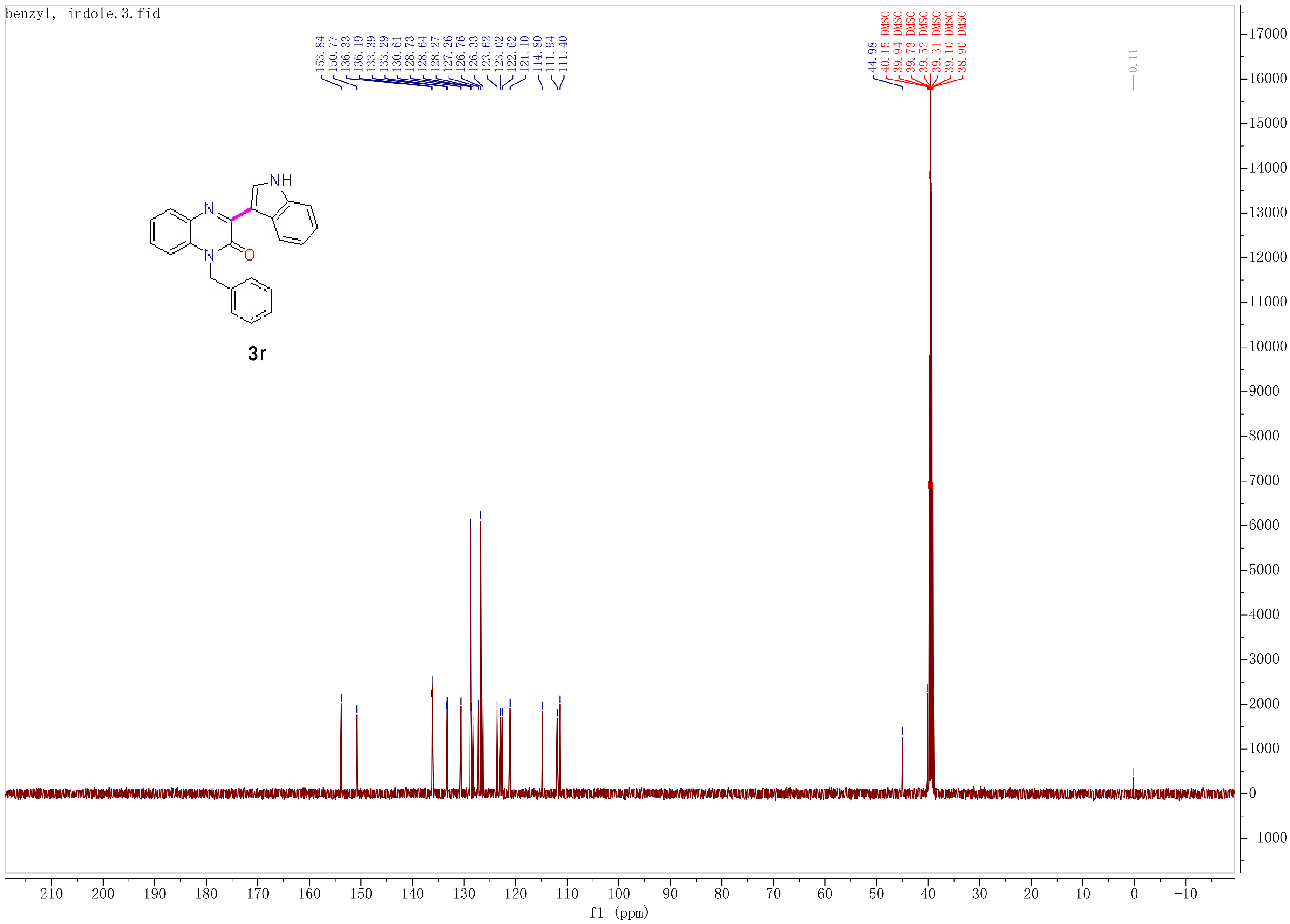

benzyl, indole.3.fid
153.84
150.77
136.33
136.19
133.39
133.29
130.61
128.73
128.64
128.27
127.26
126.76
126.33
123.62
123.02
122.62
121.10
114.80
111.94
111.40
44.98
40.15 DMSO
39.94 DMSO
39.73 DMSO
39.52 DMSO
39.31 DMSO
39.10 DMSO
38.90 DMSO
0.11
NH
N
N
O
3r
f1 (ppm)

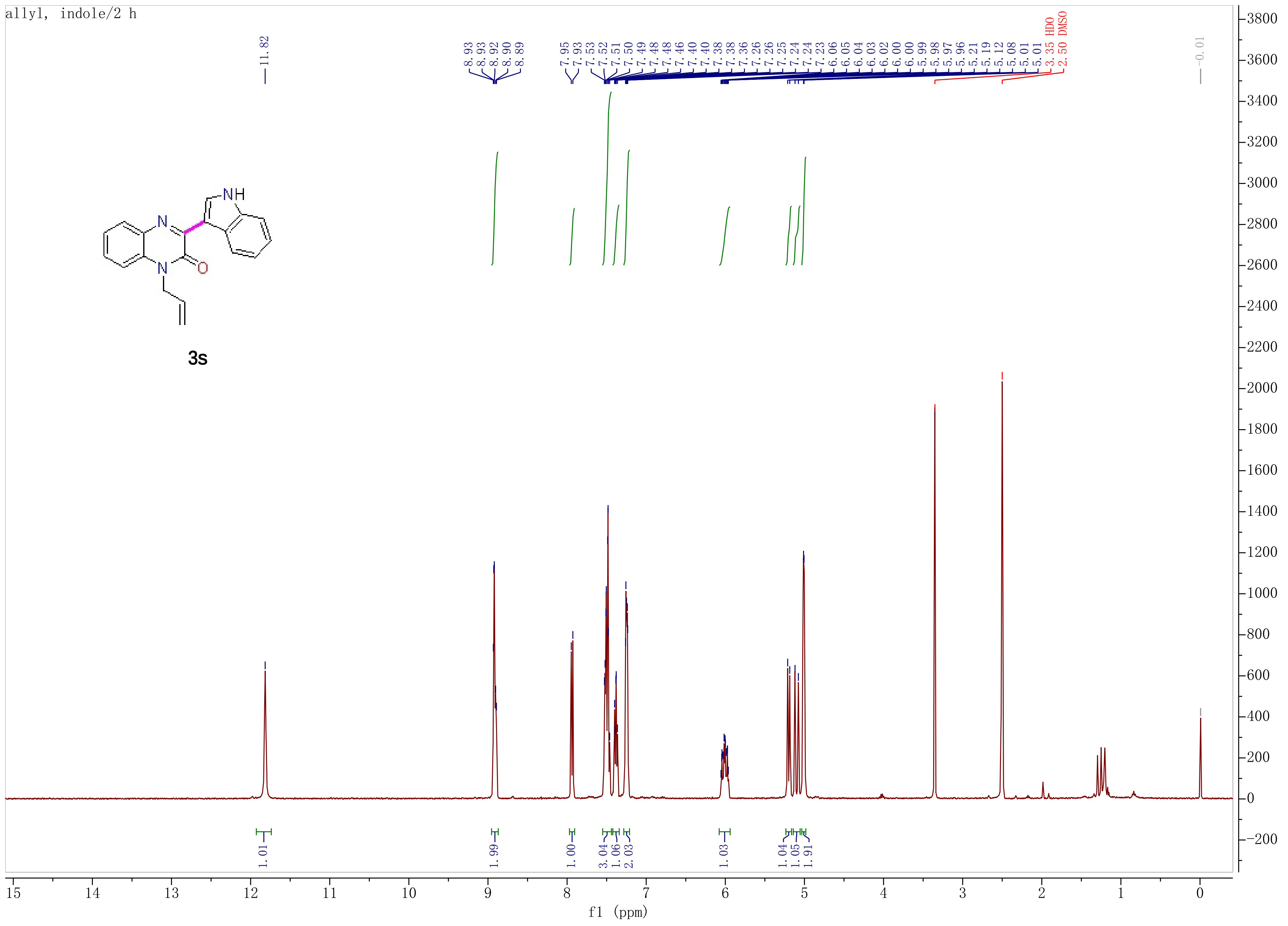
allyl, indole/2 h
11.82
8.93 8.93 8.92 8.90 8.89
7.95 7.93 7.53 7.52 7.51 7.50 7.49 7.48 7.48 7.46 7.40 7.40 7.38 7.38 7.36 7.26 7.26 7.25 7.24 7.24 7.23
6.06 6.05 6.04 6.03 6.02 6.00 6.00 5.99 5.98 5.97 5.96
5.21 5.19 5.12 5.08 5.01 5.01
3.35 HDO
2.50 DMSO
-0.01
NH
N
N
O
3s
1.01
1.99
1.00
3.04
1.06
2.03
1.03
1.04
1.05
1.91
f1 (ppm)

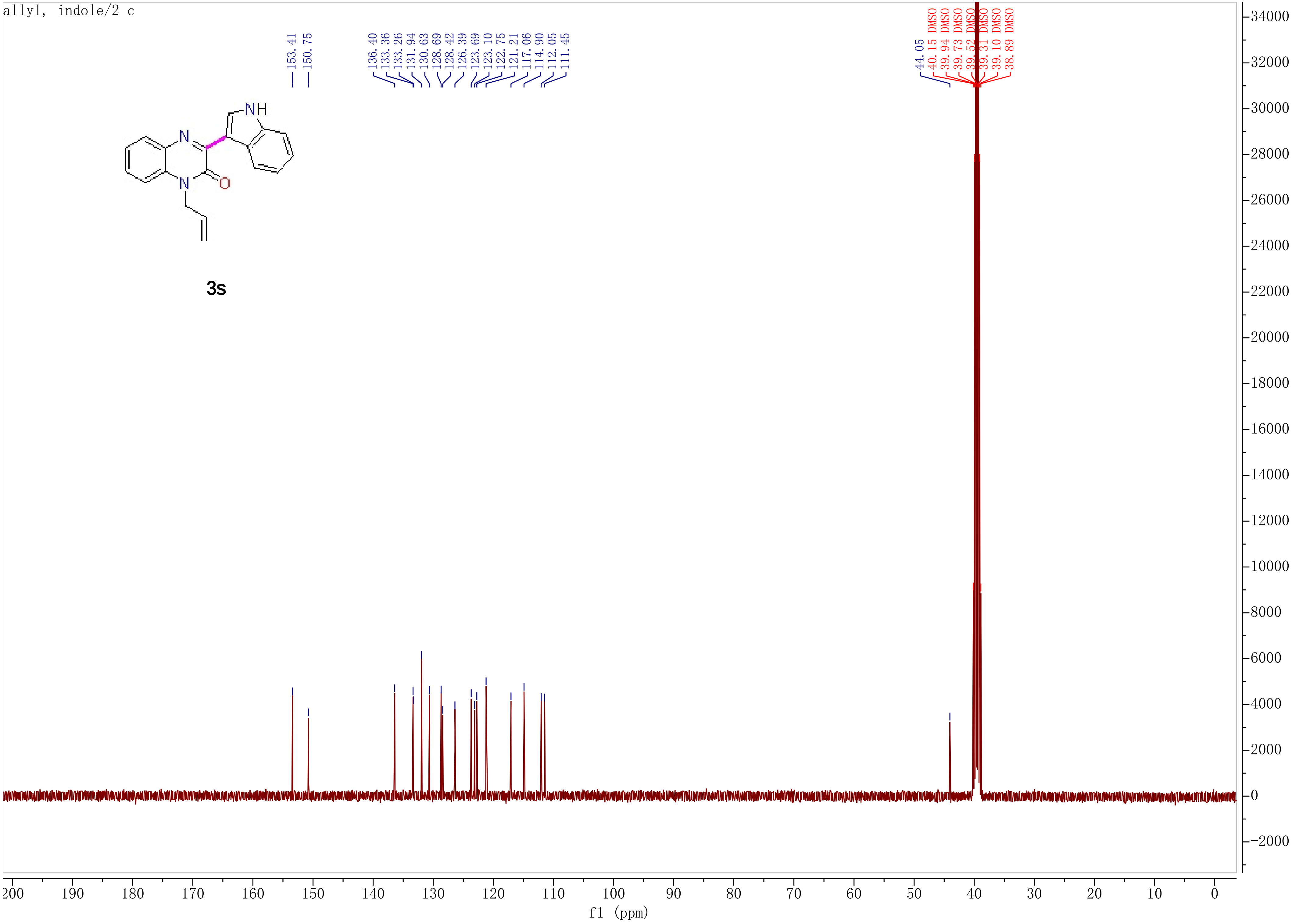

allyl, indole/2 c
153.41
150.75
136.40
133.36
133.26
131.94
130.63
128.69
128.42
126.39
123.69
123.10
122.75
121.21
117.06
114.90
112.05
111.45
44.05
40.15 DMSO
39.94 DMSO
39.73 DMSO
39.52 DMSO
39.31 DMSO
39.10 DMSO
38.89 DMSO
NH
N
N
O
3s
f1 (ppm)

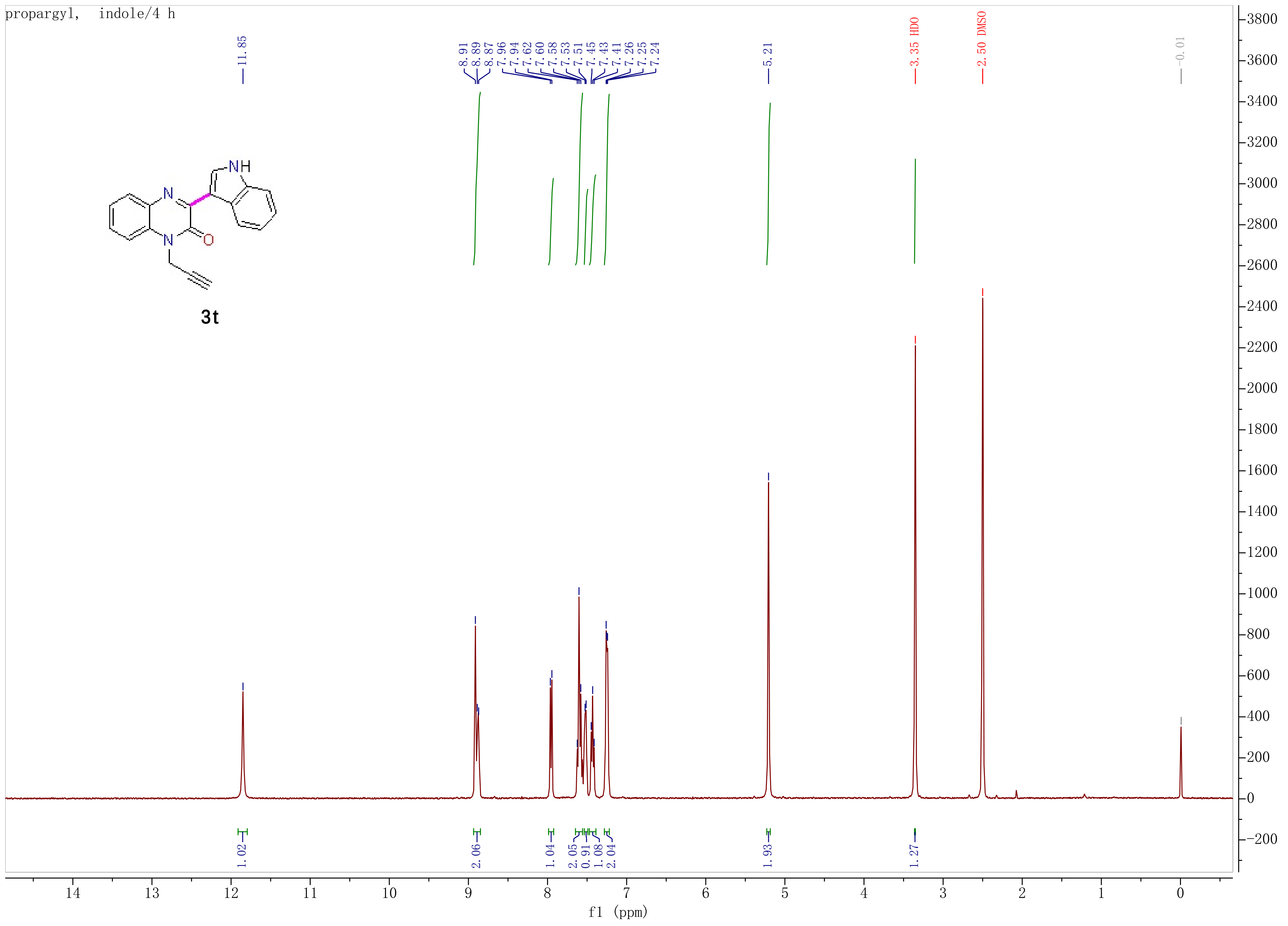

propargyl, indole/4 h
11.85
8.91
8.89
8.87
7.96
7.94
7.62
7.60
7.58
7.53
7.51
7.45
7.43
7.41
7.26
7.25
7.24
5.21
3.35 HDO
2.50 DMSO
-0.01
NH
N
N
O
3t
1.02
2.06
1.04
2.05
0.91
1.08
2.04
1.93
1.27
f1 (ppm)

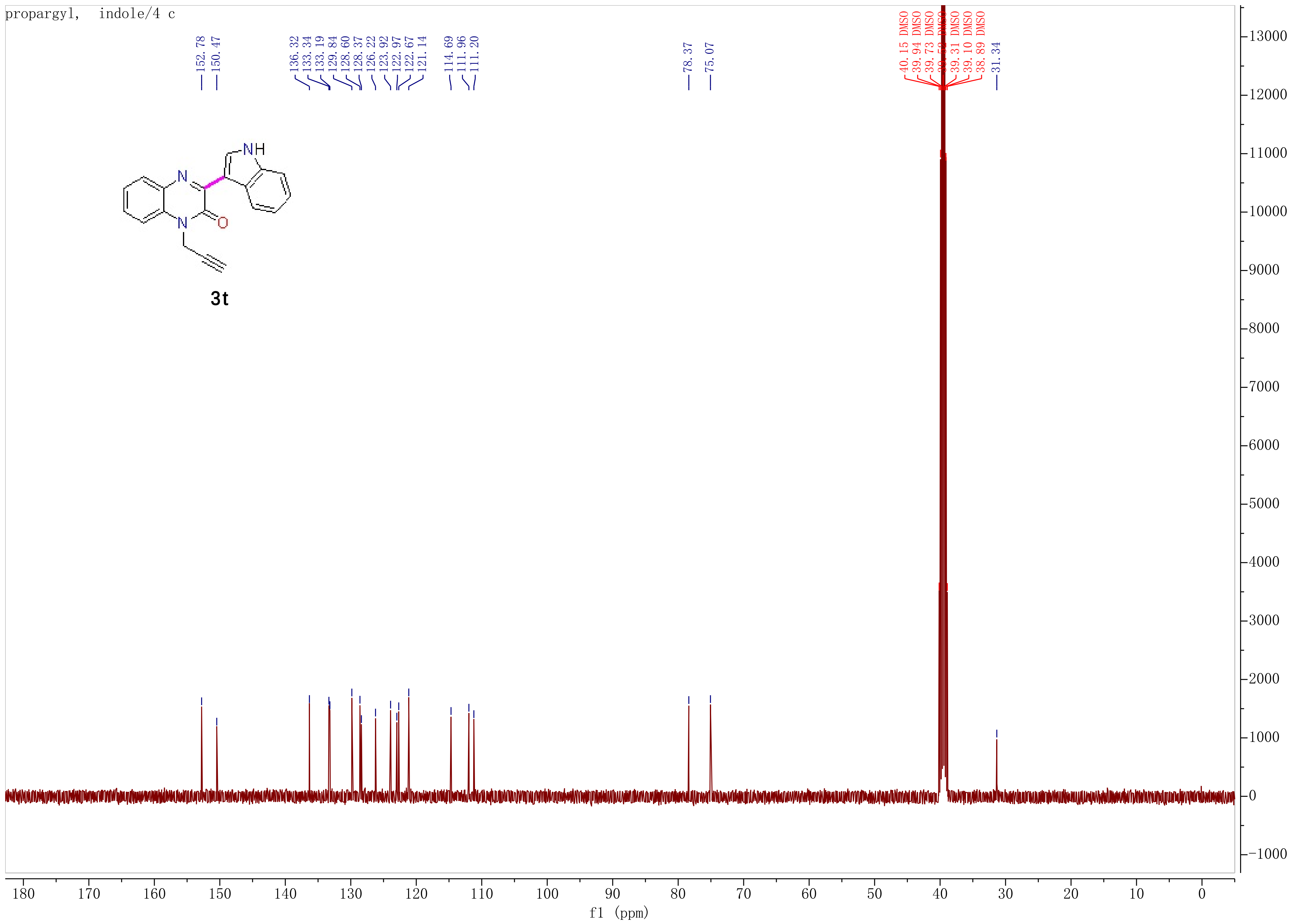

propargyl, indole/4 c
152.78
150.47
136.32
133.34
133.19
129.84
128.60
128.37
126.22
123.92
122.97
122.67
121.14
114.69
111.96
111.20
78.37
75.07
40.15 DMSO
39.94 DMSO
39.73 DMSO
39.31 DMSO
39.10 DMSO
38.89 DMSO
31.34
NH
N
N
O
3t
f1 (ppm)
